# Synthesis, crystal structure and *in-silico* evaluation of aryl­sul­fon­amide Schiff bases for potential activity against colon cancer

**DOI:** 10.1107/S205322962400233X

**Published:** 2024-03-28

**Authors:** Sherif O. Kolade, Oluwafemi S. Aina, Allen T. Gordon, Eric C. Hosten, Idris A. Olasupo, Adeniyi S. Ogunlaja, Olayinka T. Asekun, Oluwole B. Familoni

**Affiliations:** a Department of Chemistry, University of Lagos, Akoka-Yaba, Lagos, Nigeria; b Department of Chemistry, Nelson Mandela University, Port Elizabeth 6031, South Africa; University of the Witwatersrand, South Africa

**Keywords:** Schiff base, aryl­sul­fon­amide, colon cancer, crystal structure, mol­ecular docking, ADMET

## Abstract

This report presents a comprehensive investigation into the synthesis and characterization of Schiff base com­pounds derived from benzene­sul­fon­amide. The synthesis process, involved the reaction between *N*-cyclo­amino-2-sulf­anil­amide and various substituted *o*-salicyl­aldehydes, resulted in a set of com­pounds that were subjected to rigorous characterization using advanced spectral techniques, including ^1^H NMR, ^13^C NMR and FT–IR spectroscopy, and single-crystal X-ray diffraction. Furthermore, an in-depth assessment of the synthesized com­pounds was conducted through Absorption, Distribution, Metabolism, Excretion and Toxicity (ADMET) analysis, in conjunction with docking studies, to elucidate their pharmacokinetic profiles and potential. Impressively, the ADMET analysis showcased encouraging drug-likeness properties of the newly synthesized Schiff bases. These computational findings were substanti­ated by mol­ecular properties derived from density functional theory (DFT) calculations using the B3LYP/6-31G* method within the Jaguar Module of Schrödinger 2023-2 from *Maestro* (Schrodinger LLC, New York, USA). The ex­plor­ation of frontier mol­ecular orbitals (HOMO and LUMO) enabled the computation of global reactivity descriptors (GRDs), encompassing charge separation (*E*_gap_) and global softness (*S*). Notably, within this analysis, one Schiff base, namely, 4-bromo-2-{*N*-[2-(pyr­rol­idine-1-sul­fonyl)phenyl]car­box­imid­oyl}phenol, **20**, em­erged with the smallest charge separation (Δ*E*_gap_ = 3.5780 eV), signifying heightened potential for biological properties. Conversely, 4-bromo-2-{*N*-[2-(piper­idine-1-sul­fonyl)phenyl]car­box­imid­oyl}phenol, **17**, exhibited the largest charge separation (Δ*E*_gap_ = 4.9242 eV), implying a relatively lower propensity for biological activity. Moreover, the synthesized Schiff bases displayed re­marke­able inhibition of tankyrase poly(ADP-ribose) polymerase enzymes, integral in colon cancer, surpassing the efficacy of a standard drug used for the same purpose. Additionally, their bioavailability scores aligned closely with established medications such as trifluridine and 5-fluoro­uracil. The ex­plor­ation of mol­ecular electrostatic potential through colour mapping delved into the electronic behaviour and reactivity tendencies intrinsic to this diverse range of mol­ecules.

## Abstract 2

The successful synthesis, characterization and analysis of inter­molecular inter­actions of aryl­sul­fon­amide Schiff bases have been achieved, alongside their evaluation for inhibitory effects on tankyrase poly(ADP-ribose) polymerase in the context of colon cancer, through *in-silico* testing.

## Introduction

The quest for effective anti­cancer agents remains a pivotal challenge in medicinal chemistry and pharmacology, particularly in the context of colon cancer, which is among the leading causes of cancer-related mortality worldwide (Kumar *et al.*, 2023*a* ▸,*b* ▸). The synthesis of novel com­pounds and the ex­plor­ation of their biological activities are critical steps in the development of new therapeutic agents. In this connection, aryl­sul­fon­amide Schiff bases have emerged as a class of com­pounds with significant potential due to their versatile chemical structures and promising pharmacological profiles (Irfan *et al.*, 2020 ▸; Muhammad-Ali *et al.*, 2023 ▸; Dueke-Eze *et al.*, 2020 ▸).

Schiff bases, characterized by their imine functional group (–C=N–), have been studied extensively for their diverse pharmacological activities, including anti­cancer properties (Alblewi *et al.*, 2023 ▸). The introduction of an aryl­sul­fon­amide moiety into Schiff base structures has been hypothesized to enhance their biological activity, owing to the known efficacy of the sul­fon­amide group in various therapeutic agents (Abd El-Wahab *et al.*, 2020 ▸; Elsamra *et al.*, 2022 ▸). The rationale behind this hypothesis centres on exploring the anti­cancer potential of Schiff bases, particularly focusing on their inter­action with colon cancer. Extensive literature has already underscored their efficacy as anti­cancer agents (Abd-Elzaher *et al.*, 2016 ▸). Remarkably, Schiff bases have demonstrated activity against colon cancer and have been documented for such effects (Matela, 2020 ▸). Notably, the combination of benzene­sul­fon­amide with a Schiff base has been reported, merging the bioactive attributes of sul­fon­amides and Schiff bases to investigate potential synergies between these well-established functional groups (Afsan *et al.*, 2020 ▸). The cumulative evidence of their enhanced activity spurred our inter­est in undertaking the present study.

**Figure 1:**
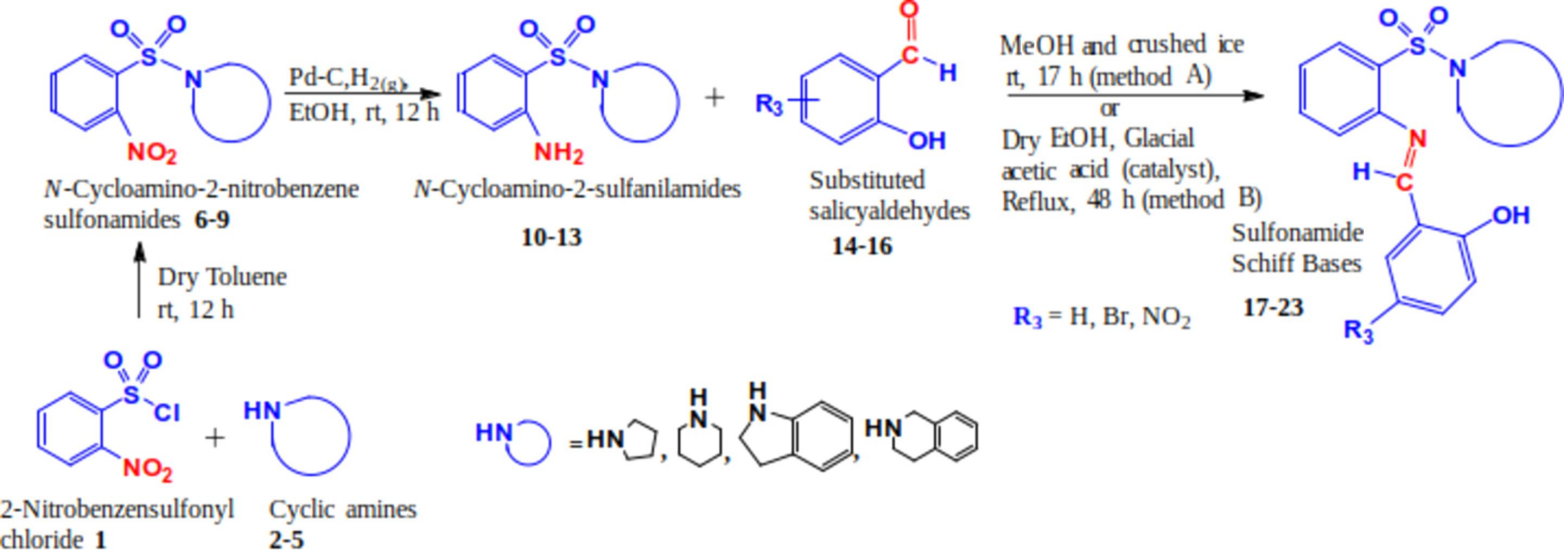
General reaction scheme of the formation of potentially bioactive sul­fon­amide Schiff bases.

Tankyrase poly(ADP-ribose) polymerase, a crucial enzyme involved in DNA repair and the regulation of various cellular processes, has been implicated in the development of colon cancer (Feng & Koh, 2013 ▸; Eisemann & Pascal, 2020 ▸). Tankyrase’s involvement in Wingless-related integration site (Wnt) signaling, which governs cell growth, motility and differentiation, makes it a significant target (Pai *et al.*, 2017 ▸). Colorectal cancer, a prevalent form of cancer worldwide, often arises from precancerous polyps in the colon or rectum. Tankyrase’s modification of Axin through poly(ADP-ribose) chains disrupts the Axin complex, leading to Axin degradation and β-catenin stabilization. The accumulation of β-catenin contributes to the progression of colon cancer (Gao *et al.*, 2014 ▸). Under normal circumstances, Axin aids in regulating the Wnt pathway by facilitating β-catenin degradation (Huang & He, 2008 ▸). However, mutations in colon cancer can lead to the persistent accumulation of β-catenin, even in the absence of Wnt signaling, promoting uncontrolled cell growth and tumour formation (Behrens, 2000 ▸). Inhibiting specific amino acid residues in human tankyrase poly(ADP-ribose) polymerase using Schiff bases, such as (*E*)-2-[(2-hy­droxy­benzyl­idene)amino]­benzene­sul­fon­amide derivatives (**17**–**23**) (Fig. 1 ▸), could potentially prevent the accumulation of β-catenin, holding promise for effective inter­vention (Meyer *et al.*, 2006 ▸).

Our research also delves into the crystal structure of benzene­sul­fon­amides (Kolade *et al.*, 2020 ▸), which, along with our ex­plor­ation of the crystal structure of a Schiff base sul­fon­amide, forms a comprehensive investigation into the potential therapeutic avenues these com­pounds may offer.

## Experimental

### Instruments and measurements

All reagents were purchased from Millipore Sigma (Ger­many and South Africa) and were used without further purification. The melting points were determined on an elec­tro­thermal digital melting-point apparatus and are uncorrected. Reactions were monitored by thin-layer chromatography (TLC) on Merck silica gel 60 F254 precoated plates using a di­chloro­methane/*n*-hexane (2 or 1.4:1 *v*/*v*) solvent system vis­ualized under a UV lamp (254 nm). Column chromatography was per­formed with silica gel (70–230 mesh ASTM) and mobile phases were as indicated. Sample crystallization was achieved by the slow evaporation of the indicated solvent systems at ambient temperature. IR spectra were obtained using a Bruker Tensor 27 platinum ATR–FT–IR spectrometer. The ATR–FT–IR spectra were acquired in a single mode with a resolution of 4 cm^−1^ over 32 scans in the region 4000–650 cm^−1^. ^1^H and ^13^C NMR spectra were record­ed in CDCl_3_ on a Bruker 400 MHz spectrometer. Chemical shift values were measured in parts per million (ppm) downfield from tetra­methyl­silane (TMS), and coupling constants (*J*) are reported in Hertz (Hz). Theoretical studies were per­formed for the com­pounds and, in each case, their single-crystal X-ray diffraction (SC-XRD) structures were used for optimization and global reactivity descriptor (GRD) calculations.

### Synthesis and crystallization

#### Synthesis of sul­fon­amide Schiff bases

The general reaction scheme for the formation of potentially bioactive sul­fon­amide Schiff bases is shown in Fig. 1 ▸. The *N*-cyclo­amino-2-sulf­anil­amides were prepared as reported previously (Kolade *et al.*, 2022 ▸) by reacting the amino­sulf­anil­amides with substituted *o*-salicyl­aldehyde either at room temperature or under reflux to obtain the required Schiff bases in good yields (57–80%). Only *o*-salicyl­aldehyde and *N*-piperidinyl-2-sulf­anil­amide gave the required Schiff base at room temperature, and the others were refluxed to give the required products.

*2.2.1.1. Method A: synthesis of N^1^-(2^1^-hy­droxy­benzyl­iden­yl)-N-piperidinyl-2-sulf­anil­amide **17***. *N*-Piperidinyl-2-sulf­anil­amide **11** (0.100 g, 0.417 mmol) was dissolved in methanol (5 ml) and 2-hy­droxy­benzaldehyde, or *o*-salicyl­aldehyde, **14** (0.056 g, 0.05 ml, 0.459 mmol), was added dropwise to the solution with stirring. Crushed ice (1.00 g) was added to the stirring mixture after 5 min. The reaction mixture was stirred at ambient temperature for 17 h and monitored with TLC. On completion, the mixture was filtered, using a Buckner funnel, and the residue was air-dried, dissolved in warm methanol and filtered hot to leave single crystals of **17** on slow evaporation. The physical properties and the spectroscopic data are pre­sent­ed in the supporting information.

*2.2.1.2. Method B: synthesis of N^1^-(5^1^-bromo/nitro-2^1^-hy­droxy­benzyl­iden­yl)-N-cyclo­amino-2-sulf­anil­amides **18**–**23***. To a stirring solution of *N*-cyclo­amino-2-sulf­anil­amides **10**–**13** (1.0 mmol) in ethanol (10 ml) was added 5-bromo­(nitro)-*o*-salicyl­aldehydes **15**–**16** (1.3 mmol), followed by glacial acetic acid (10 drops) as catalyst. The whole mixture was refluxed for 48 h and monitored with TLC. After completion, the reaction mixture was allowed to cool to ambient temperature and kept in the fume hood for 24 h. The residue was then recrystallized from ethanol (10 ml) and filtered hot to leave crystals of **18**–**23** on slow evaporation. The physical properties and the spectroscopic data are pre­sent­ed in the supporting information.

### Docking studies

#### Selection of reference drugs and cancer protein macromolecule

Common anti­cancer standard drugs, such as capecitabine (ID: 60953), 5-fluoro­uracil (ID: 3385) and trifluridine (ID: 6256), were downloaded from *Pubchem* (https://pubchem.ncbi.nlm.nih.gov/, last accessed on May 25, 2023) and saved in .sdf format as reference to compare inhibitory per­formance with the synthesized chemical com­pounds. In order to evaluate the lead com­pounds as inhibitors of the tankyrase poly(ADP-ribose) polymerase family responsible for cancer pathogenesis (Shirai *et al.* 2020 ▸), its protein crystal structure (PDB entry 6kro) was downloaded from www.rcsb.org (last accessed on April 20, 2023).

#### Preparation of ligands, reference drugs and protein mol­ecules for docking

Synthesized com­pounds (drawn using *Chemdraw* 14.0 and saved in .sdf format) and the selected reference drugs saved as .sdf files were opened in PyRx 0.8 *Autodock Vina* software (Kondapuram *et al.*, 2021 ▸). Energy minimization was car­ried out, followed by conversion into protein databank partial charge (pdbqt) ligands. The crystal structure of the protein mol­ecule tankyrase poly(ADP-ribose) polymerase at a resolution of 1.90 Å was also uploaded into *BIOVIA Discovery Studio* (Dassault Systémes, 2020 ▸). The binding sites were determined and all unwanted heteroatoms and water mol­ecules were removed, while polar hydrogen bonds were added to give pure protein and saved as .pdb files (Pawar & Rohane, 2021 ▸).

#### Mol­ecular docking

Docking simulations were per­formed with PyRx *AutoDock* using the Lamarkian genetic algorithm and default procedures for docking a flexible ligand to a rigid protein. Blind docking was initially per­formed to identify all potential binding sites on the target protein within a 90 × 75 × 75 cubic grid centre. A grid spacing of 1.00 Å was used for the calculation of the grid maps using the autogrid module of *AutoDock* tools. For each ligand, a set of nine independent runs were per­formed for the enzyme run against all ligands and reference drugs. Clear identification of the potential binding sites is followed by docking of ligands to the sites and the most probable and energetically favourable binding conformations were determined (Trott & Olson, 2010 ▸). Docking solutions were analyzed and ranked on the basis of the *Vina* scoring functions. All calculations were car­ried out on PC-based machines running Microsoft Windows 10 operating systems. The resulting structures were vi­su­al­ized and analyzed using the *Discovery Studio* visualizer.

### Refinement

Crystal data, data collection and structure refinement details are summarized in Table 1 ▸. Carbon-bound H atoms were added in idealized geometrical positions in a riding model. Nitro­gen-bound H atoms were located in a difference map and refined freely. The H atom of the hy­droxy group was allowed to rotate with a fixed angle around the C—O bond to best fit the experimental electron density. The Hirshfeld surface analyses were per­formed with *CrystalExplorer* (Version 21.5; Spackman *et al.*, 2021 ▸).

## Results and discussion

### Chemistry

*N* ^1^-(5^1^-Substituted-2^1^-hy­droxy­benzyl­iden­yl)-*N*-cyclo­amino-2-sulf­anil­amides **17**–**23** were prepared from the reaction of *N*-cyclo­amino-2-sulf­anil­amides **10**–**13** [as reported previously by Kolade *et al.* (2022 ▸)] with substituted *o*-salicyl­aldehyde either at room temperature or under reflux to obtain the required Schiff bases in good yields (57–80%). Only *o*-sali­cyl­al­de­hyde and *N*-pi­peri­din­yl-2-sulf­anil­amide gave the re­quired Schiff base at room temperature, and the others were refluxed to obtain the desired products. The *N* ^1^-(2^1^-hy­droxy­benzyl­iden­yl)-*N*-piperidinyl-2-sulf­anil­amide **17**, which was pre­pared at room temperature (Method A), was aimed at establishing an eco-friendly protocol. The reaction progress was monitored by TLC.

All the com­pounds synthesized were characterized by their melting points and their IR, ^1^H/^13^C NMR and MS spectra. In order to clarify the mode of bonding on the ligands, their IR spectra (as pre­sent­ed in the supporting information) confirm the formation of the sul­fon­amide Schiff base ligands **17**–**23** by the appearance of a strong absorption band at around 1614–1618 cm^−1^, which is attributed to stretching vibrations of the azomethine group and the absence of the original aldehydic bond (–C=O) and NH_2_ vibrations (Salehi *et al.*, 2019 ▸). The stretching vibrations of aromatic carbon-to-carbon double bonds (C=C) of the com­pounds are observed at 1512–1570 cm^−1^, while the strong absorption bands which appeared at around 1300–1331 and 1119–1155 cm^−1^ are indexed to (S=O)_2_ asymmetric and symmetric stretching frequencies, respectively. The IR spectra provided in the supporting information also reveal other diagnostic bands that further corroborate the formation of Schiff base ligands.

The ^1^H NMR spectra (see supporting information) of sul­fon­amide Schiff bases (ligands) **17**–**23** were record­ed in CDCl_3_, using tetra­methyl­silane (TMS) as the inter­nal standard. The signals at 1.36–4.46 ppm in the ^1^H NMR spectra of the ligands result from the protons of the methyl­ene groups (–C**H**_2_–). The singlet signals which correspond to the imine groups (–C**H**=N–) in these ligands are observed at 8.12–8.67 ppm. The phenolic protons (–OH), the most deshielded protons, are clearly indicated at 12.31–13.50 ppm. The deshielded nature of the phenolic OH hydrogen is likely a consequence of it forming a strong resonance-assisted intramolecular O—H⋯N hydrogen bond. The aromatic protons of the com­pounds are record­ed in the range 6.66–8.44 ppm. Finally, the success of the formation of the sul­fon­amide Schiff bases is corroborated by the ^13^C NMR spectra (see supporting information) of com­pounds **17**–**23**, which show the azomethine C atoms (–**C**=N–) at the chemical environments of 160–162 ppm, while the most deshielded phenolic C atoms occur at 163–166 ppm and the aromatic C atoms are observed at 111–148 ppm.

### Crystal structure

Compound **18** formed pale-yellow platelets with the ortho­rhom­bic space group *Pbca* (Table 1 ▸). The close *ortho* positioning of the two functional groups on the central arene ring forces their rotation, with a resulting dihedral angle of 30.8 (2)° for the least-squares planes through the piperidine and the imino­methyl­phenol groups, and dihedral angles of 77.17 (17) and 51.82 (12)°, respectively, with the central linking arene group. The imino­methyl­phenol group is planar, with an intra­molecular O—H⋯N inter­action of 1.86 Å, forming a ring closure that can be described with an *S*(6) graph-set descriptor (Table 2 ▸). The inter­molecular hydrogen-bond inter­actions are dominated by the O atoms from the sulfonyl group, with a number of C—H⋯O=S inter­actions, resulting in three chains of inter­actions having *C*(7), *C*(9) and *C*(7) descriptors. The Hirshfeld surface illustrated in Fig. S1 (see supporting information) clearly shows these inter­actions. The hy­droxy group also contributes and is involved in a C—H⋯O inter­action of 2.54 Å, resulting in a *C*(8) chain inter­action. The presence of these C—H⋯O inter­actions is indicated on the Hirshfeld surface fingerprint plot as H⋯O (see Fig. S2). There is also an inter­molecular C—H⋯π(ring) inter­action of 2.88 Å to the centroid of the C11–C16 ring. This inter­action is indicated by H⋯C on the Hirshfeld surface fingerprint plot (Fig. S2). Table S3 (see supporting information) lists the percentage reciprocal hydrogen surface contact areas, with the H⋯H inter­actions having the largest percentage contact. The closest H⋯H contact indicated in Fig. S2 arises between H atoms on the arene rings between two ad­ja­cent imino­methyl­phenol groups. The H⋯O/O⋯H and H⋯C/C⋯H inter­actions have similar contact surface areas, while H⋯Br/Br⋯H inter­actions are also present.

### Theoretical calculations

#### DFT calculations

Mol­ecular orbital calculations, en­com­pas­sing full geometry optimization, were methodically conducted on the Schiff base derivatives alongside the established pharmaceutical com­pounds trifluridine, capecitabine and 5-fluoro­uracil. These sophisticated calculations were executed using the Jaguar module within *Maestro* (Version 13.6.122) and *MMshare* (Version 6.2.122, Release 2023-2). This involved the integration of the basis set 6-31G* level, harmonized with the hybrid density functional theory (DFT) that incorporates the Becke 3-parameter exchange potential (Becke, 1993 ▸; Prokopenko *et al.*, 2019 ▸; Jędrzejczyk *et al.*, 2022 ▸; Pandi *et al.*, 2022 ▸). This intricate approach paved the way for the meticulous determination of crucial mol­ecular properties. The focus of this investigation involved the precise computation of the highest occupied mol­ecular orbital (HOMO) and the lowest unoccupied mol­ecular orbital (LUMO) using the aforementioned methodology. The outcomes of these meticulous calculations, which illuminate the intricate electronic structure and reactivity of the mol­ecules, served as the foundation for the sub­se­quent computation of pivotal global reactivity descriptors. These encompass a spectrum of descriptors, notably the ionization po­ten­tial (*I*), electron affinity (*A*), chemical po­ten­tial (μ), electronegativity (χ), global hardness (η), global softness (*S*) and global electrophilicity (ω) values (Gordon *et al.*, 2022 ▸).

#### Global reactivity descriptors of the synthesized Schiff bases and standard drugs

Density functional theory (DFT) stands as a widely embraced technique for *ab-initio* assessments of diverse mol­ecular components. Among its manifold utilities, it holds prominence in discerning the characteristics of frontier mol­ecular orbitals (FMOs), a pivotal factor in elucidating various reaction types and predicting the most reactive sites within conjugated systems (da Silva *et al.*, 2006 ▸). This comprehension of structure–property relationships assumes paramount importance in the endeavour to craft enhanced pharmaceutical agents, given that the mol­ecular configuration profoundly influences the per­formance of drugs (Mahmood *et al.*, 2022 ▸).

The bedrock for global vital reactivity descriptors lies in the FMO properties, precisely, the HOMO and LUMO energy values. Through a judicious application of DFT energetics, this study delved into the intricate tapestry of three-dimensional electronic states intrinsic to the mol­ecules under scrutiny. As such, the analysis offered an unprecedented glimpse into the transferability of lone pairs, the nuances of bond inter­actions, and the reactivity landscape within the specific mol­ecular milieu (Hall *et al.*, 2009 ▸). In its totality, this exhaustive computational inquiry provides an illuminating vista into the intricate electronic characteristics and reactivity proclivities of the mol­ecules under examination. The insights gleaned from this study significantly enrich our understanding of their potential roles and behaviours across a spectrum of chemical scenarios.

In the present context, the com­pounds under scrutiny underwent a meticulous ex­plor­ation of their quantum chemi­cal attributes. Specifically, the focus was on the localization energies of the HOMO and the LUMO. These energies, encapsulated within the rubric of FMOs, serve as linchpins in upholding chemical stability. Moreover, they emerge as potent tools for dissecting donor–acceptor inter­actions. The HOMO embodies a mol­ecule’s capacity to donate an electron, while the LUMO signifies its propensity to accept an electron. A lower LUMO value indicates an augmented inclination for electron acceptance, while higher HOMO values delineate a heightened disposition to donate electrons to unoccupied mol­ecular orbitals (Yele *et al.*, 2021 ▸). Considering the context of the LUMO within the synthesized Schiff bases, **19** displays the most intriguing attribute, featuring the lowest energy level for the LUMO orbital (−2.4963 eV), indicative of its pronounced tendency to accept electrons. Conversely, **17** showcases the highest LUMO energy level (−1.3287 eV). This establishes an order of increasing electron-accepting tendency among the com­pounds: **19** > **20** > **23** > **21** > **18** > **22** > **23** > capecitabine > trifluridine > **17** > 5-fluoro­uracil (Table 3 ▸).

On a contrasting note, when focusing on *E*_HOMO_, **20** de­mon­strates the highest energy level (−5.9851 eV), followed closely by **22** with the second highest *E*_HOMO_ (−6.1778 eV). These values signify the pronounced potential of **20** and **22** to donate electrons (Yele *et al.*, 2021 ▸). Remarkably, capecitabine emerges as the only standard drug displaying HOMO energies higher than the synthesized com­pounds (**19** and **23**), boasting an energy level of *E*_HOMO_ = −6.4578 eV. Beyond mere energy levels, a thorough structural analysis encompasses a comprehensive evaluation of intra-ligand inter­actions. Notably, these inter­actions include hydrogen bonds (within a 3.5 Å range), halogen bonds (within a 3.5 Å range), π–π stacking (within a 5.5 Å range) and π–cation inter­actions (within a 6.6 Å range).

**Figure 2:**
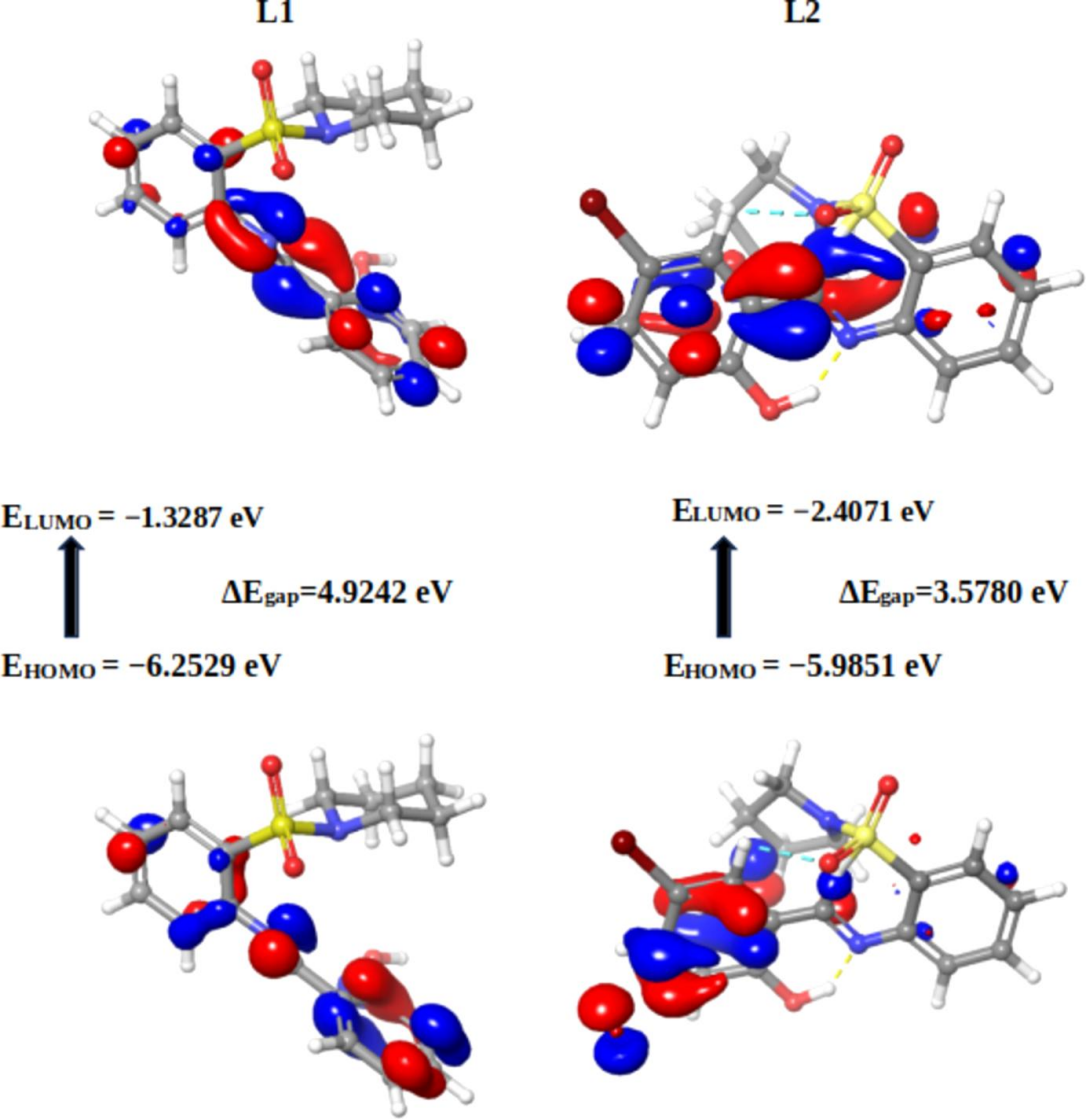
Frontier mol­ecular orbitals (FMOs) of **17** and **20**.

Delving deeper into the mol­ecular architecture, both the HOMO and the LUMO orbitals exhibit localization on both arene rings of **17**. However, no discernible hydrogen bonds or π–π stacking within the studied distances are exhibited by the com­pound. In the intricate case of **20**, HOMO orbitals predominantly localize on the arene ring bearing the Br atom, while the LUMO orbitals are positioned closer to the imine functionality (C=N). This arrangement leads to the identification of hydrogen bonding within the optimized structure of **20**, specifically involving C=N⋯OH (1.50 Å) and a weaker S=O⋯H(aromatic) inter­action (Fig. 2 ▸).

**Figure 3:**
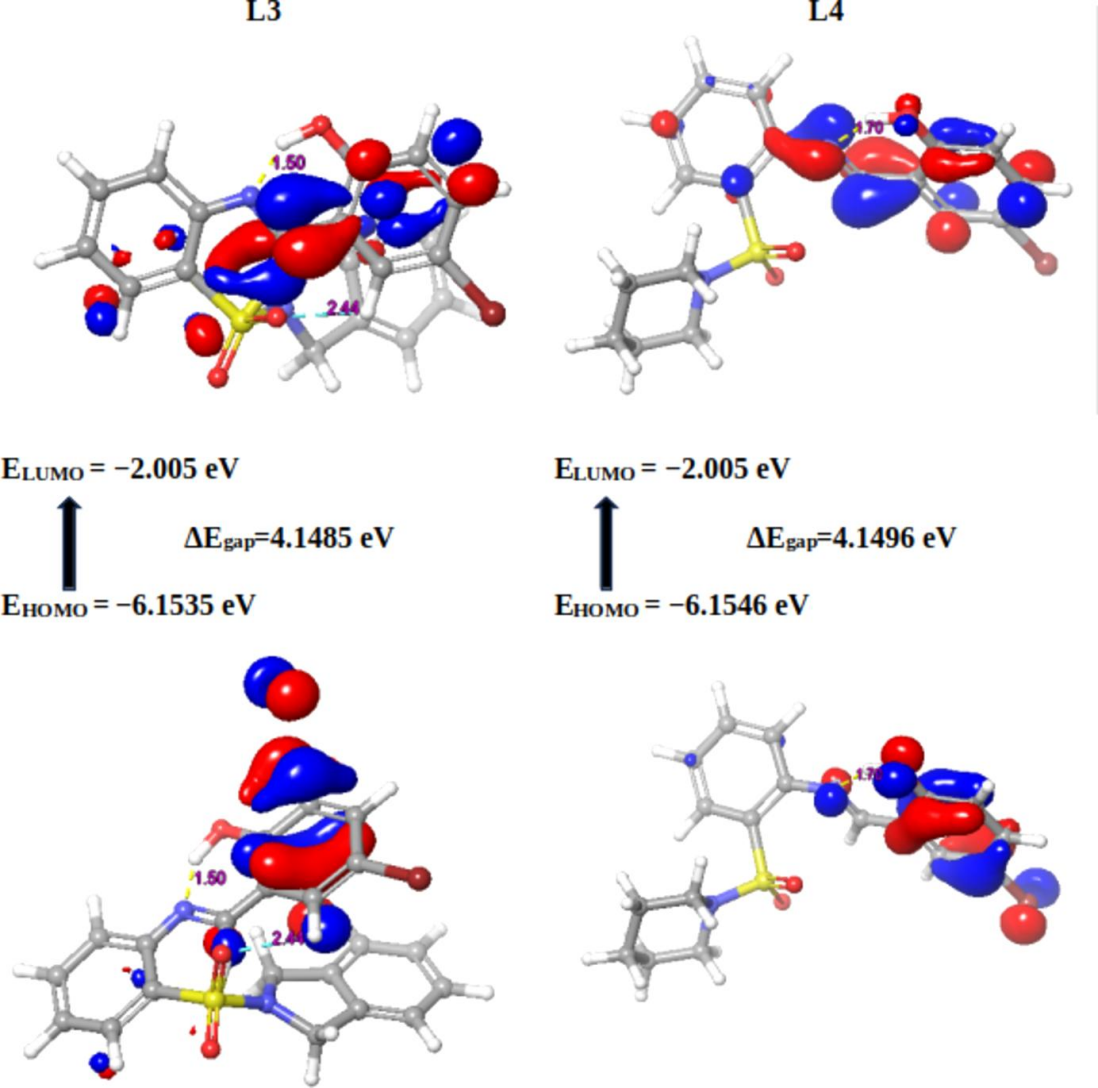
Frontier mol­ecular orbitals (FMOs) of **21** and **18**.

**Figure 4:**
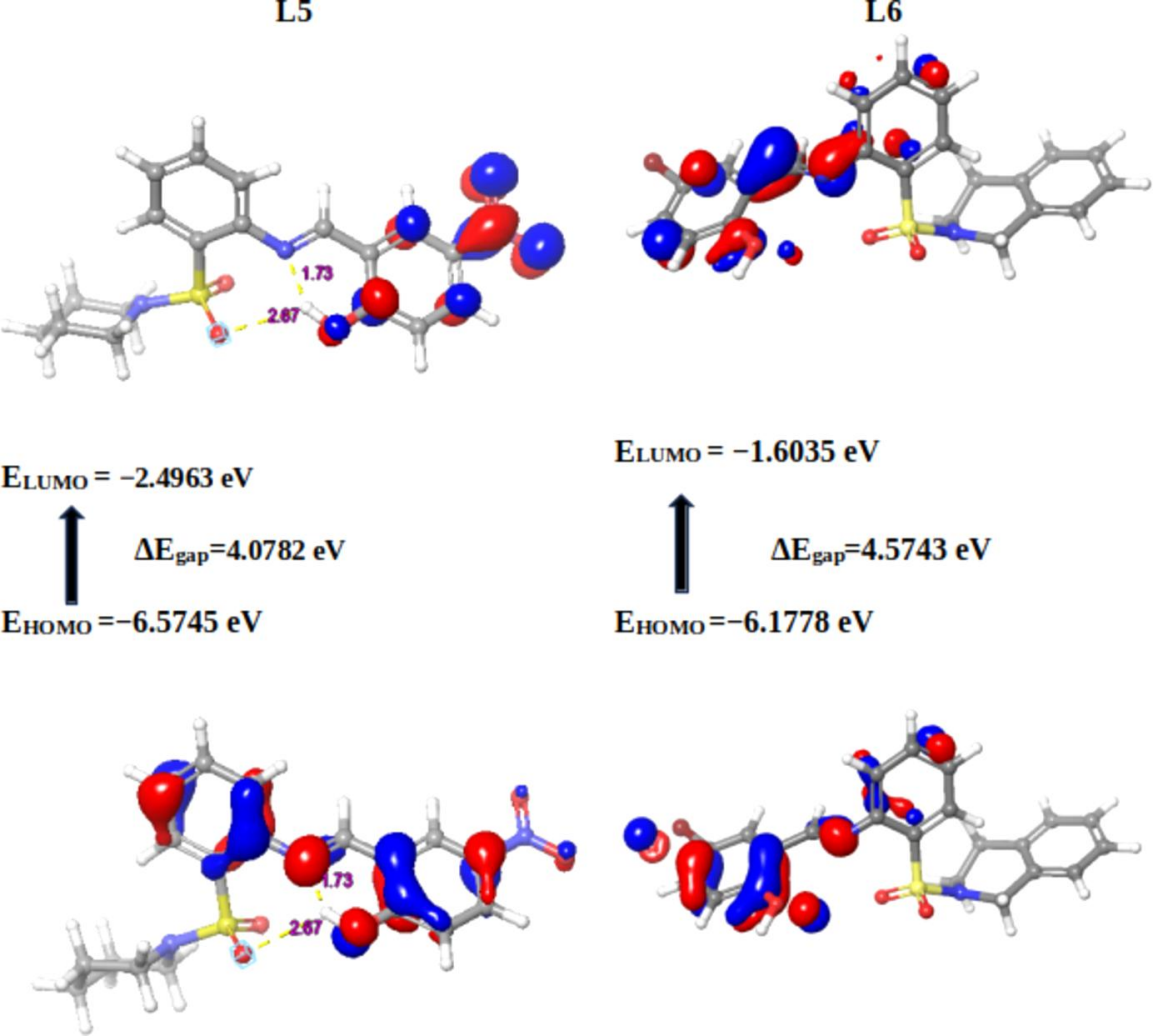
Frontier mol­ecular orbitals (FMOs) of **19** and **22**.

**Figure 5:**
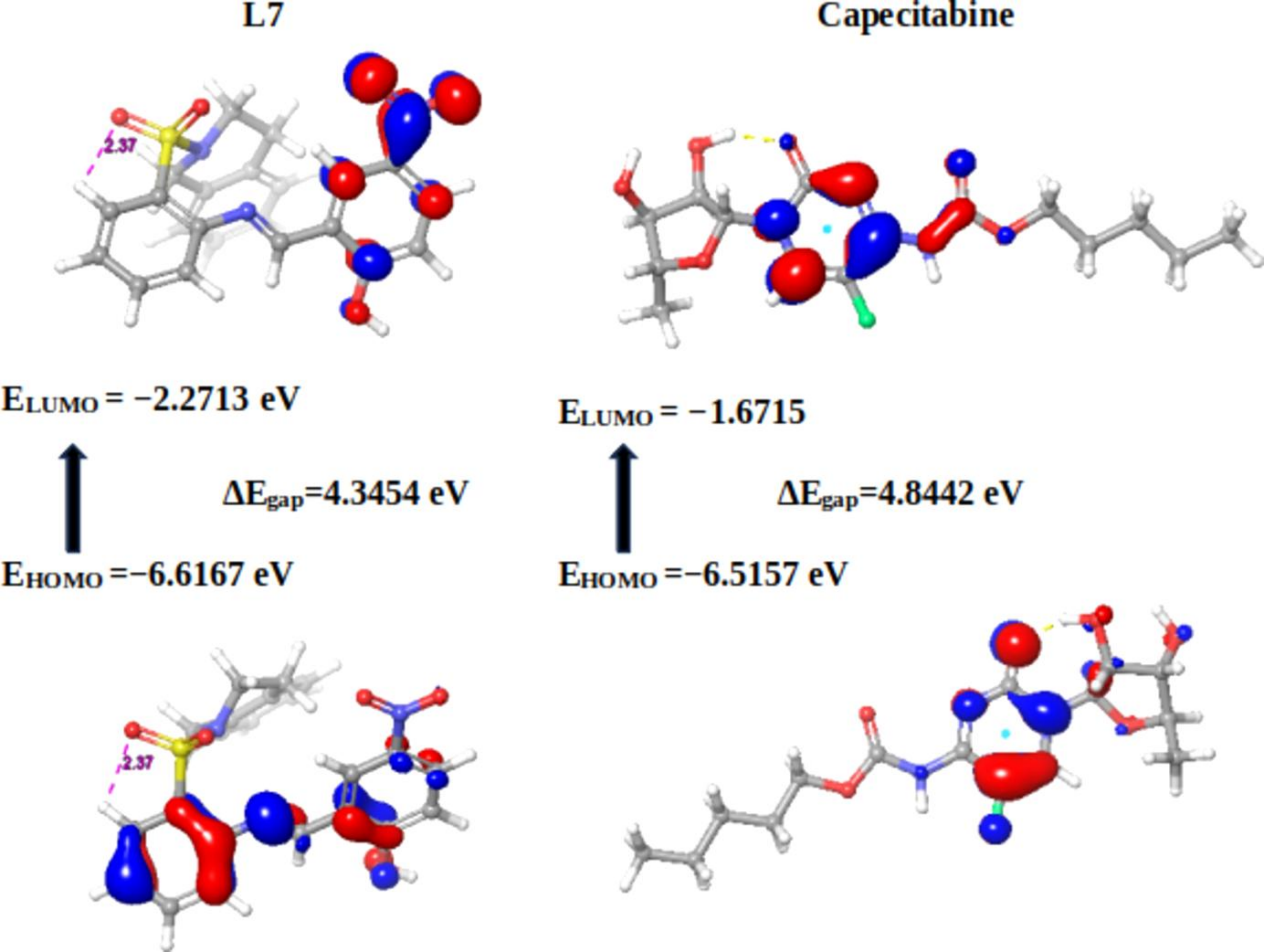
Frontier mol­ecular orbitals (FMOs) of **23** and capecitabine.

Similarly, Schiff base **21** (Fig. 3 ▸) showcases HOMO orbitals predominantly on the bromine-bearing arene ring, while the LUMO orbitals position themselves in proximity to the imine functionality. Notably, **21** boasts robust hydrogen bonding between the hy­droxy O and imine N atom (1.50 Å). Extending this pattern, derivatives **18** and **19** exhibit comparable hydrogen bonding to **21**, with distances of 1.70 and 1.73 Å, respectively. Schiff base **19** (Fig. 4 ▸) distinguishes itself further by showcasing an additional, albeit weaker, hydrogen bond (2.67 Å) between the S=O group and an aromatic H atom. The thematic consistency in the HOMO and LUMO orbital localization is mirrored across Schiff bases **18**, **19**, **22** and **23**, closely resembling the pattern exhibited by **20**, except for **23**, where the LUMO orbitals predominantly localize on the NO_2_ group (Fig. 5 ▸).

**Figure 6:**
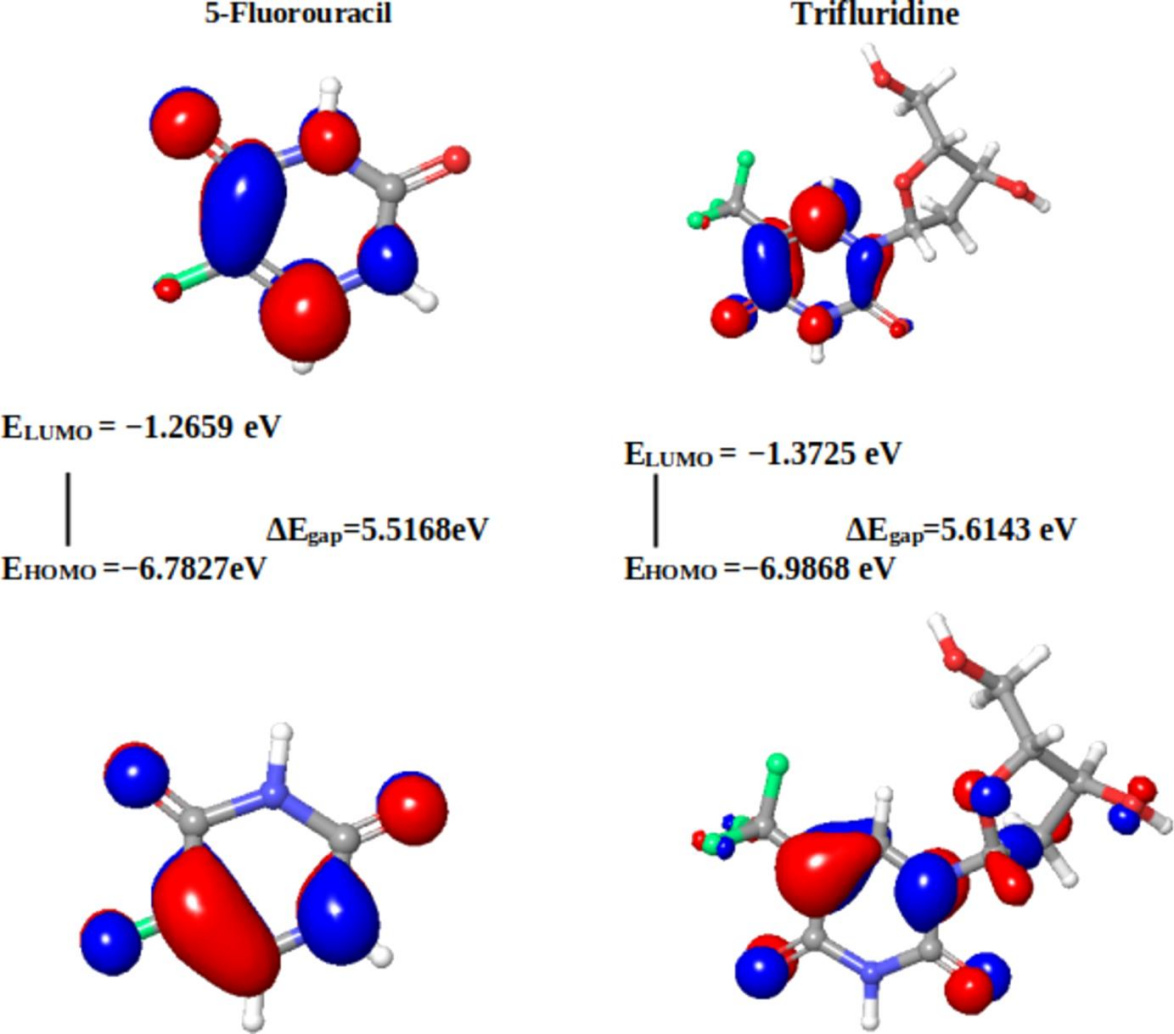
Frontier mol­ecular orbitals (FMOs) of 5-fluoro­uracil and trifluridine.

Within the structures of 5-fluoro­uracil and trifluridine (Fig. 6 ▸), the HOMO and LUMO exhibit localization in distinct regions of each respective mol­ecule. This localization directly signifies the occurrence of charge-transfer processes.

Turning our focus to broader implications, the eigenvalues of the HOMO and LUMO, along with their energy gap, offer crucial insights into the biological activity of a molecule (Table 3 ▸). A diminished energy gap, symbolized as Δ*E*_gap_, renders a mol­ecule more susceptible to polarization. This phenomenon aligns with heightened chemical reactivity and reduced kinetic stability, ultimately driving positive impetus toward biological activity. In contrast, an enlarged energy gap between the HOMO and LUMO orbitals signifies the kinetic instability of a mol­ecule, translating to a diminished propensity for biological activity (Pereira *et al.*, 2017 ▸; Akman, 2019 ▸; Choudhary *et al.*, 2013 ▸; Abdelsalam *et al.*, 2022 ▸). Adding a layer of nuance, **20** emerges as the mol­ecule showcasing the smallest charge separation (Δ*E*_gap_ = 3.5780 eV), suggesting its heightened potential for biological properties. In contrast, **17** displays the largest charge separation (Δ*E*_gap_ = 4.9242 eV), indicating a comparatively lower propensity for biological properties compared to the other synthesized Schiff bases.

In essence, the meticulous unravelling of these quantum features through DFT provides a profound understanding of the intricate inter­play between mol­ecular structure, reactivity and biological per­formance. Such insights hold transformative potential in advancing drug design and precision chemical manipulation. The distinctiveness of ligand **20** is further underscored by its characterization as the ‘softest’ mol­ecule (*S* = 0.5589 eV) and, consequently, the ‘least hard’ mol­ecule (η = 1.7890 eV). Conversely, **17** exhibits the highest hardness value (η = 2.4621 eV) and lowest softness (*S* = 0.4062 eV). When we turn our attention to standard drugs, the order of softness is observed as capecitabine > 5-fluoro­uracil > trifluridine, with all values generally lower than most synthesized Schiff bases. The chemical potential (μ) spans from −4.5354 eV (lowest) for **19** to −3.7908 eV (highest) for **17**. Schiff bases **19** and **20** exhibit the highest electrophilicity index (ω), with values of 5.044 and 4.9209 eV, respectively. In contrast, Schiff base **17** showcases the lowest electrophilicity index (ω = 2.9182 eV).

With regard to the computed global reactivity indices and the HOMO–LUMO gap, the order is: **20** > **19** > **21** > **18** > **23** > **22** > capecitabine > **17** > 5-fluoro­uracil > trifluridine. This insightful hierarchy provides valuable direction for the reactivity and potential biological activity of the synthesized Schiff bases.

#### Mol­ecular Electrostatic Potential (MESP)

The Mol­ecular Electrostatic Potential (MESP) concept serves as a window into the intricate charge distribution enveloping mol­ecules within the expanse of three-dimensional space. Its significance is particularly pronounced in identifying susceptible loci for electrophilic and nucleophilic inter­actions, which are critical in the realm of biological recognition and hydrogen-bonding phenomena. Through the utilization of colour mapping grounded in electron density, the electrostatic potential of the studied mol­ecules found visual expression, as illustrated in Figs. 2–6 ▸ ▸ ▸ ▸ ▸.

This visual representation employs a spectrum of colours to delineate the MESP surface characteristics. Red hues signify regions enriched with electrons, indicating a partially negative charge, while blue shades indicate electron-deficient zones with a partial positive charge. Light-blue nuances mark slightly electron-deficient areas, while yellow tinges highlight slightly electron-rich regions. Neutral zones with a zero potential are depicted in green (Altürk *et al.*, 2015 ▸; Friesner *et al.*, 2006 ▸).

**Figure 7:**
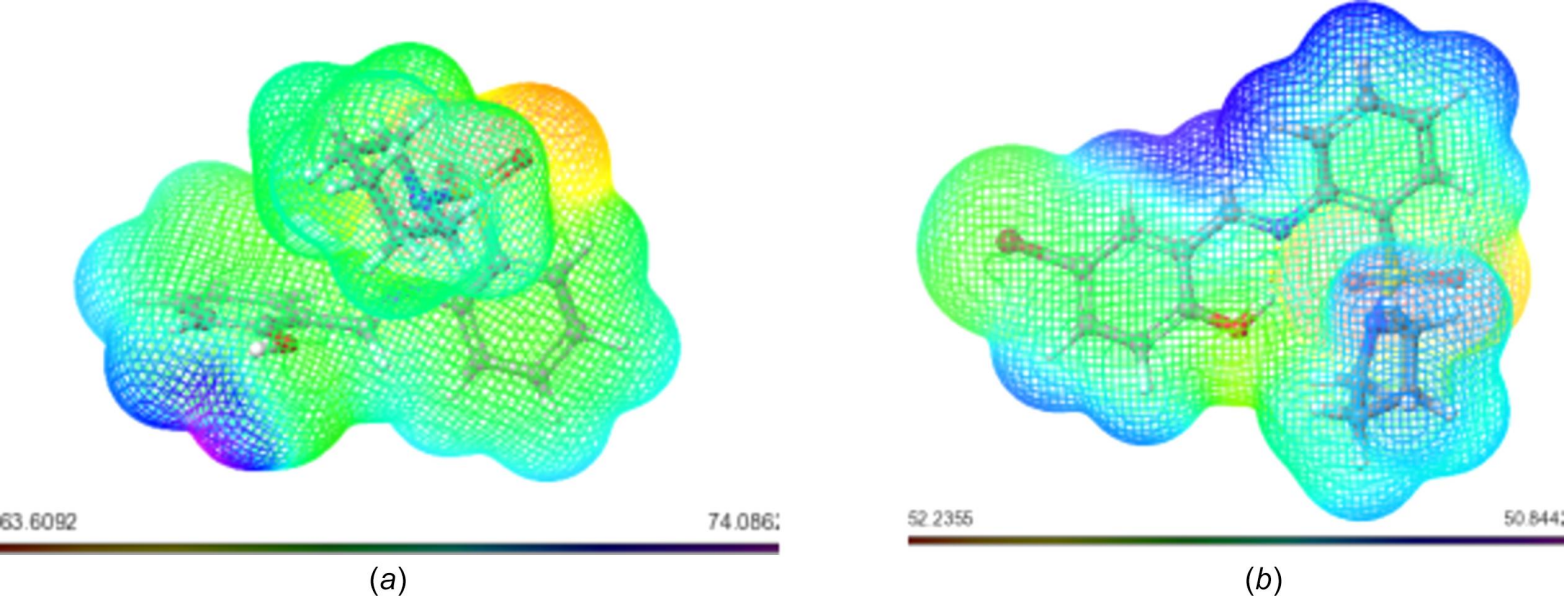
MESP plots of (*a*) **17** and (*b*) **20**. Regions of attractive potential appear in red and those of repulsive potential appear in blue.

**Figure 8:**
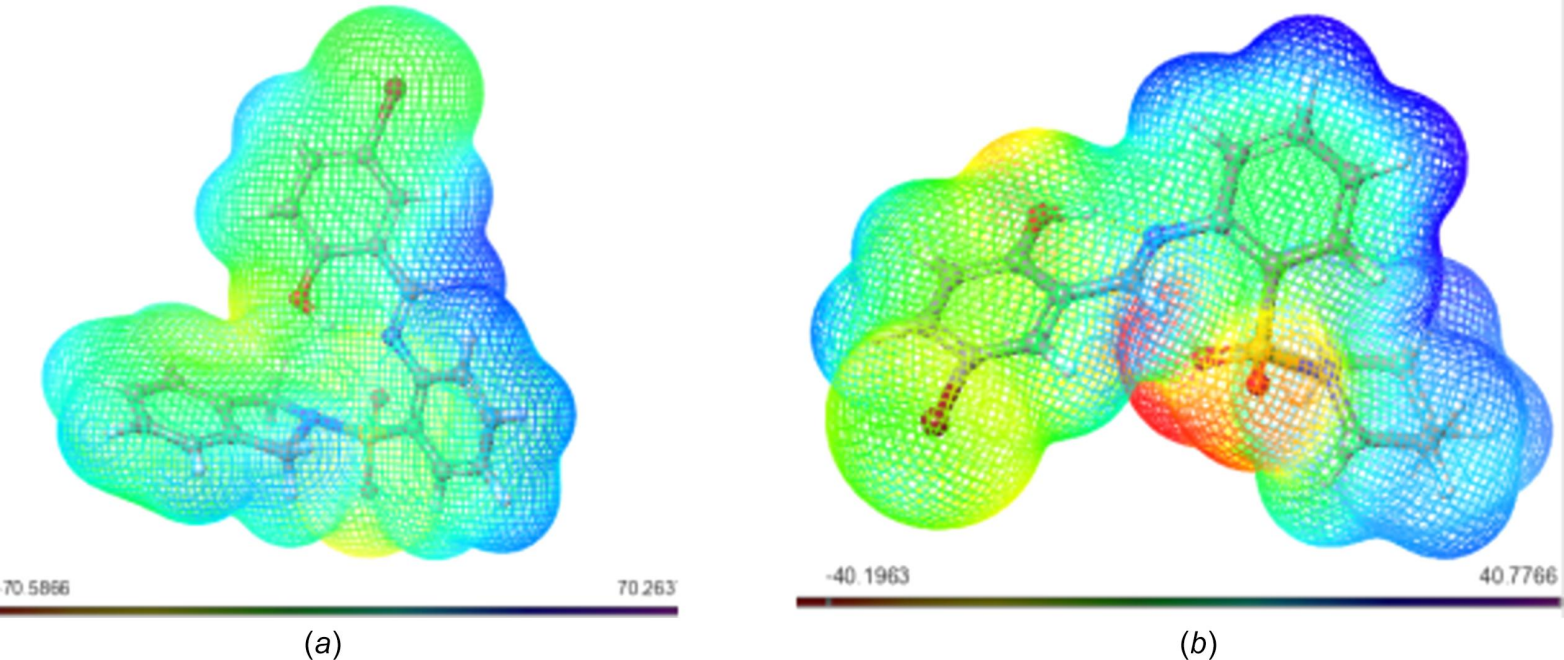
MESP plots of (*a*) **21** and (*b*) **18**.

**Figure 9:**
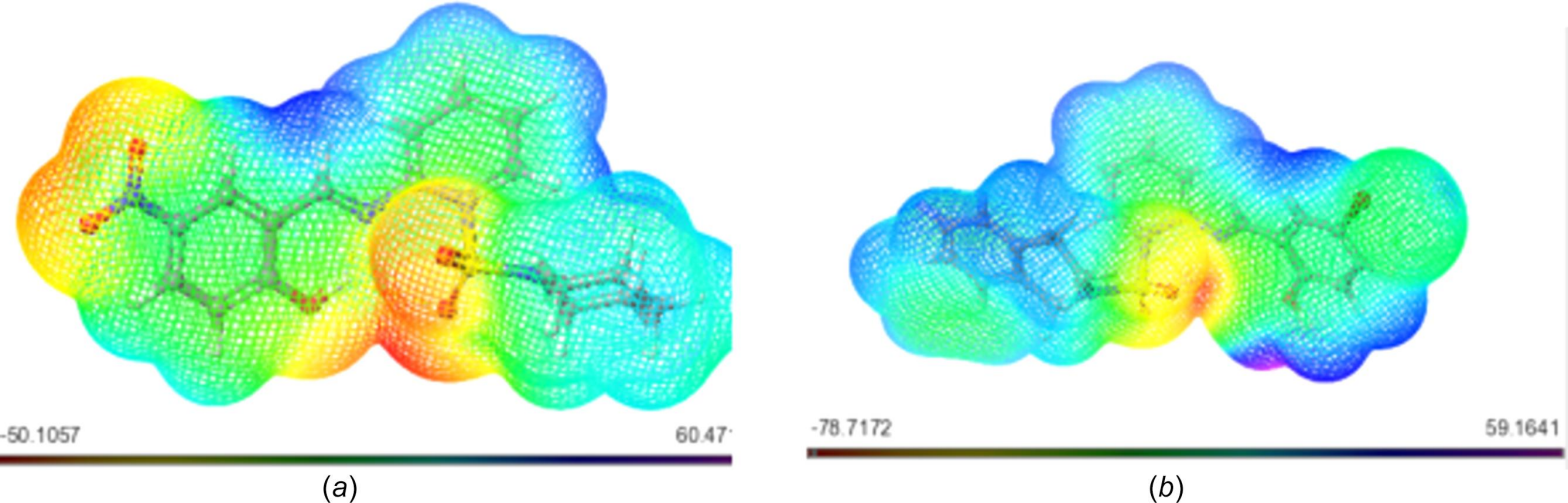
MESP plots of (*a*) **19** and (*b*) **22**.

**Figure 10:**
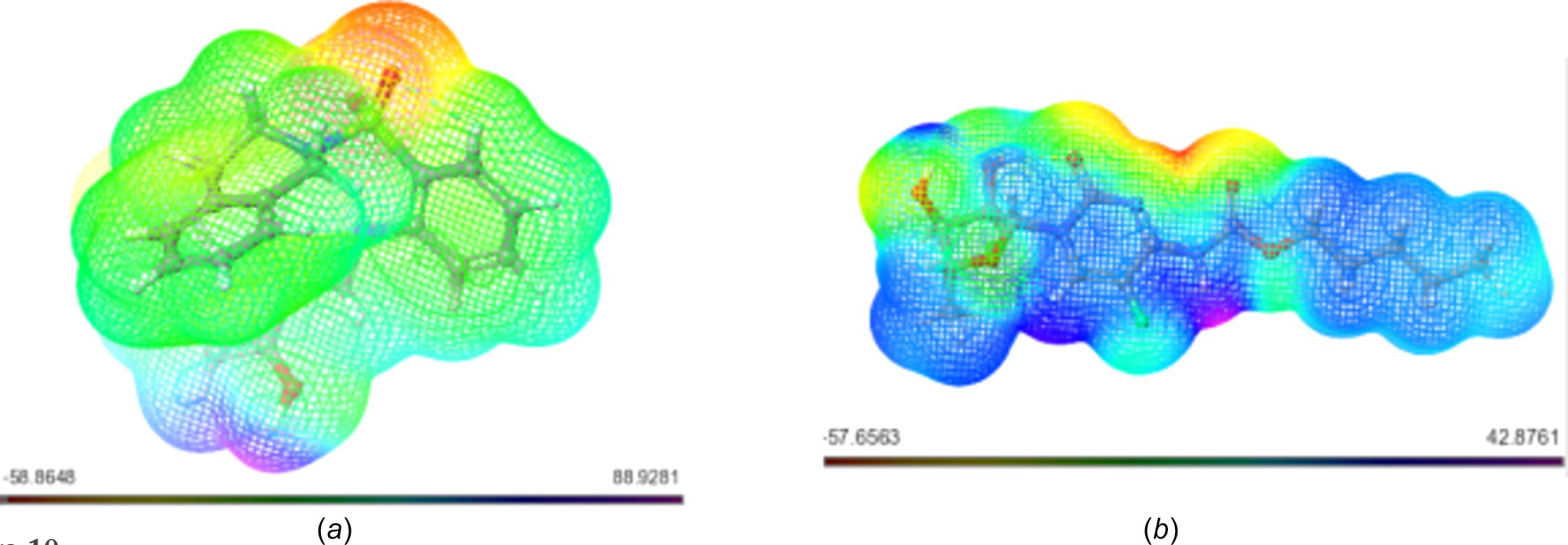
MESP plots of (*a*) **23** and (*b*) capecitabine.

**Figure 11:**
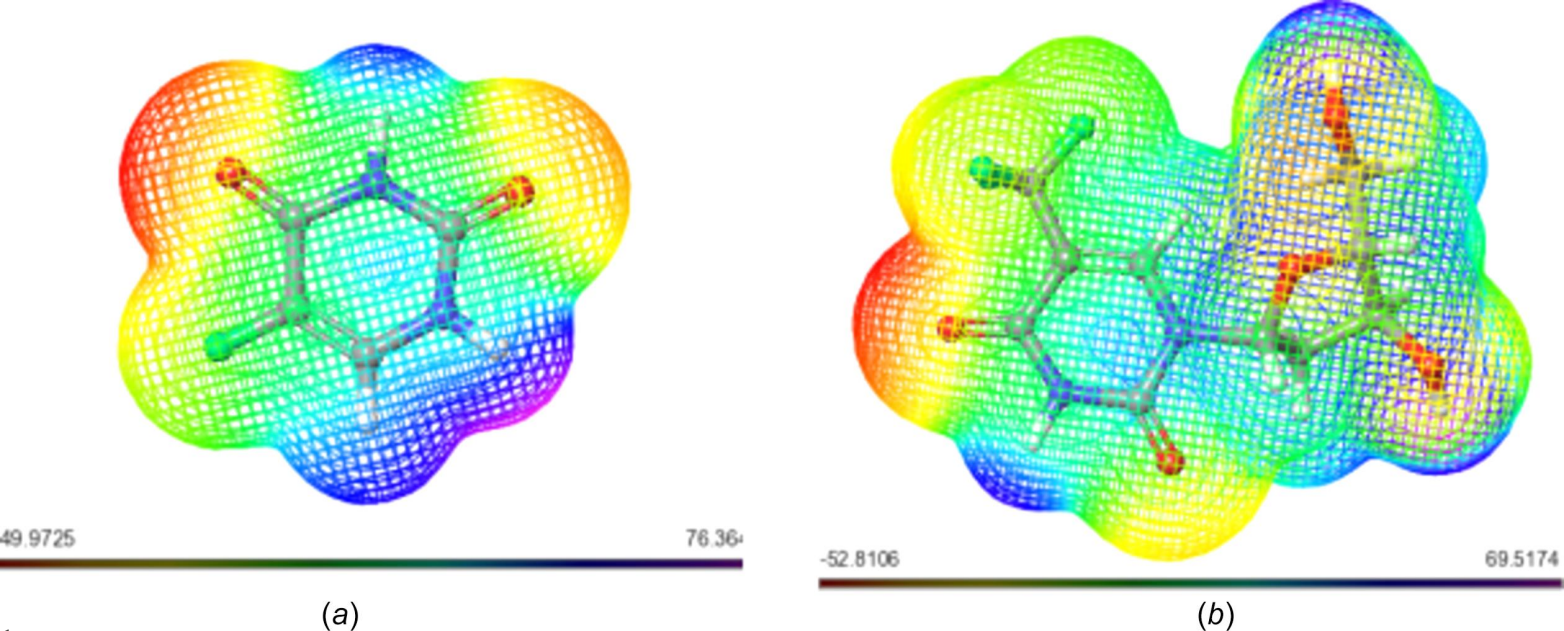
MESP plots of (*a*) 5-fluoro­uracil and (*b*) trifluridine.

Upon scrutinizing the MESP mappings of individual com­pounds, distinct patterns emerge. Schiff base **17** [Fig. 7 ▸(*a*)] pre­dominantly reveals a green surface, save for the hy­droxy-functionalized section which distinctly appears in blue. Similarly, the MESP profile of com­pound **20** [Fig. 7 ▸(*b*)] features prevalent blue regions, with a specific green–yellow region localized over the bromine-bearing arene ring. In the case of **21** [Fig. 8 ▸(*a*)], an evident gradient from green to blue characterizes the MESP map. Analogously, the MESP portrayal of **18** [Fig. 9 ▸(*b*)] reflects this trend, except for the S=O functional-group regions which assume a red hue. Both **19** [Fig. 9 ▸(*a*)] and **22** [Fig. 9 ▸(*b*)] display a blending of blue and green regions in their respective MESP renderings. For **23** [Fig. 10 ▸(*a*)], the MESP map predominantly features green hues, while the hy­droxy-enriched area adopts a distinctive blue shade. Noteworthy instances include the standard drug capecitabine [Fig. 10 ▸(*b*)], predominantly depicted in blue in its MESP representation. The standard drug 5-fluoro­uracil [Fig. 11 ▸(*a*)] showcases the entire spectrum of colour variations across its surface. Similarly, trifluridine [Fig. 11 ▸(*b*)] transitions from blue to red, thereby illustrating its surface characteristics encompassing the 2-(hy­droxy­meth­yl)tetra­hy­dro­furan-3-ol moi­ety.

### Docking studies

#### Schiff bases as potential inhibitors of tankyrase colon cancer protein mol­ecules

**Figure 12:**
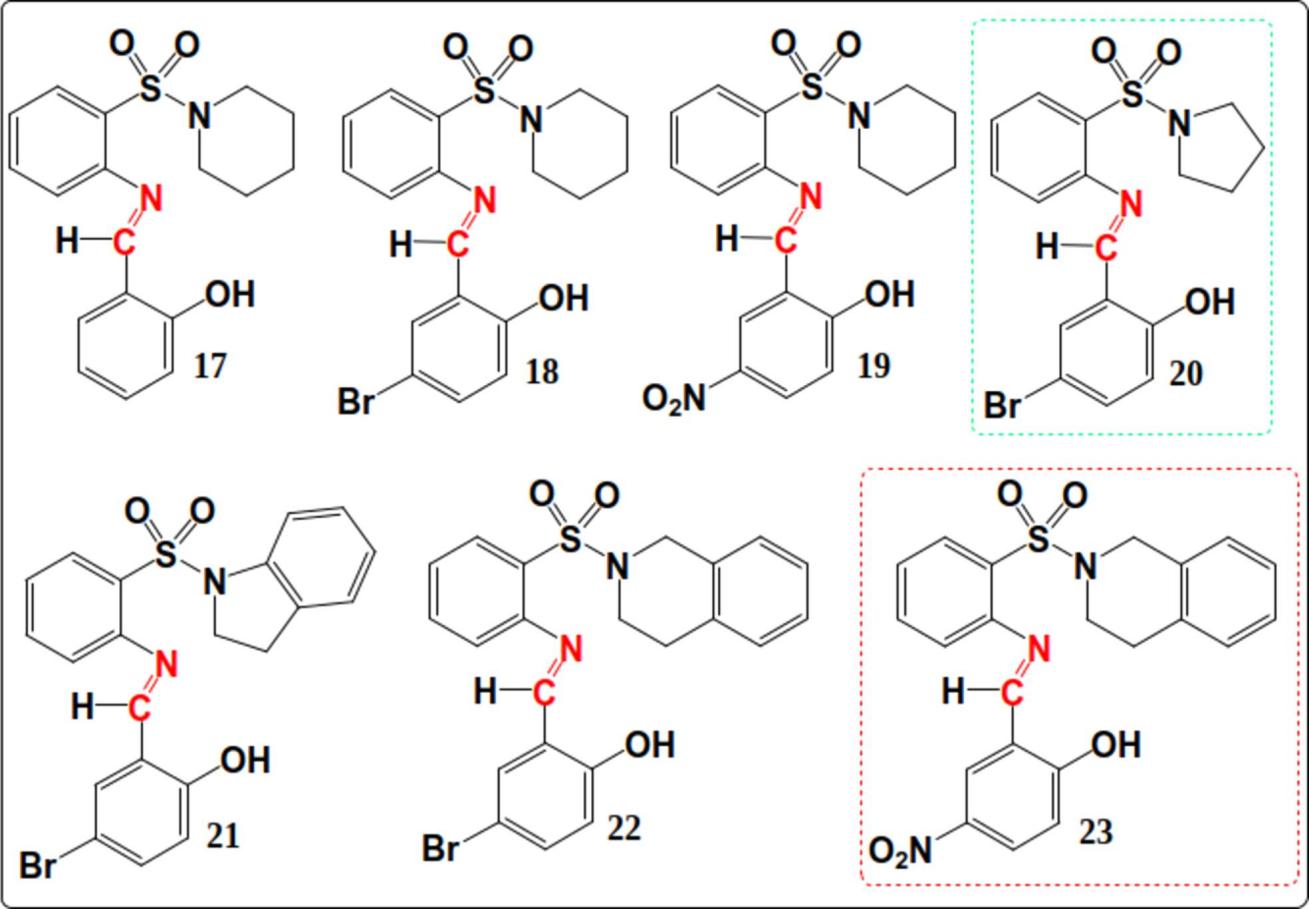
Structures of the synthesized Schiff bases **17**–**23** used for docking.

The docking car­ried out using *AutoDock Vina* on the PyRx website (https://pyrx.sourceforge.io/) and the summary of the binding energy of each ligand obtained is pre­sent­ed in Table 4 ▸ (the structures are pre­sent­ed in Fig. 12 ▸). In addition, the drug-likeness and toxicity of the synthesized Schiff bases and reference drugs were also investigated (Tables 5–10 ▸ ▸ ▸ ▸ ▸ ▸).

It is noteworthy that ligand **23** has the highest binding energy of −11.1 kcal mol^−1^, probably due to the presence of not only the sul­fon­amide but also the nitro group coupled with the tetra­hydro­iso­quinoline moiety binding to the protein.

The binding energy was observed to be significantly higher than that of the reference drugs trifluridine, capecitabine and 5-fluoro­uracil having binding energies of −8.0, −7.9 and −5.5 kcal mol^−1^, respectively. However, when the toxicity and the drug-likeness of ligand **23** were checked using the ProTox-II webserver and Swiss­ADME (http://www.swissadme.ch/), respectively, it was toxic and failed some of the rules despite its excellent binding inter­action.

Inter­estingly, ligands **17**, **20** and **18** were completely non­toxic and followed all drug-likeness rules, unlike all the other synthesized Schiff bases (*i.e.* **21**, **19**, **22** and **23**; see supporting information). In comparison, the reference drugs were also toxic, falling short of at least one drug-likeness rule. The best reference drug, *i.e.* trifluridine, and ligand **20**, namely, (*E*)-4-bromo-2-({[2-(pyrrolidin-1-ylsulfon­yl)phen­yl]imino}­meth­yl)phenol, with the best binding energy and in compliance with all drug-like rules and displaying complete nontoxicity, were selected for further study (Table 10 ▸).

**Figure 13:**
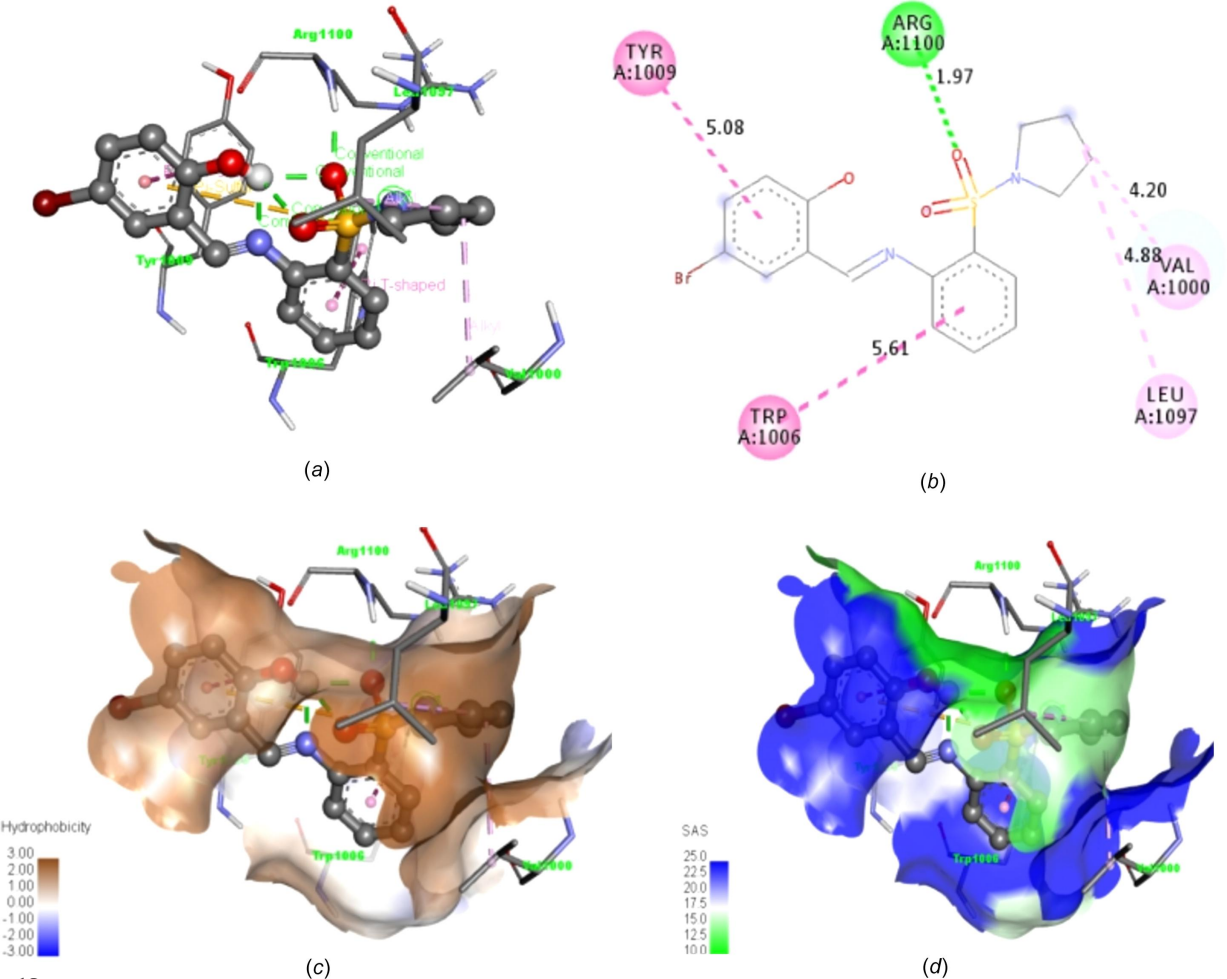
2D and 3D structures of synthesized Schiff base **20** showing (*a*) the inter­acting amino acid residues, (*b*) bond lengths, (*c*) hydro­phobic inter­actions and (*d*) solvent-accessibility surface.

The 2D and 3D structures of **20** showing the inter­acting amino acid residues [Fig. 13 ▸(*a*)], bond lengths [Fig. 13 ▸(*b*)], hydro­phobic inter­actions [Fig. 13 ▸(*c*)] and solvent-accessibility surface [Fig. 13 ▸(*d*)] are all pre­sent­ed. One of the sul­fon­amide O atoms exhibits a hydrogen-bonding inter­action with amino acid residue Arg_1100_ at a bonding distance of 1.97 Å, which is also noticeable within the atoms of the ligand in an intra­molecular fashion [Figs. 13 ▸(*a*) and 13(*b*)]. π-Alkyl and T-shaped inter­actions were also exhibited between the pyrrolidine moiety and amino acid residues Val_1000_ and Leu_1097_, and between the π-electrons of the two aromatic rings and the Trp_1006_ and Tyr_1009_ residues, respectively.

**Figure 14:**
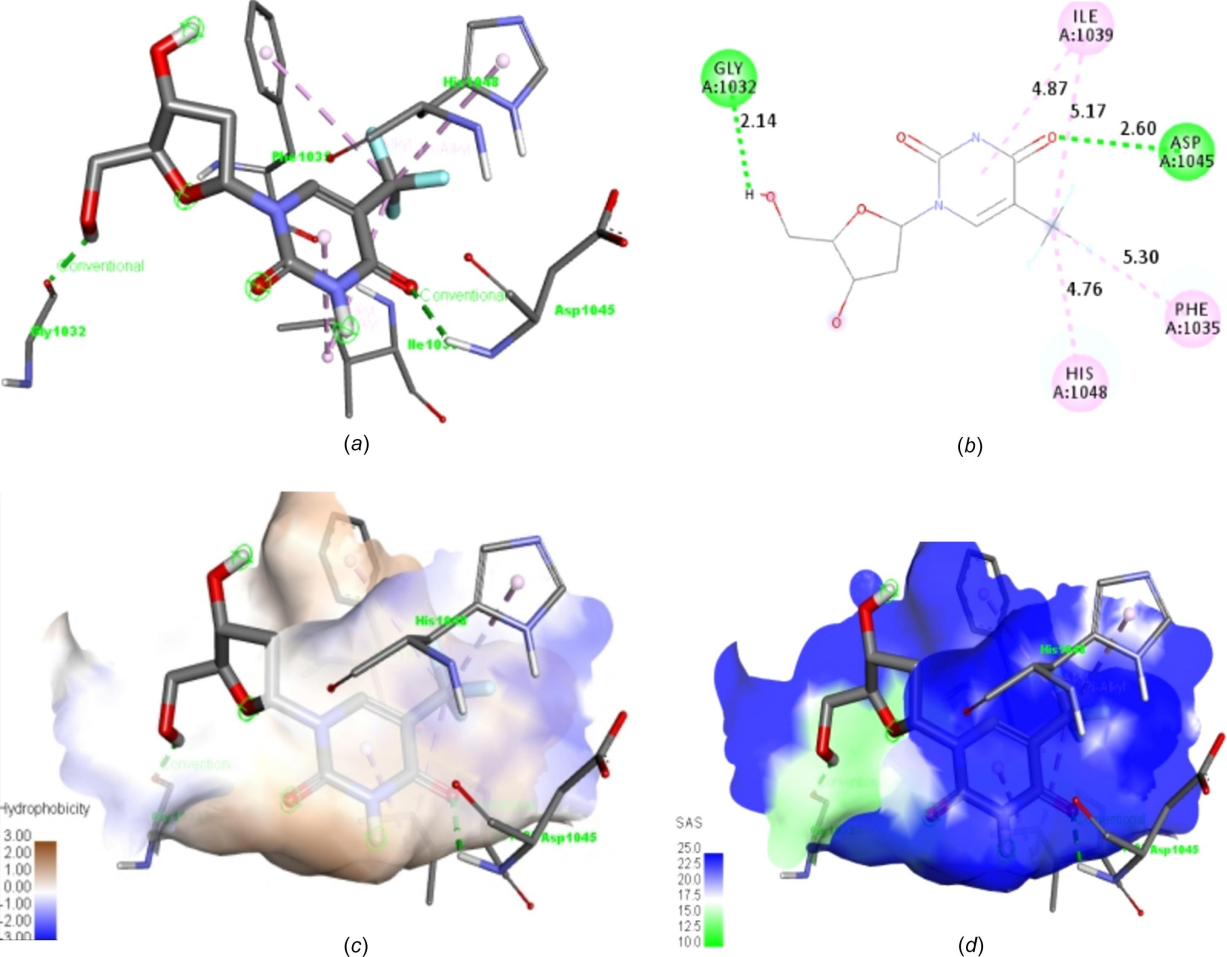
2D and 3D structures of the reference drug trifluridine, showing (*a*) the inter­acting amino acid residues, (*b*) bond lengths, (*c*) hydro­phobic inter­actions and (*d*) solvent-accessibility surface.

Significantly, the amino acid residues inter­acting with tankyrase poly(ADP-ribose) polymerase residues prefer hydro­phobic inter­actions [as depicted by the deep-brown region of Fig. 13 ▸(*c*)]. While Arg_1100_ had good solvent-accessibility surface inter­actions, other inter­acting residues had excellent solvent inter­actions with ligand **20** [Figs. 13 ▸(*c*) and 13(*d*)]. Although the reference drug trifluridine has two hydrogen-bond inter­actions, they are comparatively weaker and have longer bond lengths of 2.14 and 2.60 Å with Gly1032 and Asp1045, respectively, when compared with ligand **20** (1.90 Å), as pre­sent­ed in Figs. 14 ▸(*a*) and 14(*b*).

Noticeably, the solvent-accessibility surface of the reference drug seems better, as all inter­acting amino acid residues inter­act in the blue region [Fig. 14 ▸(*d*)]; however, it has comparably lower binding energy (Table 4 ▸), *i.e.* poorer hydro­phobicity than exhibited by most drug-like candidates [Fig. 14 ▸(*c*)], and it possesses mutagenic toxicity (Table 7 ▸).

#### Toxicity and drug-likeness of Schiff bases 17–23 and reference drugs

The SMILES (simplified mol­ecular-input line-entry system) of the synthesized Schiff bases and the reference drugs were obtained *via ChemDraw* (Version 14.0) software and *PubChem*, respectively. These SMILES were uploaded into the online webservers Pro-Tox-II and Swiss­ADME to investigate the *in-silico* toxicity and drug-likeness. A summary of the results obtained is pre­sent­ed in Tables 5–10 ▸ ▸ ▸ ▸ ▸ ▸. From the results, it is obvious that ligand **23** (−11.10 kJ mol^−1^) fell short of the toxicity test, despite being the best inter­acting ligand (Table 7 ▸). It could also not completely fit into the hexa­gonal drug-likeness physicochemical space. From the investigation, it became clear that ligand **20**, with a binding energy of −9.50 kJ mol^−1^, is completely nontoxic and fits perfectly into the hexa­gon, thereby displaying 100% drug-likeness (Table 6 ▸).

In comparison, the two common colon cancer reference drugs used in this study show some levels of toxicity. While trifluridine is mildly mutagenic, 5-fluoro­uracil is highly carcinogenic (Tables 8 ▸ and 9 ▸). Unlike synthesized Schiff bases **17**, **20** and **18**, this study also reveals that 5-fluoro­uracil fails some drug-likeness tests in addition to its toxic nature (Table 9 ▸).

In the course of the drug-likeness investigation, physicochemical parameters and drug-likeness violations of the Schiff bases and the reference drugs trifluridine and 5-fluoro­uracil were also compared, as shown in Tables 9 ▸ and 10 ▸. Most of the properties, such as the number of heavy atoms, rotatable bonds, TPSA (topological polar surface area), log *Kp* and bioavailability scores of the synthesized ligands compare effectively with trifluridine and 5-fluoro­uracil. Inter­estingly, none of the synthesized ligands violated Absorption, Distribution, Metabolism and Excretion (ADME) rules; hence, they can be tagged as potential drug candidates. While their gastrointestinal (GI) absorption is very high, the same properties exhibited by the reference drugs, none of the Schiff bases are blood–brain barrier permeant, making them safe without any unwarranted inter­ference with the central nervous system (Table 9 ▸).

## Conclusion

The successful synthesis, characterization and analysis of the inter­molecular inter­actions of *N* ^1^-(5^1^-substituted-2^1^-hy­droxy­benzyl­iden­yl)-*N*-cyclo­amino-2-sulf­anil­amides (com­pounds **17**–**23**) have been achieved, alongside their evaluation for inhibitory effects on tankyrase poly(ADP-ribose) polymerase in the context of colon cancer through *in-silico* testing. Crystal packing and density functional theory (DFT) analyses have indicated that hydrogen bonds and π–π stacking play a crucial role in the mol­ecular cohesion of these com­pounds. Furthermore, the DFT results, when combined with mol­ecular docking studies, reveal that the electronegativity and electrophilicity attributes of these com­pounds significantly influence their binding affinity towards tankyrase poly(ADP-ribose) polymerase. This comprehensive study not only sheds light on the underlying mechanisms of action but also lays down a foundational framework for the development of effective therapies against colon cancer based on com­pounds **17**–**23**. 

## Supplementary Material

Crystal structure: contains datablock(s) I, global. DOI: 10.1107/S205322962400233X/ef3052sup1.cif

Structure factors: contains datablock(s) I. DOI: 10.1107/S205322962400233X/ef3052Isup2.hkl

Supporting information file. DOI: 10.1107/S205322962400233X/ef3052sup3.pdf

CCDC reference: 2305610

## Figures and Tables

**Table 1:** Experimental details for **18**

Crystal data	
Chemical formula	C_18_H_19_BrN_2_O_3_S
*M*_r_	423.32
Crystal system, space group	Orthorhombic, *P* *b* *c* *a*
Temperature (K)	296
*a*, *b*, *c* (Å)	12.4070 (9), 17.4250 (14), 17.5276 (12)
*V* (Å^3^)	3789.3 (5)
*Z*	8
Radiation type	Mo *K*α
μ (mm^−1^)	2.30
Crystal size (mm)	0.84 × 0.43 × 0.12

Data collection	
Diffractometer	Bruker APEXII CCD
Absorption correction	Multi-scan (*SADABS*; Bruker, 2016 ▸)
*T*_min_, *T*_max_	0.133, 0.241
No. of measured, independent and observed [*I* > 2σ(*I*)] reflections	27860, 3356, 2255
*R*_int_	0.060
(sin θ/λ)_max_ (Å^−1^)	0.597

Refinement	
*R*[*F* ^2^ > 2σ(*F* ^2^)], *wR*(*F* ^2^), *S*	0.062, 0.172, 1.09
No. of reflections	3356
No. of parameters	227
H-atom treatment	H-atom parameters constrained
Δρ_max_, Δρ_min_ (e Å^−3^)	0.52, −0.38

Computer programs: *APEX2* (Bruker, 2016 ▸), *SAINT* (Bruker, 2016 ▸), *SHELXT2018* (Sheldrick, 2015*a* ▸), *SHELXL2018* (Sheldrick, 2015*b* ▸), *ShelXle* (Hübschle *et al.*, 2011 ▸), *ORTEP-3 for Windows* (Farrugia, 2012 ▸), *PLATON* (Spek, 2020 ▸) and *Mercury* (Macrae *et al.*, 2020 ▸).

**Table 2:** Hydrogen-bond geometry for **18** (Å, °) *Cg* is the centroid of the C11–C16 ring.

*D*—H⋯*A*	*D*—H	H⋯*A*	*D*⋯*A*	*D*—H⋯*A*
O1—H1*A*⋯N1	0.82	1.86	2.586 (5)	146
C1—H1⋯O2^i^	0.93	2.54	3.363 (6)	149
C16—H16⋯O2^i^	0.93	2.64	3.461 (6)	147
C23—H23⋯O3	0.93	2.43	2.846 (7)	107
C25—H25⋯O3^ii^	0.93	2.49	3.220 (6)	135
C26—H26⋯O1^i^	0.93	2.62	3.467 (7)	151
C35—H35*B*⋯O2	0.97	2.43	2.852 (7)	106
C32—H32*B*⋯*Cg* ^iii^	0.97	2.88	3.664 (7)	139

Symmetry codes: (i) 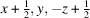 ; (ii) 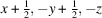 ; (iii) 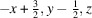 .

**Table 3:** Global reactivity descriptors of the synthesized Schiff bases and standard drugs

Entry	*E*_HOMO_	*E*_LUMO_	Δ*E*_gap_	*I*	*A*	μ	*X*	η	*S*	ω
**17**	−6.2529	−1.3287	4.9242	6.2529	1.3287	−3.7908	3.7908	2.4621	0.4062	2.9183
**20**	−5.9851	−2.4071	3.5780	5.9851	2.4071	−4.1961	4.1961	1.7890	0.5590	4.9210
**21**	−6.1535	−2.0050	4.1485	6.1535	2.0050	−4.0793	4.0793	2.0743	0.4821	4.0112
**18**	−6.1546	−2.0050	4.1496	6.1546	2.0050	−4.0798	4.0798	2.0748	0.4820	4.0112
**19**	−6.5745	−2.4963	4.0782	6.5745	2.4963	−4.5354	4.5354	2.0391	0.4904	5.0439
**22**	−6.1778	−1.6035	4.5743	6.1778	1.6035	−3.8907	3.8907	2.2872	0.4372	3.3092
**23**	−6.6167	−2.2713	4.3454	6.6167	2.2713	−4.4440	4.4440	2.1727	0.4603	4.5448
5-Flu	−6.7827	−1.2659	5.5168	6.7827	1.2659	−4.0243	4.0243	2.7584	0.3625	2.9355
Cap	−6.4578	−1.6019	4.8559	6.4578	1.6019	−4.0299	4.0299	2.4279	0.4119	3.3444
Tri	−6.9868	−1.3725	5.6143	6.9868	1.3725	−4.1797	4.1797	2.8071	0.3562	3.1117

Notes: Δ*E*_gap_ is the energy gap or charge separation, *I* is ionization potential, *A* is electron affinity, μ is chemical potential, *X* is electronegativity, η is global hardness, *S* is global softness and ω is the electrophilicity index. Tri is trifluridine, Cap is capecitabine and 5-Flu is 5-fluoro­uracil.

**Table 4:** Summary of the binding energy (kcal mol^−1^) of Schiff bases with poly(ADP-ribose) polymerase

Optimized Schiff bases	Summarized drug-likeness and toxicity	6kro
6kro_23_E=714.59	mildly nondrug-like and toxic	−11.1
6kro_22_E=797.81	nontoxic but mildly nondrug-like	−10.3
6kro_21_E=687.32	nondrug-like and nontoxic	−9.9
6kro_20_E=635.91	drug-like and nontoxic	−9.5
6kro_17_E=666.05	drug-like and nontoxic	−9.2
6kro_18_E=685.47	drug-like and nontoxic	−8.7
6kro_19_E=748.77	drug-like but mildly toxic	−6.7
6kro_trifluridine_E=282.80	drug-like but mildly toxic	−8
6kro_capecitabine_E=624.15	drug-like but highly toxic	−7.9
6kro_5-fluoro­uracil_E=45.84	mildly nondrug-like and toxic	−5.5

**Table 7:** *In-silico* toxicity study and drug-likeness of trifluridine

**Druglikeness**		
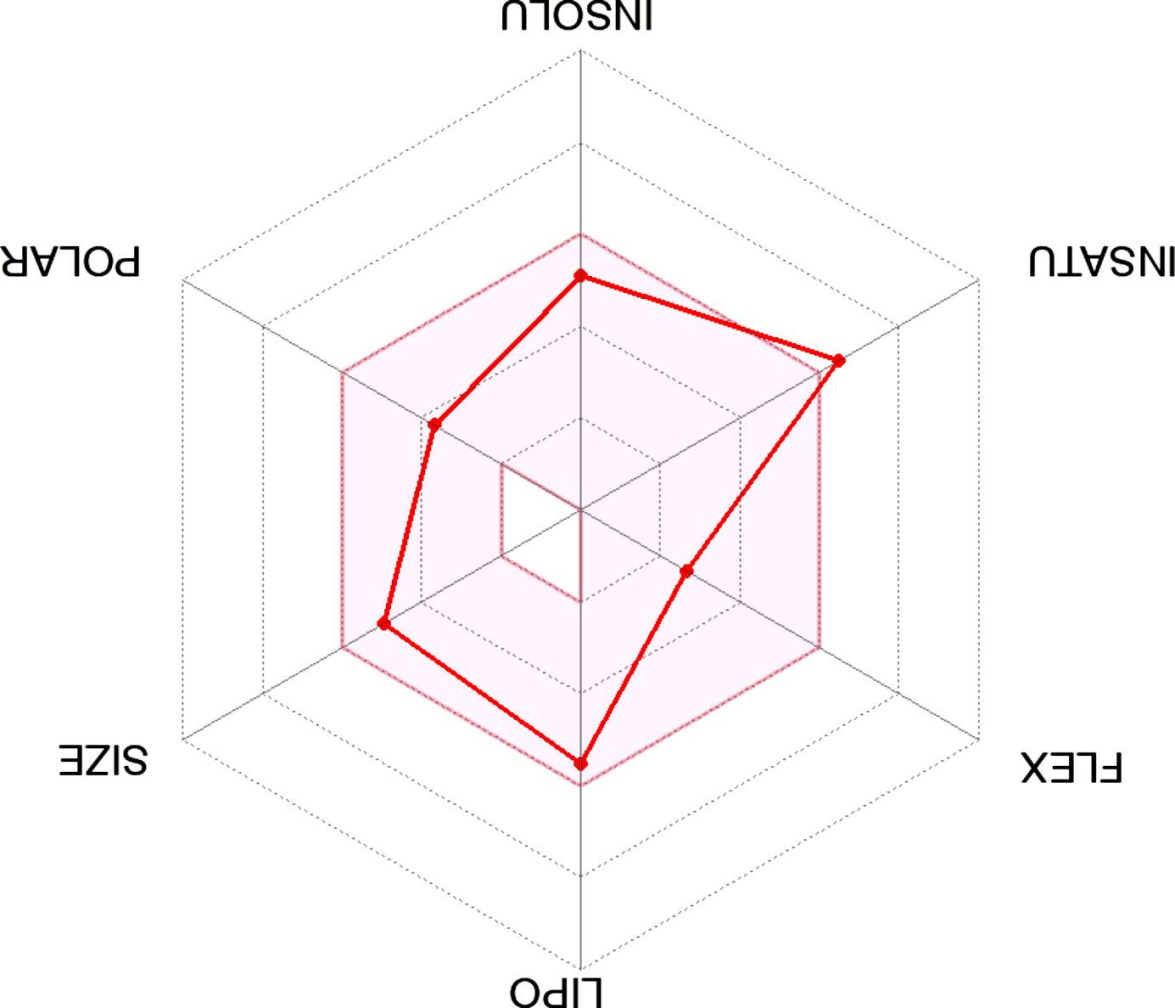		
**Toxicity**		
Target	Prediction	Probability
Hepatotoxicity	Inactive	0.76
Carcinogenicity	Inactive	0.60
Immunotoxicity	Inactive	0.99
Mutagenicity	Active	0.64
Cytotoxicity	Inactive	0.88

**Table 6:** *In-silico* toxicity study and drug-likeness of **23** using ProTox-II and Swiss­ADME

**Druglikeness**		
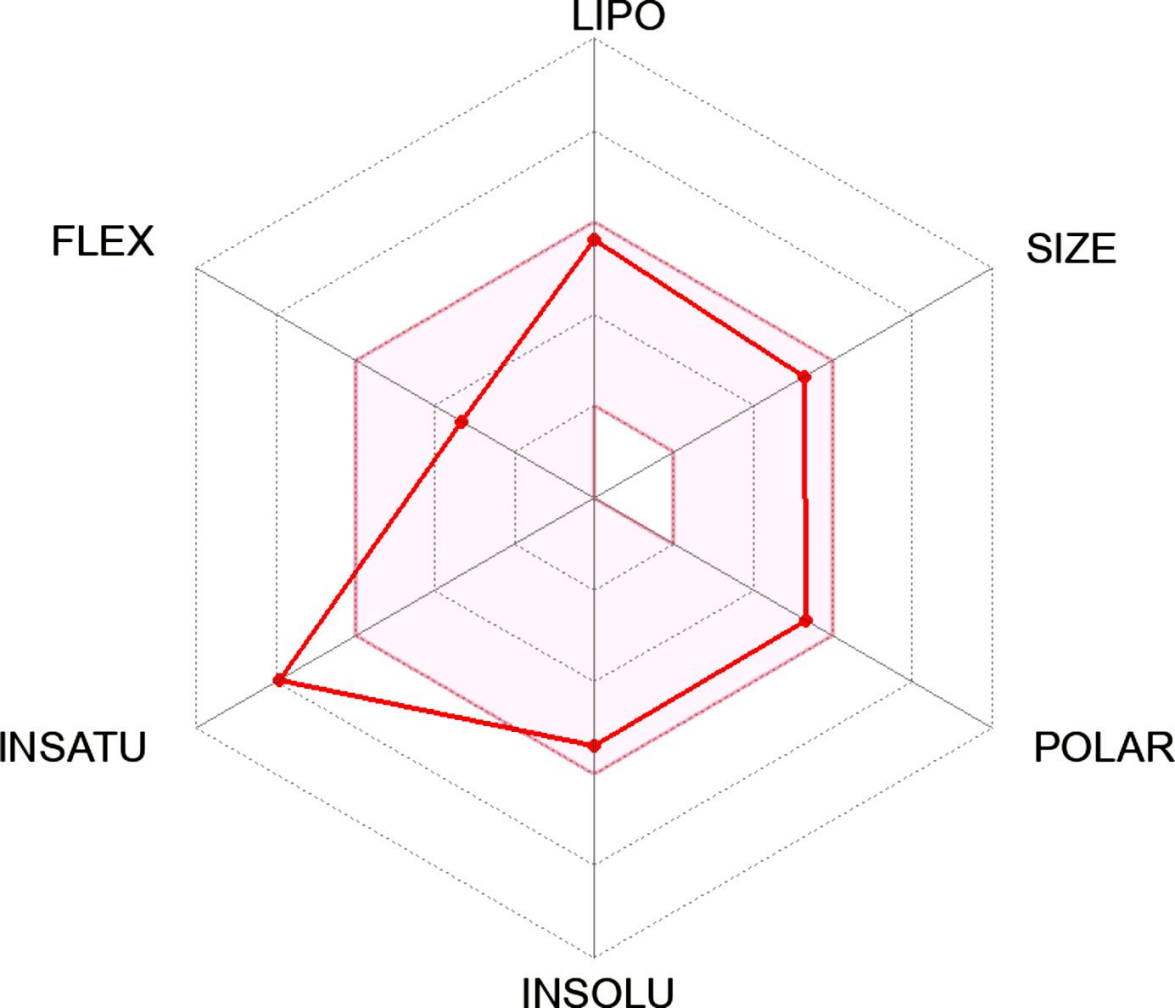		
**Toxicity**		
Target	Prediction	Probability
Hepatotoxicity	Inactive	0.62
Carcinogenicity	Inactive	0.57
Immunotoxicity	Inactive	0.94
Mutagenicity	Active	0.68
Cytotoxicity	Inactive	0.78

**Table 5:** *In-silico* toxicity study and drug-likeness of **20** using ProTox-II and Swiss­ADME

**Druglikeness**		
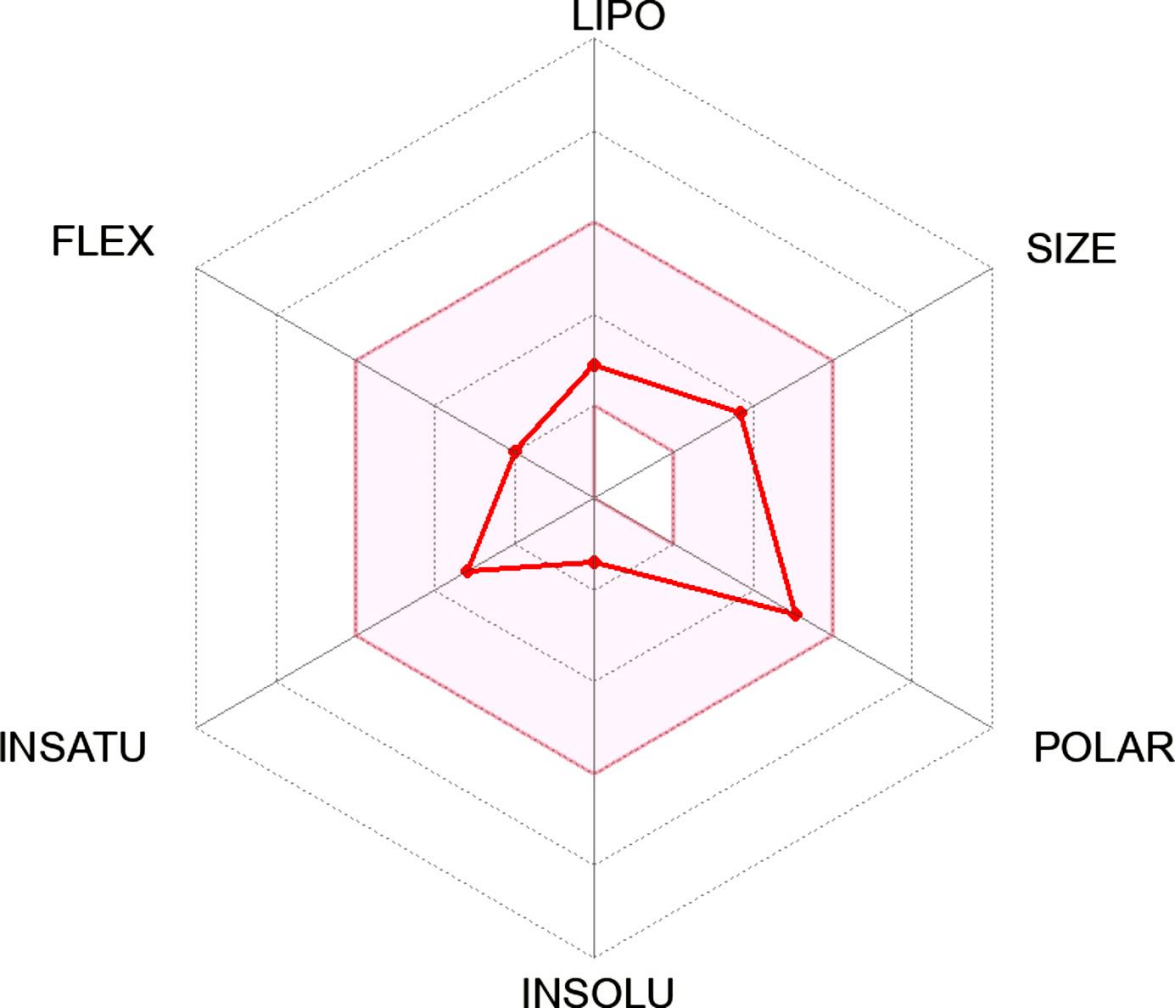		
**Toxicity**		
Target	Prediction	Probability
Hepatotoxicity	Inactive	0.57
Carcinogenicity	Inactive	0.57
Immunotoxicity	Inactive	0.87
Mutagenicity	Inactive	0.71
Cytotoxicity	Inactive	0.73

**Table 8:** *In-silico* toxicity study and drug-likeness of 5-fluoro­uracil

**Druglikeness**		
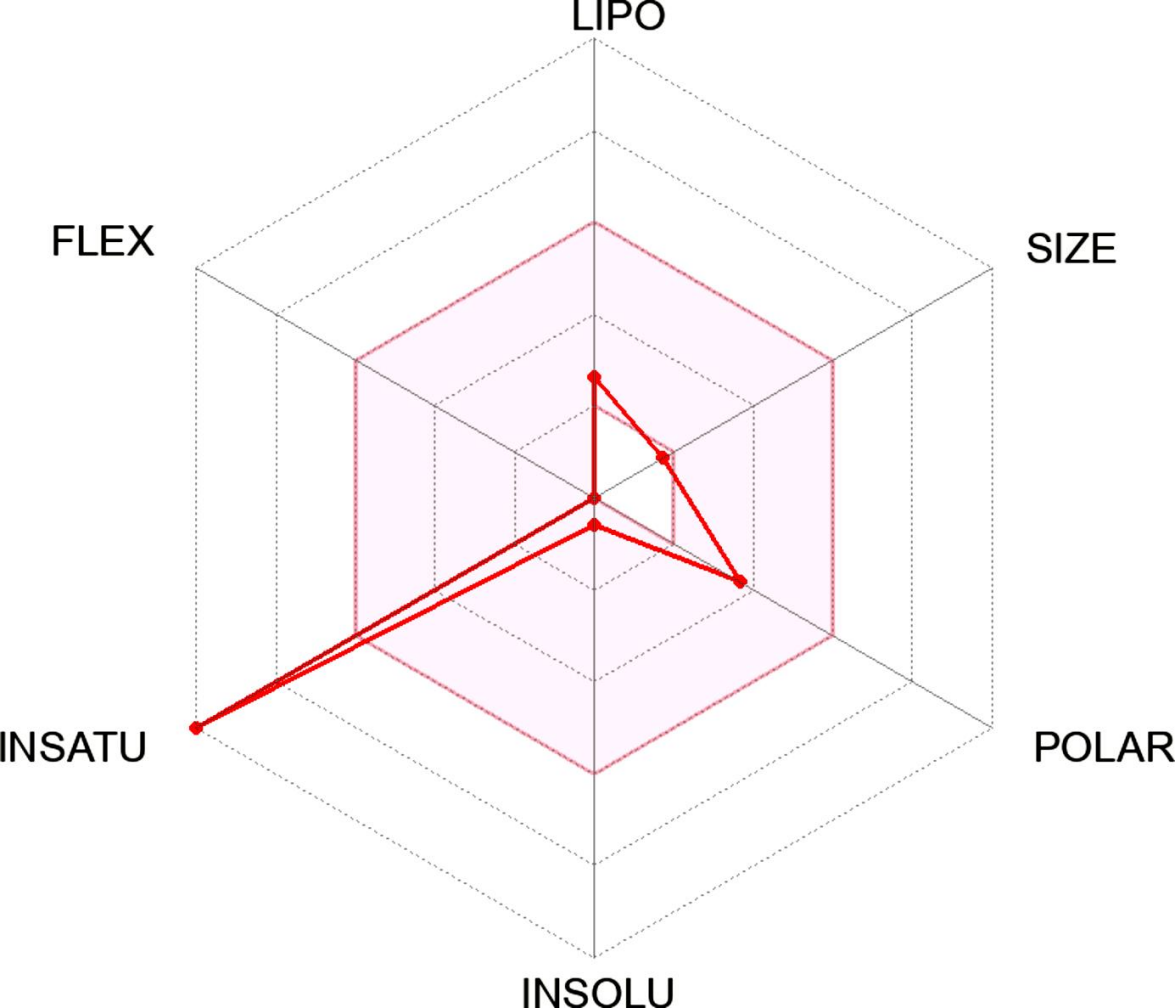		
**Toxicity**		
Target	Prediction	Probability
Hepatotoxicity	Inactive	0.78
Carcinogenicity	Active	0.85
Immunotoxicity	Inactive	0.99
Mutagenicity	Inactive	0.88
Cytotoxicity	Inactive	0.93

**Table 9:** Physicochemical properties of the synthesized Schiff bases and reference drugs

Compound	*M*_r_	No. of heavy atoms	Fraction C*sp* ^3^	No. rotational bonds	No. hydrogen-bond acceptors	No. hydrogen-bond donors	TPSA	log *Kp* (cm s^−1^)	Bioavailability score
**17**	344.43	24	0.28	4	5	1	78.35	−6.37	0.55
**20**	409.3	24	0.24	4	5	1	78.35	−6.53	0.55
**21**	457.34	28	0.10	4	4	1	78.35	−6.04	0.55
**18**	423.32	25	0.28	4	5	1	78.35	−6.36	0.55
**19**	389.43	27	0.28	5	7	1	124.17	−6.77	0.55
**22**	471.37	29	0.14	4	5	1	78.35	−6.17	0.55
**23**	437.47	31	0.14	5	7	1	124.17	−6.58	0.55
Trifluridine	296.2	20	0.60	3	8	3	104.55	−8.43	0.55
Capecitabine	359.35	25	0.67	8	8	3	122.91	−8.09	0.55
5-Fluoro­uracil	130.08	9	0	0	3	2	65.72	−7.73	0.55

**Table 10:** Drug-likeness rule violation and *in-silico* toxicity study of trifluridine and 5-fluoro­uracil for comparison GI is gastrointestinal and BBB is blood–brain barrier.

Drug-likeness rules violations						Blood–brain distribution and metabolism	
Compounds	Lipinski	Ghose	Veber	Egan	Muegge	GI absorption	BBB permeant
**17**	0	0	0	0	0	High	No
**20**	0	0	0	0	0	High	No
**21**	0	0	0	0	0	High	No
**18**	0	0	0	0	0	High	No
**19**	0	0	0	0	0	High	No
**22**	0	0	0	0	0	Low	No
**23**	0	0	0	0	0	High	No
Trifluridine	0	0	0	0	0	High	No
Capecitabine	0	0	0	0	0	High	No
5-Fluoro­uracil	0	0	0	0	0	High	No
