# Supplementary material for: Synthesis, crystal structure and in-silico evaluation of aryl­sul­fon­amide Schiff bases for potential activity against colon cancer
Source: Acta Crystallogr C Struct Chem. 2024 Mar 28;80(Pt 4):129–42. doi: 10.1107/S205322962400233X (PMC10996187; doi:10.1107/S205322962400233X)
Supplement: Supplementary file 3 [file c-80-00129-sup3.pdf]

Acta Cryst C STRUCTURAL CHEMISTRY

Volume 80 (2024)

Supporting information for article:

Synthesis, crystal structure and *in-silico* evaluation of arylsulfonamide Schiff bases for potential activity against colon cancer

Sherif O. Kolade, Oluwafemi S. Aina, Allen T. Gordon, Eric C. Hosten, Idris A. Olasupo, Adeniyi S. Ogunlaja, Olayinka T. Asekun and Oluwole B. Familoni

2.2.1.1. Method A: Synthesis of N^1-(2[1]-hydroxybenzylidenyl)-*N*-piperidinyl-2-sulphanilamide **17**

Orange crystals (0.118g, 82%); R_f = 0.41 (2:1 $CHCl_3$/*n*-hexane); 153-155 °C IR (Bruker, ATR, *v* cm^{-1}): 3101 (C-O-H str.), 3084 (*aryl* C–H str.), 2943 (*sp^3*-C–H str.), 1616 (C=N str.), 1566 (*aryl* C=C str.), 1300 (*asym* SO_2–N str.), 1148 (*sym* SO_2–N str.); ^{1}H-NMR (Bruker, 400 MHz, $CDCl_3$, δ_H, ppm): 12.43 (1H, s, O**H**), 8.48 (1H, s, **H**C=N), 7.93 (1H, d, *J* = 8 Hz, Ar**H**), 7.54 (1H, t, *J* = 8 Hz, Ar**H**), 7.34 (2H, t, *J* = 6 Hz, Ar**H**), 7.29 (1H, d, *J* = 8 Hz, Ar**H**), 7.12 (1H, d, *J* = 8 Hz, Ar**H**), 6.98 (1H, d, *J* = 8 Hz, Ar**H**), 6.88 (1H, t, *J* = 8 Hz, Ar**H**), 3.04 (4H, t, *J* = 4 Hz, -C**H**$_2$NC**H**$_2$-), 1.50–1.45 (4H, m, -C**H**$_2$CH$_2$C**H**$_2$-), 1.37–1.36 (4H, m, -CH$_2$C**H**$_2$CH$_2$-); ^{13}C-NMR (Bruker, 100 MHz, $CDCl_3$, δ_C, ppm): 164.2 (**Ar**-OH), 161.1 (H**C**=N), 147.6 (**Ar**-N), 134.1; 134.0; 132.8; 131.3; 131.2; 126.2; 120.5; 119.1; 119.0; 117.7 (**Ar**H), 46.3 (-**C**H$_2$N**C**H$_2$-), 25.4 (-**C**H$_2$CH$_2$**C**H$_2$-), 23.7 (-CH$_2$**C**H$_2$CH$_2$-); (GC–MS) *m/z*: calculated for $[C_{18}H_{20}N_2O_3S + H]^+$ 344.1, found 344.1.

2.2.1.2. Method B: Synthesis of N^1-(5[1]-bromo/nitro-2[1]-hydroxybenzylidenyl)-*N*-cycloamino-2-sulphanilamides **18-23**

2.2.1.2.1. N^1-(5[1]-bromo-2[1]-hydroxybenzylidenyl)-*N*-piperidinyl-2-sulfanilamide 18

N-Piperidinyl-*o*-sulphanilamide 11 (0.100 g, 0.417 mmol) and 5-bromo-2-salicylaldehyde 15 (0.109 g, 0.542 mmol). Yellow crystals (0.132 g, 75%); R_f = 0.28 (1.1:1 $CHCl_3$/*n*-hexane); m.p. 154.2–154.4 °C; IR (Bruker, ATR, *v* cm^{-1}): 3000 (*aryl* C–H str.), 2948 (*sp^3*-C–H str.), 1616 (C=N str.), 1559 (*aryl* C=C str.), 1318 (*asym* SO_2–N str.), 1148 (*sym* SO_2–N str.), 606 (C–Br str.); ^{1}H-NMR (Bruker, 400 MHz, $CDCl_3$, δ_H, ppm): 12.46 (1H, s, O**H**), 8.41 (1H, s, **H**C=N), 7.93 (1H, d, *J* = 8 Hz, Ar**H**), 7.56 (1H, t, *J* = 8 Hz, Ar**H**), 7.45 (1H, s, Ar**H**), 7.41 (1H, d, *J* = 8 Hz, Ar**H**), 7.33 (1H, t, *J* = 8 Hz, Ar**H**), 7.11 (1H, d, *J* = 8 Hz, Ar**H**), 6.89 (1H, d, *J* = 12 Hz, Ar**H**), 3.02 (4H, t, *J*

= 4 Hz, -C**H**$_2$NC**H**$_2$-), 1.52–1.38 (6H, m, -C**H**$_2$C**H**$_2$C**H**$_2$); ^{13}C-NMR (Bruker, 100 MHz, $CDCl_3$, δ_C, ppm): 162.7 (**Ar**-OH), 160.1 (H**C**=N), 147.0 (**Ar**-N), 136.6; 135.7; 134.7; 134.1; 131.5; 131.2; 126.7; 120.3; 119.8; 110.6 (**Ar**H), 46.3 (-**C**H$_2$N**C**H$_2$-), 25.4 (-**C**H$_2$CH$_2$**C**H$_2$-), 23.7 (-CH$_2$**C**H$_2$CH$_2$-); (GC–MS) *m/z*: calculated for $[C_{18}H_{19}B_rN_2O_3S + H]^+$ 422.0, found 422.0.

2.2.1.2.2. *N^1*-(2^1-hydroxy-5^1-nitrobenzylidenyl)-*N*-piperidinyl-2-sulfanilamide **19**

N-Piperidinyl-*o*-sulphanilamide 11 (0.100 g, 0.417 mmol) and 5-nitro-2-salicylaldehyde 16 (0.091 g, 0.542 mmol). Yellow crystals (0.130 g, 80%); R_f 0.35 (1.1:1 $CHCl_3$/*n*-hexane); m.p. 159.2–159.4 ºC; IR (Bruker, ATR, *v* cm^{-1}): 3092 (*aryl* C–H str.), 2942 (*sp^3*-C–H str.), 1617 (C=N str.), 1563 (*aryl* C=C str.), 1330 (*asym* SO_2–N str.), 1118 (*sym* SO_2–N str.), 1512 (*asym* NO_2 str.), 1328 (*sym* NO_2 str.); ^{1}H-NMR (Bruker, 400 MHz, $CDCl_3$, δ_H, ppm): 13.50 (1H, s, O**H**), 8.67 (1H, s, **H**C=N), 8.44 (1H, s, Ar**H**), 8.32 (1H, d, *J* = 8 Hz, Ar**H**), 8.03 (1H, d, *J* = 8 Hz, Ar**H**), 7.70 (1H, t, *J* = 8 Hz, Ar**H**), 7.48 (1H, t, *J* = 8 Hz, Ar**H**), 7.28 (1H, d, *J* = 4 Hz, Ar**H**), 7.17 (1H, d, *J* = 8 Hz, Ar**H**), 3.13-3.12 (4H, m, -C**H**$_2$NC**H**$_2$-), 1.57 (4H, m, -C**H**$_2$CH$_2$C**H**$_2$), 1.49-1.48 (4H, m, -CH$_2$C**H**$_2$CH$_2$); ^{13}C-NMR (Bruker, 100 MHz, $CDCl_3$, δ_C, ppm): 166.2 (**Ar**-OH), 162.2 (H**C**=N), 146.1 (**Ar**-N), 140.1; 134.2; 131.7; 131.1; 129.0; 128.8; 127.4; 120.2; 118.7; 118.1 (**Ar**H), 46.3 (-**C**H$_2$N**C**H$_2$-), 25.4 (-**C**H$_2$CH$_2$**C**H$_2$-), 23.6 (-CH$_2$**C**H$_2$CH$_2$-); (GC–MS) *m/z*: calculated for $[C_{18}H_{19}N_3O_5S + H]^+$ 389.1, found 389.0.

2.2.1.2.3. *N^1*-(5^1-bromo-2^1-hydroxybenzylidenyl)-*N*-pyrrolidinyl-2-sulfanilamide **20**

N-Pyrrolidinyl-*o*-sulphanilamide 10 (0.100 g, 0.442 mmol) and 5-bromo-2-salicylaldehyde 15 (0.116 g, 0.575 mmol). Yellow crystals (0.138 g, 76%); R_f = 0.66 (1:1 Ethyl acetate/*n*-hexane); m.p. 143.6–143.8 ºC; IR (Bruker, ATR, *v* cm^{-1}): 3536 (O–H str.) 3070 (*aryl* C–H str.), 2972 (*sp^3*-

C–H str.), 1616 (C=N str.), 1555 (*aryl* C=C str.), 1317 (*asym* SO_2–N str.), 1150 (*sym* SO_2–N str.), 602 (C–Br str.); ^{1}H-NMR (Bruker, 400 MHz, $CDCl_3$, δ_H, ppm): 12.56 (1H, s, O**H**), 8.52 (1H, s, **H**C=N), 7.64 (1H, t, J = 8 Hz, Ar**H**), 7.54 (1H, s, Ar**H**), 7.48 (1H, d, J = 8 Hz, Ar**H**), 7.41 (1H, t, J = 8 Hz, Ar**H**), 7.23 (1H, d, J = 8 Hz, Ar**H**), 6.96 (1H, d, J = 12 Hz, Ar**H**), 3.28 (4H, t, J = 8 Hz, -C**H**2NC**H**2-), 1.83-1.80 (4H, m, -C**H**2C**H**2-); ^{13}C-NMR (Bruker, 100 MHz, $CDCl_3$, δ_C, ppm): 162.6 (**Ar**-OH), 160.0 (H**C**=N), 146.8 (**Ar**-N), 136.6; 134.7; 134.1; 132.2; 130.7; 126.7; 120.5; 120.1; 119.8; 110.6 (**Ar**H), 47.4 (-**C**H2N**C**H2-), 25.5 (-**C**H2**C**H2-);(GC–MS) *m/z*: calculated for $[C_{17}H_{17}B_rN_2O_3S + H]^+$ 408.0, found 408.0.

2.2.1.2.4. *N^1*-(5^1-bromo-2^1-hydroxybenzylidenyl)-*N*-indolinyl-2-sulphanilamide **21**

N-Indolinyl-*o*-sulphanilamide 173 (0.100 g, 0.365 mmol) and 5-bromo-*o*-salicylaldehyde 181 (0.095 g, 0.473 mmol). Yellow oil (0.095 g, 57%); R_f 0.54 (1:1 ethyl acetate/*n*-hexane); IR (Bruker, ATR, v cm^{-1}): 3082 (*aryl* C–H str.), 2901 (*sp^3*-C–H str.), 1612 (C=N str.), 1566; 1516 (*aryl* C=C str.), 1331 (*asym* SO_2–N str.), 1153 (*sym* SO_2–N str.), 612 (C–Br str.); ^{1}H-NMR (Bruker, 400 MHz, $CDCl_3$, δ_H, ppm): 12.31 (1H, s, O**H**), 8.18 (1H, s, **H**C=N), 8.01 (1H, d, J = 8 Hz, Ar**H**), 7.53 (1H, t, J = 8 Hz, Ar**H**), 7.42 (1H, d, J = 8 Hz, Ar**H**), 7.31 (1H, d, J = 12 Hz, Ar**H**), 7.20 (2H, d, J = 8 Hz, Ar**H**), 7.01 (1H, d, J = 8 Hz, Ar**H**), 6.98 (1H, d, J = 4 Hz, Ar**H**), 6.89 (1H, d, J = 8 Hz, Ar**H**), 6.77 (1H, d, J = 10 Hz, Ar**H**), 6.75 (1H, d, J = 4 Hz, Ar**H**), 3.94 (2H, t, J = 8 Hz, -NC**H**2-), 2.78 (2H, t, -NCH2C**H**2-); ^{13}C-NMR (Bruker, 100 MHz, $CDCl_3$, δ_C, ppm): 163.0 (**Ar**-OH), 160.0 (H**C**=N), 147.1 (**Ar**-N), 141.6, 136.7, 134.6; 134.5; 132.2; 131.4; 130.7; 127.5; 126.7; 124.9; 123.5; 120.7; 119.8; 114.7; 110.6; (**Ar**H), 50.0 (-N**C**H2-), 28.0 (-NCH2**C**H2-);(GC–MS) *m/z*: calculated for $[C_{21}H_{17}BrN_2O_3S + H]^+$ 456.0, found 456.0.

2.2.1.2.5.*N*[1]-(5[1]-bromo-2[1]-hydroxybenzylidenyl)-*N*-1,3,4-trihydroisoquinolinyl-2-sulphanilamide **22**

N-Isoquinolinyl-*o*-sulphanilamide 174 (0.100 g, 0.347 mmol) and 5-bromo-2-salicylaldehyde 181 (0.091 g, 0.453 mmol). Yellow crystals (0.120 g, 73.5%); R_f 0.38 (1:1 $CHCl_3$/*n*-hexane); m.p. 138.2–138.4 ºC; IR (Bruker, ATR, v cm^{-1}): 3235 (C-OH str.), 3044 (*aryl* C–H str.), 2982 (sp^3-C–H str.), 1670 (C=N str.), 1636;1564 (*aryl* C=C str.), 1373 (*asym* SO_2–N str.), 1153 (*sym* SO_2–N str.), 625 (C–Br str.); ^{1}H-NMR (Bruker, 400 MHz, $CDCl_3$, δ_H, ppm): 12.45 (1H, s, O**H**), 8.12 (1H, s, **H**C=N), 8.03 (1H, d, J = 8 Hz, Ar**H**), 7.54 (1H, t, J = 8 Hz, Ar**H**), 7.38 (1H, d, J = 8Hz, Ar**H**), 7.33 (1H, t, J = 8Hz, Ar**H**), 7.19 (1H, d, J = 8 Hz, Ar**H**), 7.02 (1H, t, J = 8 Hz, Ar**H**), 6.91 (1H, s, Ar**H**), 6.88 (2H, d, J = 8 Hz, Ar**H**), 6.67 (1H, d, J = 8 Hz, Ar**H**), 4.33 (2H, s, -NC**H**$_2$-), 3.41 (2H, t, J = 6 Hz, -NC**H**$_2$-), 2.56 (2H, t, J = 6 Hz, -NCH$_2$C**H**$_2$-); ^{13}C-NMR (Bruker, 100 MHz, $CDCl_3$, δ_C, ppm): 162.9 (**Ar**-OH), 160.0 (H**C**=N), 146.9 (**Ar**-N), 136.6, 134.8, 134.4; 133.0; 132.2; 131.9; 131.1; 128.8; 126.8; 126.6; 126.3; 126.2; 120.4; 120.4; 119.7; 110.6; (**Ar**H), 46.7 (-N**C**H$_2$-), 42.8 (-N**C**H$_2$-), 28.2 (-NCH$_2$**C**H$_2$-); (GC–MS) *m/z*: calculated for $[C_{22}H_{19}BrN_2O_3S + H]^+$ 470.0, found 470.0.

2.2.1.2.5. *N*[1]-(2[1]-hydroxy-5[1]-nitrobenzylidenyl)-*N*-1,3,4-trihydroisoquinolinyl-2-sulphanilamide **23**

N-Isoquinolinyl-*o*-sulphanilamide 174 (0.100 g, 0.347 mmol) and 5-nitro-2-salicylaldehyde 182 (0.075 g, 0.451 mmol). Yellow crystals (0.112 g, 74%); R_f 0.45 (2:1 CH_2Cl_2/*n*-hexane); m.p. 168.5–168.7 ºC; IR (Bruker, ATR, v cm^{-1}): 3080 (*aryl* C–H str.), 2901 (sp^3-C–H str.), 1614 (C=N str.), 1566 (*aryl* C=C str.), 1330 (*asym* SO_2–N str.), 1152 (*sym* SO_2–N str.), 1516 (*asym* NO_2 str.), 1330 (*sym* NO_2 str.); ^{1}H-NMR (Bruker, 400 MHz, $CDCl_3$, δ_H, ppm): 13.47 (1H, s, O**H**), 8.37 (1H,

s, **H**C=N), 8.30 (1H, d, *J* = 8 Hz, Ar**H**), 8.17 (1H, s, Ar**H**), 8.15 (1H, d, *J* = 8 Hz, Ar**H**), 7.69 (1H, t, *J* = 8 Hz Ar**H**), 7.50 (1H, t, *J* = 8 Hz, Ar**H**), 7.19 (1H, d, *J* = 8 Hz, Ar**H**), 7.16 (1H, d, *J* = 8 Hz, Ar**H**), 7.09 (1H, t, *J* = 8 Hz, Ar**H**), 7.00 (1H, d, *J* = 8 Hz, ArH), 6.95 (1H, t, *J* = 8 Hz, Ar**H**), 6.76 (1H, d, *J* = 8 Hz, Ar**H**), 4.46 (2H, s, -NC**H**$_2$-), 3.52 (2H, t, *J* = 6 Hz, -NC**H**$_2$-), 2.66 (2H, t, *J* = 8 Hz -NCH$_2$C**H**$_2$-); ^{13}C-NMR (Bruker, 100 MHz, CDCl$_3$, δ_C, ppm): 166.1 (**Ar**-OH), 162.5 (H**C**=N), 146.2 (**Ar**-N), 140.1, 140.1, 134.5, 132.9; 132.4; 131.8; 131.1; 129.0; 128.8; 127.4; 126.7; 126.3; 126.1; 120.3; 118.6; 118.0; (**Ar**H), 46.7 (-N**C**H$_2$-), 42.7 (-N**C**H$_2$-), 28.1 (-NCH$_2$**C**H$_2$-); (GC–MS) *m/z*: calculated for $[C_{22}H_{19}N_3O_5S + H]^+$ 437.1, found 437.1.

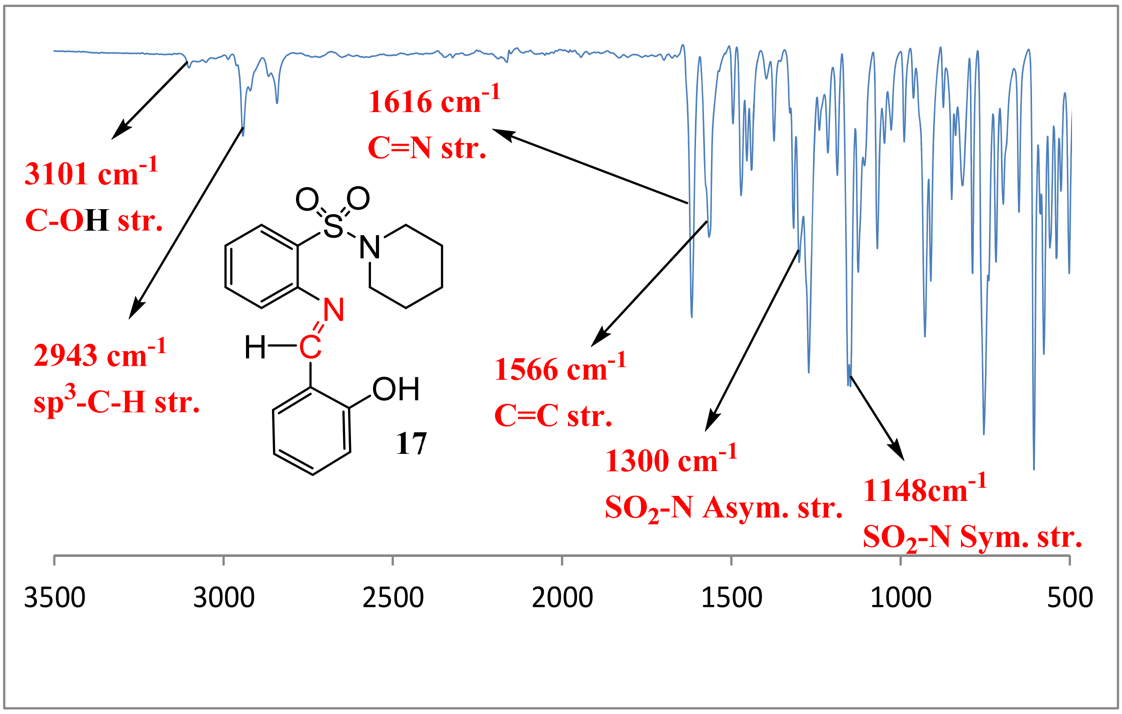

FTIR spectrum of *N*^{1}-(2^1-hydroxybenzylidenyl)-*N*-piperidinyl-2-sulfanilamide **17**

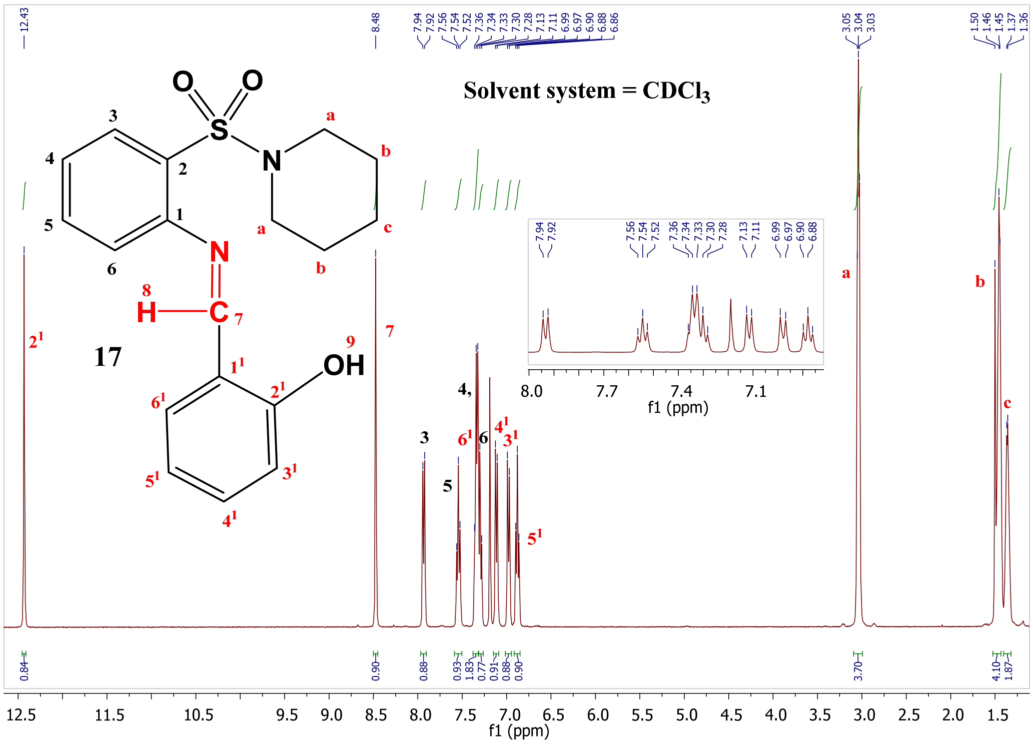

^{1}H NMR spectrum of N^{1}-(2^{1}-hydroxybenzylidenyl)-*N*-piperidinyl-2-sulfanilamide **17**

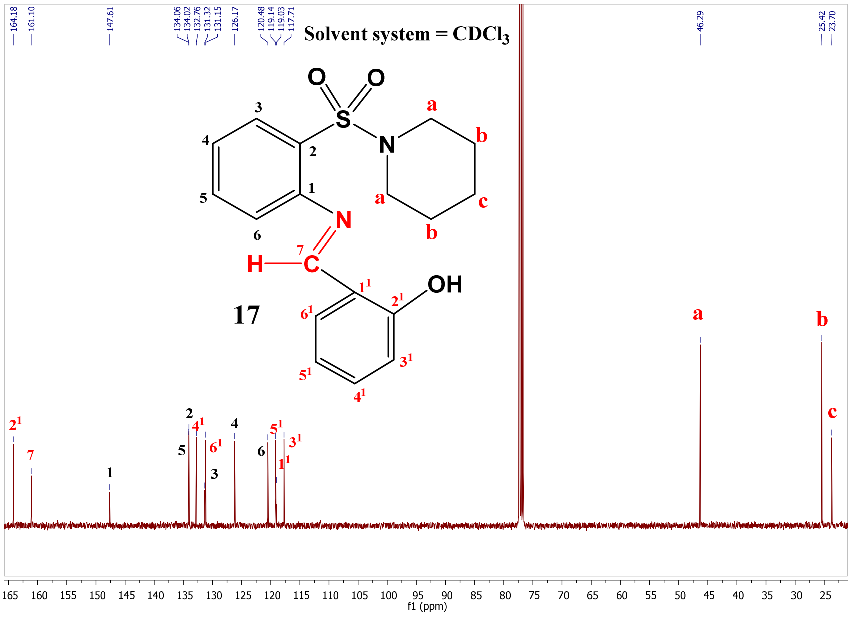

^{13}C NMR spectrum of N^{1}-(2^{1}-hydroxybenzylidenyl)-*N*-piperidinyl-*o*-sulfanilamide **17**

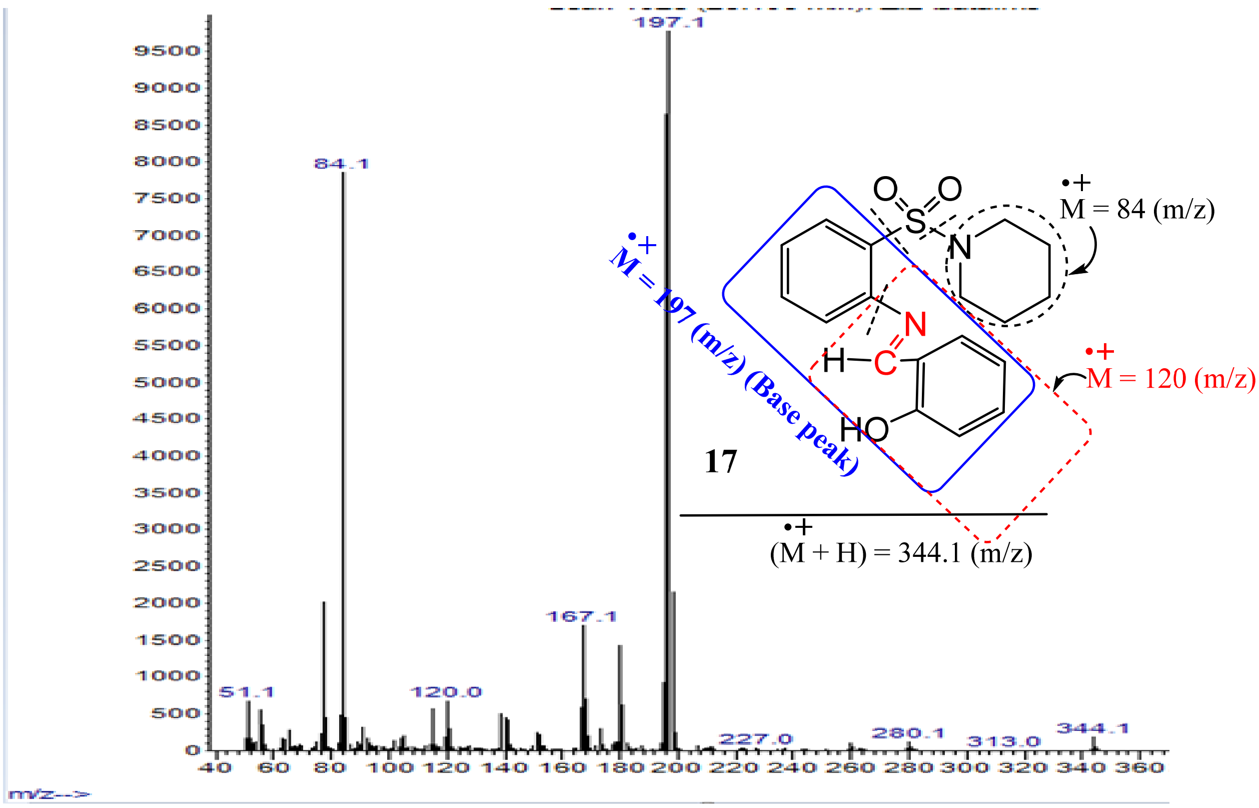

MS spectrum of N^{1}-(2^{1}-hydroxybenzylidenyl)-*N*-piperidinyl-*o*-sulfanilamide **17**

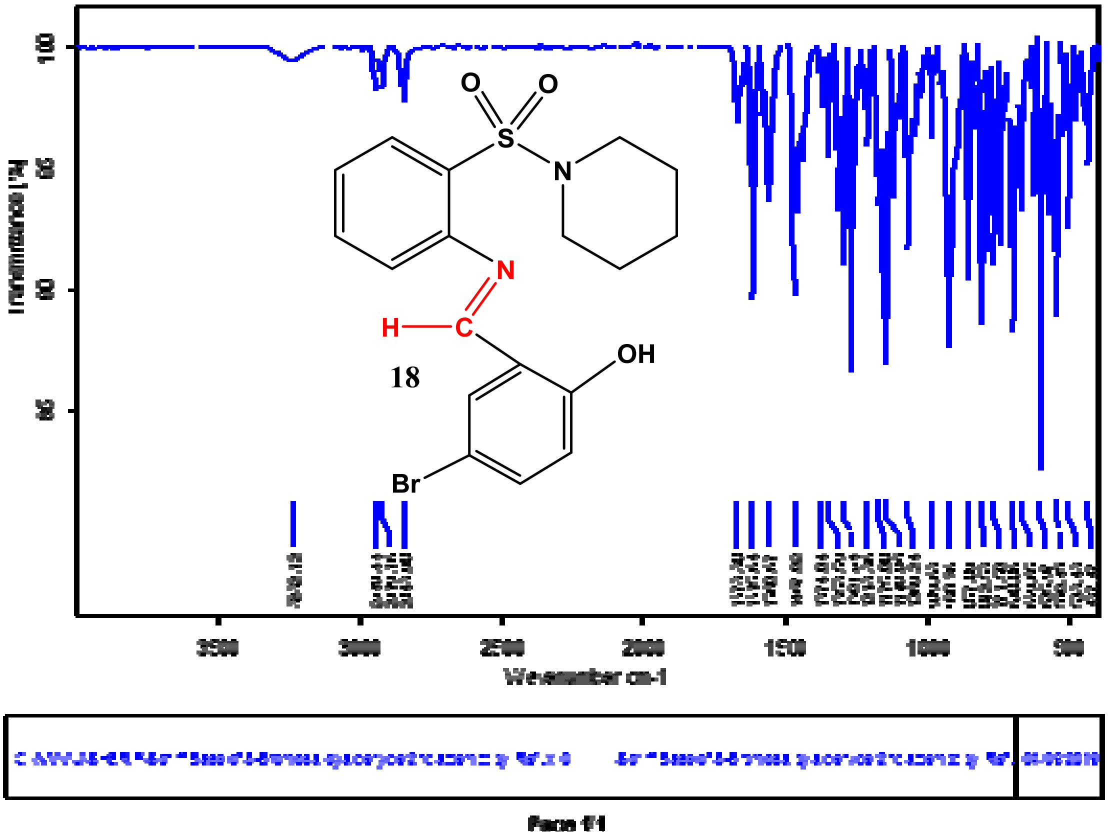

FTIR N^{1}-(5[1]-bromo-2[1]-hydroxybenzylidenyl)-*N*-piperidinyl-2-sulfanilamide **18**

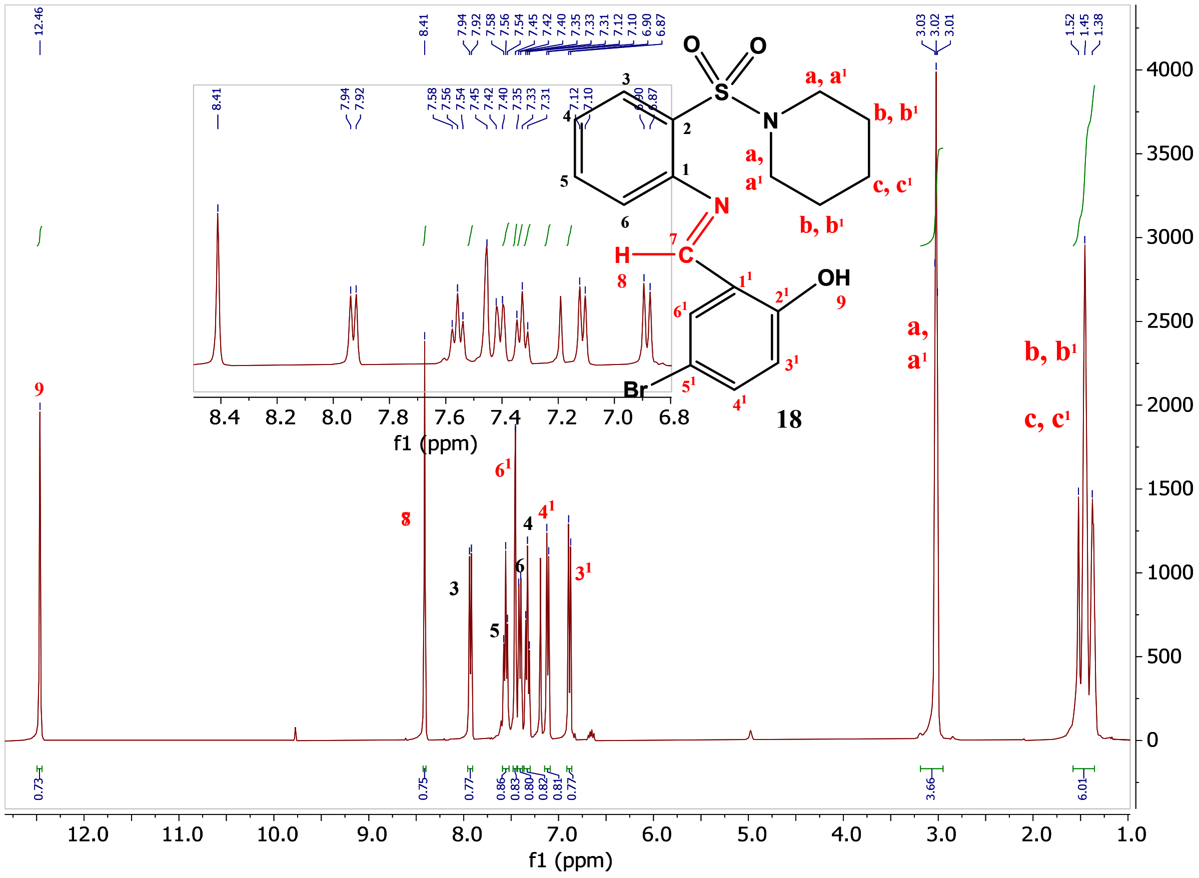

^{1}H NMR spectrum of N^1-(5[1]-bromo-2[1]-hydroxybenzylidenyl)-*N*-piperidinyl-2-sulfanilamide **18**

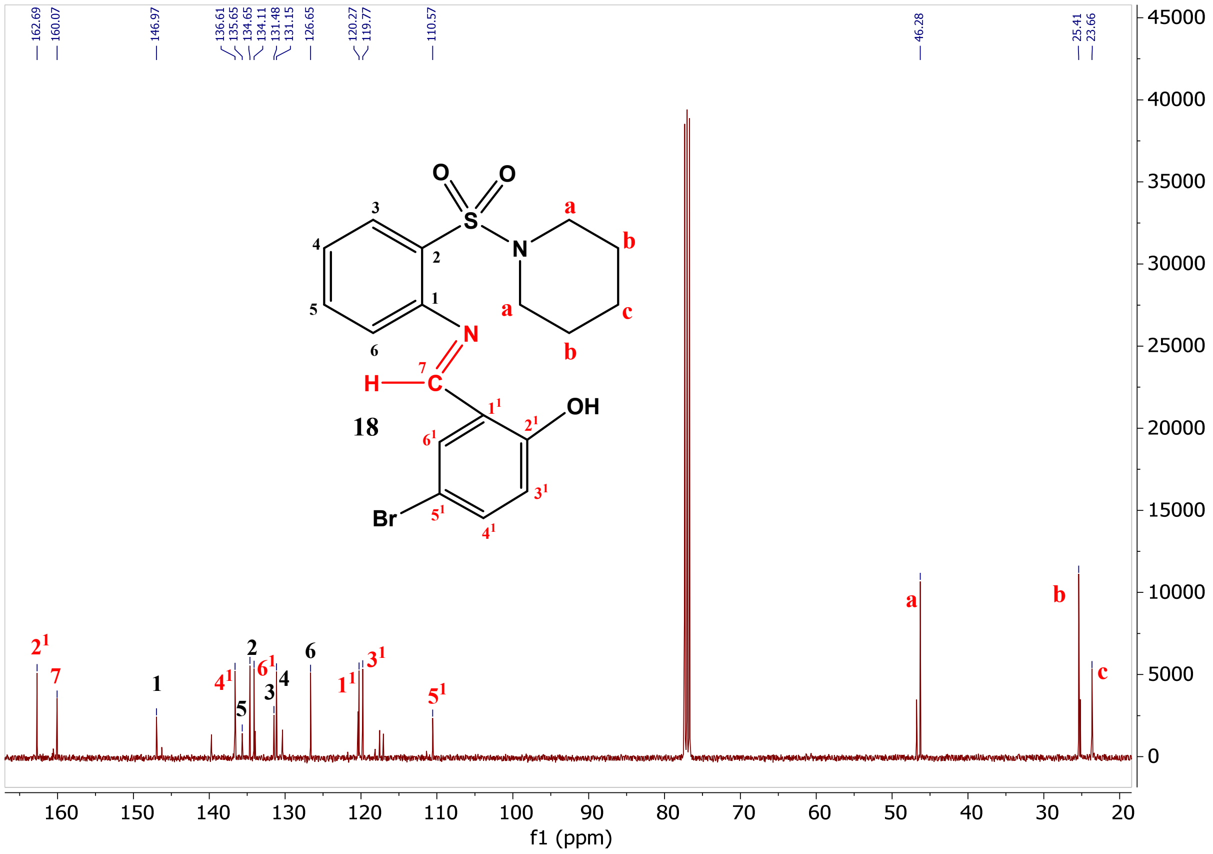

^{13}C NMR spectrum of *N*^{1}-(5^{1}-bromo-2^{1}-hydroxybenzylidenyl)-*N*-piperidinyl-2-sulfanilamide **18**

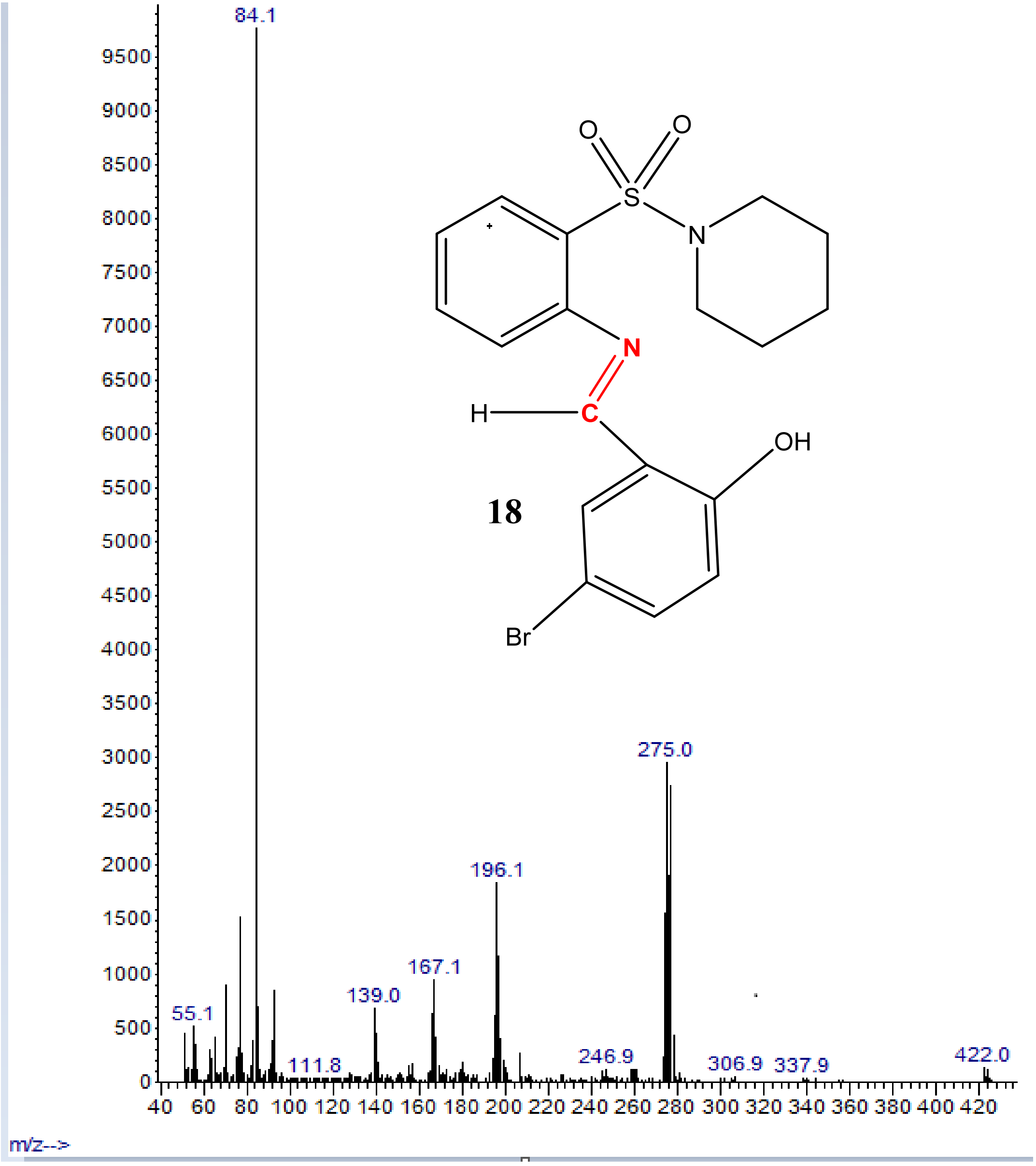

MS (ESI) spectrum of *N*[1]-(5[1]-bromo-2[1]-hydroxybenzylidenyl)-*N*-piperidinyl-2-sulfanilamide **18**

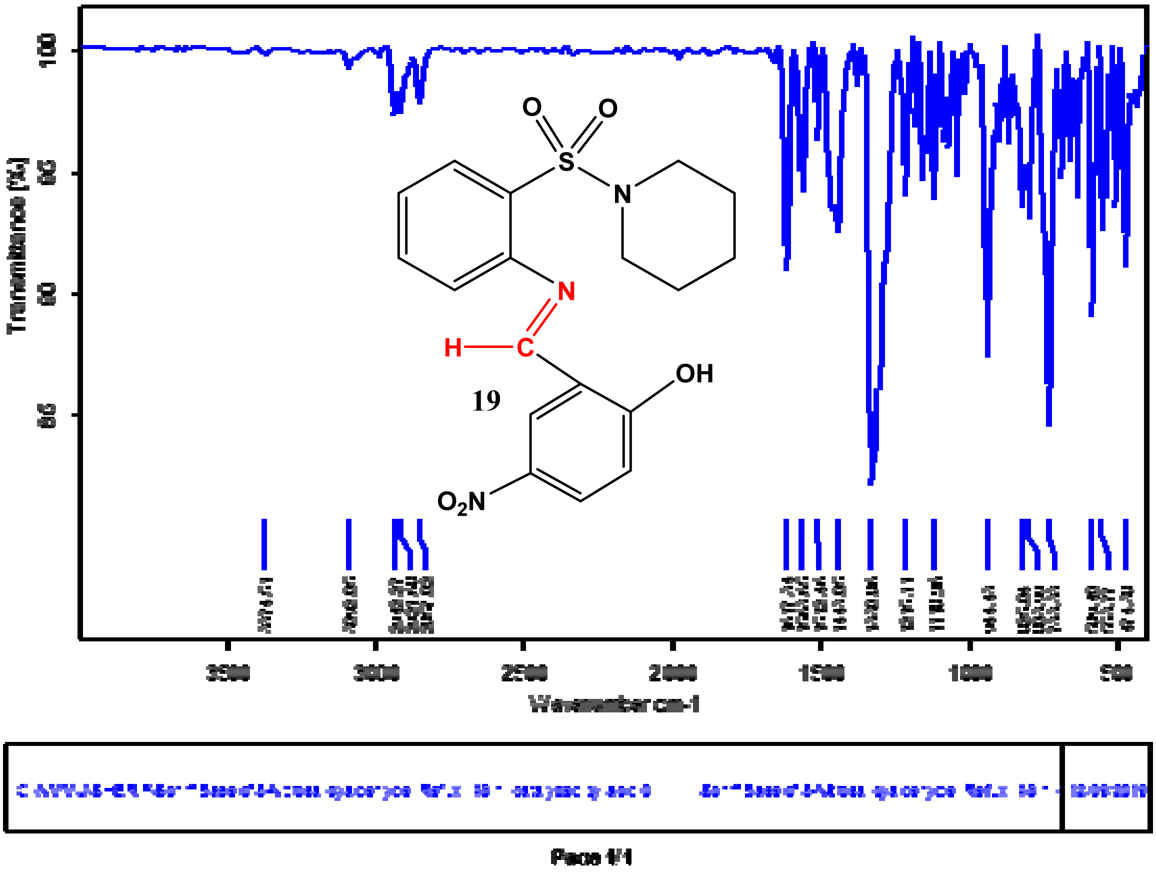

FTIR spectrum of N^{1}-(2^{1}-hydroxy-5^{1}-nitrobenzylidenyl)-*N*-piperidinyl-2-sulfanilamide **19**

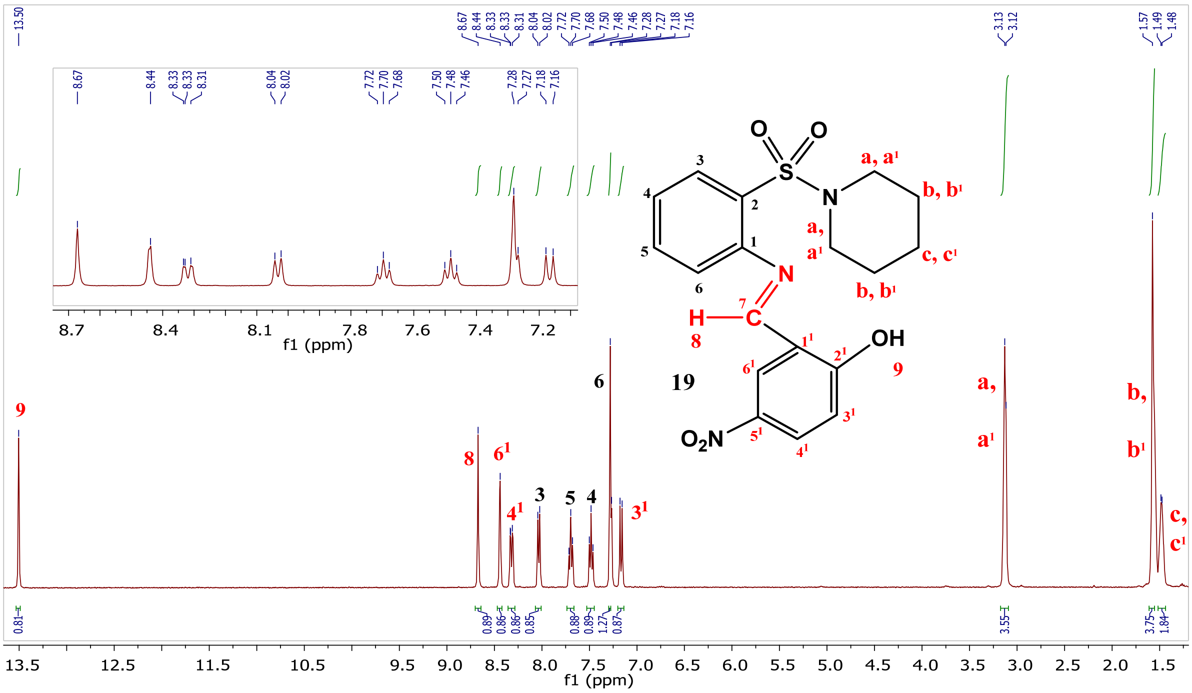

^{1}H NMR spectrum of *N^{1}*-(2^{1}-hydroxy-5^{1}-nitrobenzylidenyl)-*N*-piperidinyl-2-sulfanilamide **19**

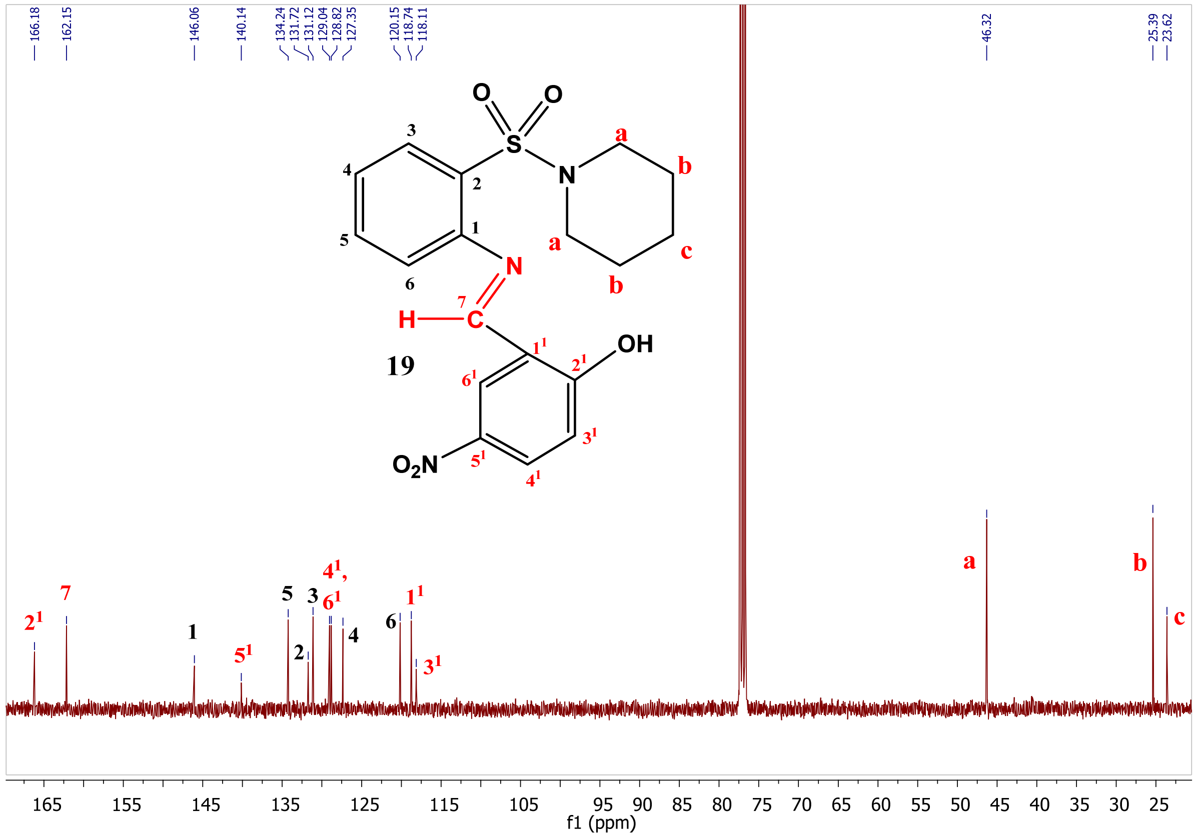

^{13}C NMR spectrum of N^1-(2^1-hydroxy-5^1-nitrobenzylidenyl)-*N*-piperidinyl-2-sulfanilamide **19**

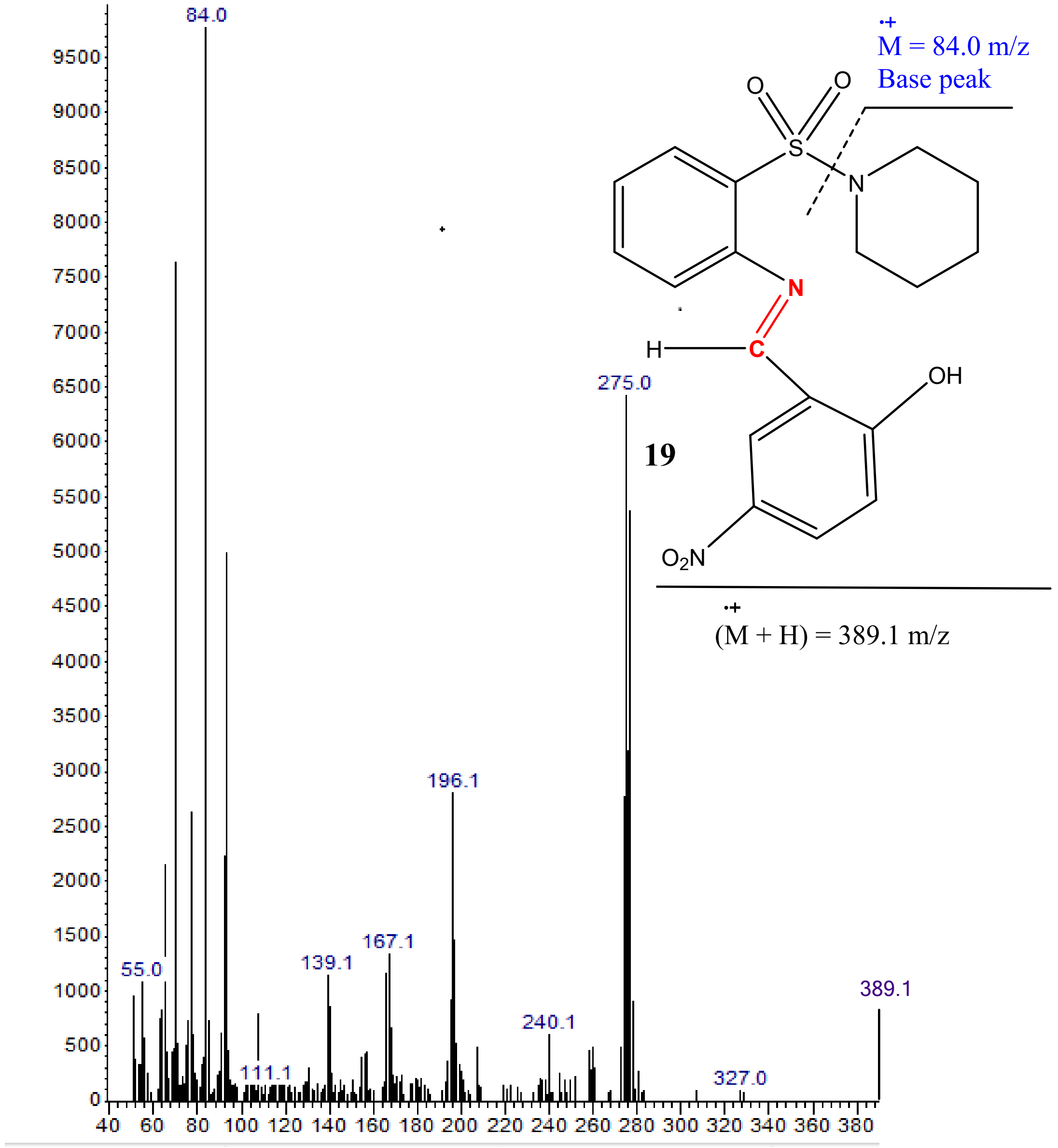

MS (ESI) spectrum of *N*[1]-(2[1]-hydroxy-5[1]-nitrobenzylidenyl)-*N*-piperidinyl-2-sulfanilamide **19**

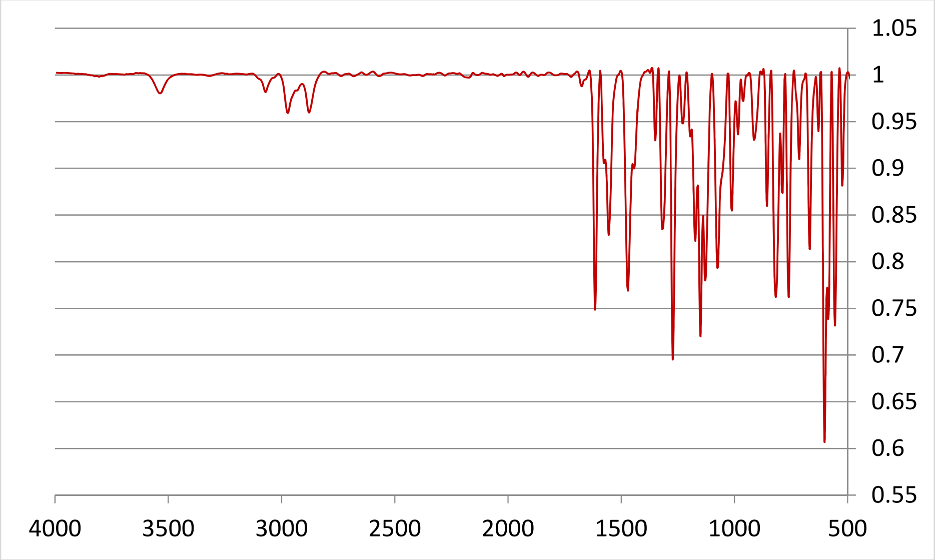

FTIR spectrum of *N*^{1}-(5^{1}-bromo-2^{1}-hydroxybenzylidenyl)-*N*-pyrrolidinyl-2-sulfanilamide **20**

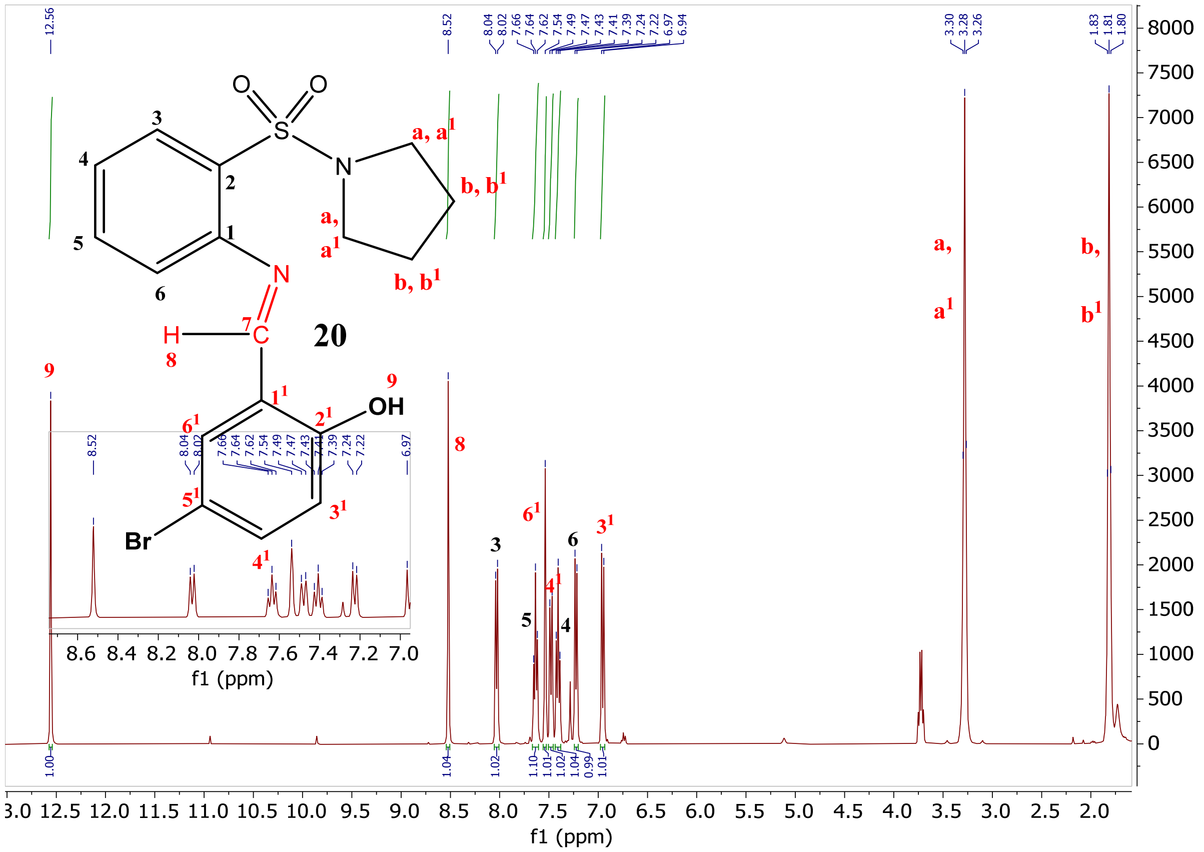

^{1}H NMR spectrum of *N[1]*-(5[1]-bromo-2[1]-hydroxybenzylidenyl)-*N*-pyrrolidinyl-2-sulfanilamide **20**

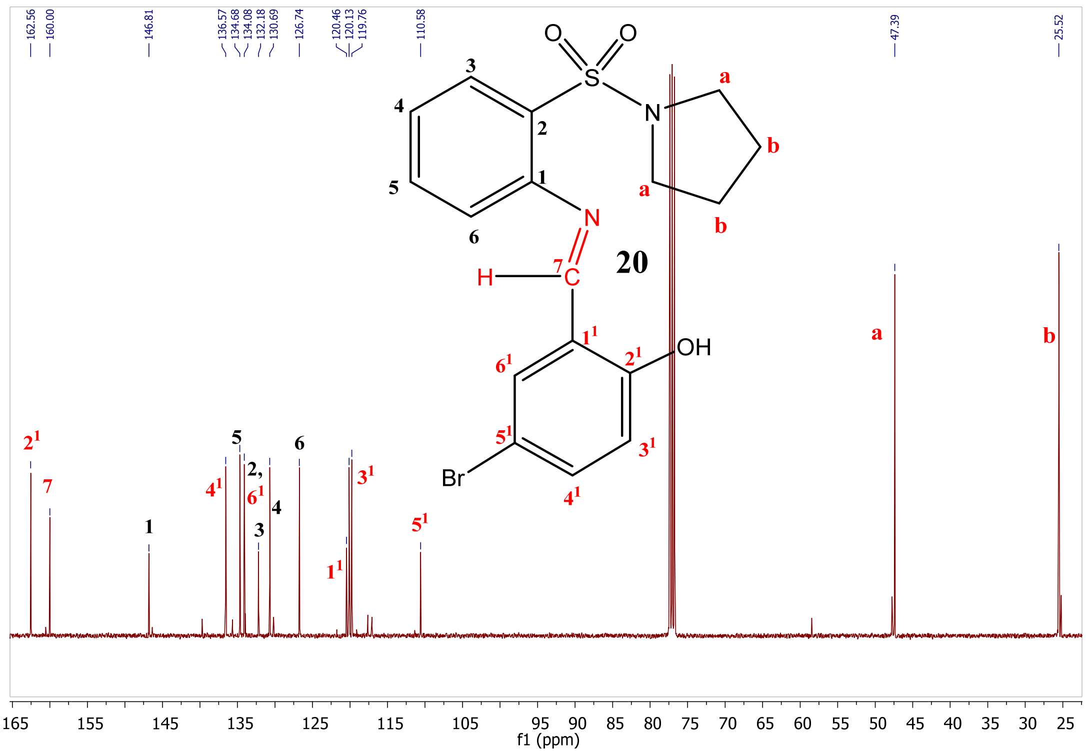

^{13}C NMR spectrum of *N*^{1}-(5^{1}-bromo-2^{1}-hydroxybenzylidenyl)-*N*-pyrrolidinyl-2-sulfanilamide **20**

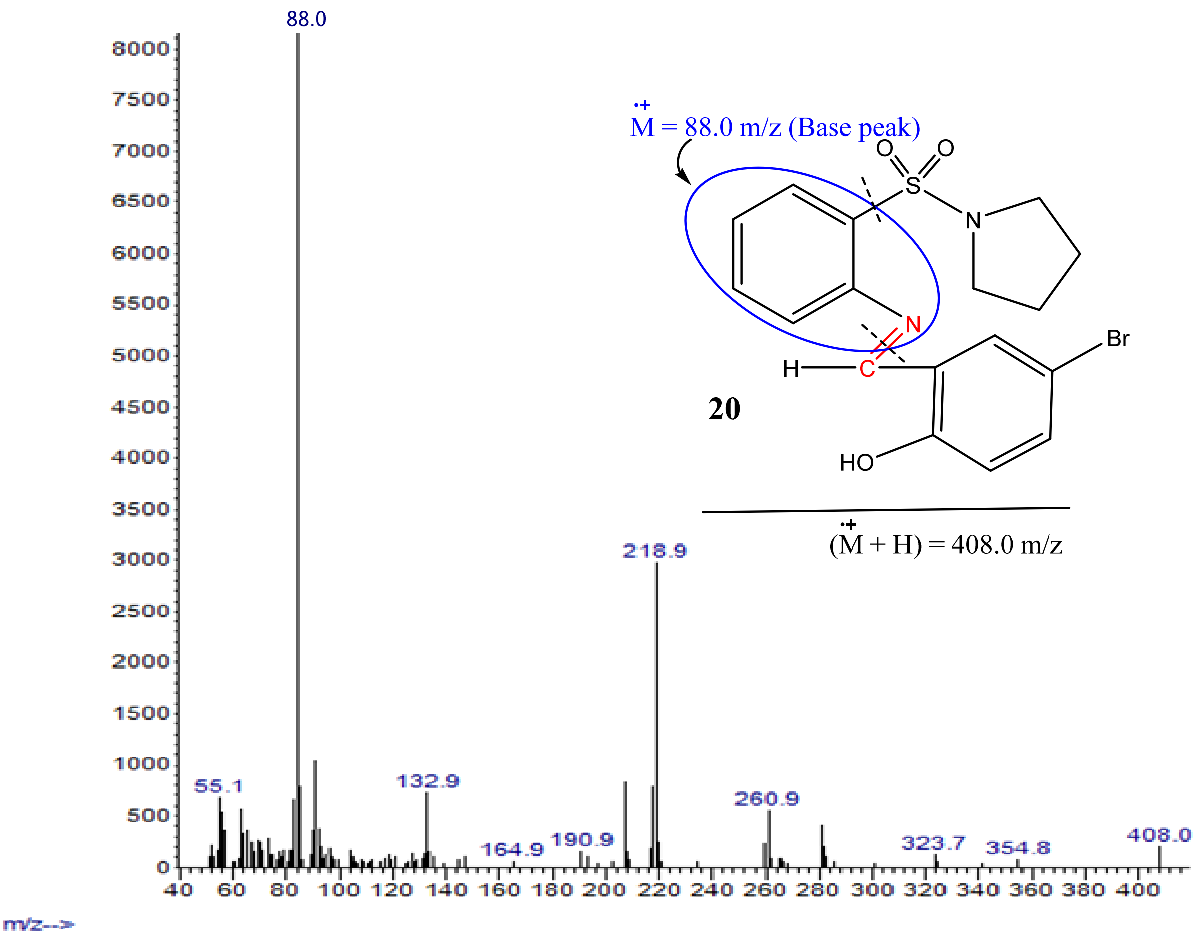

MS (ESI) spectrum of N^1-(5[1]-bromo-2[1]-hydroxybenzylidenyl)-*N*-pyrrolidinyl-2-sulfanilamide **20**

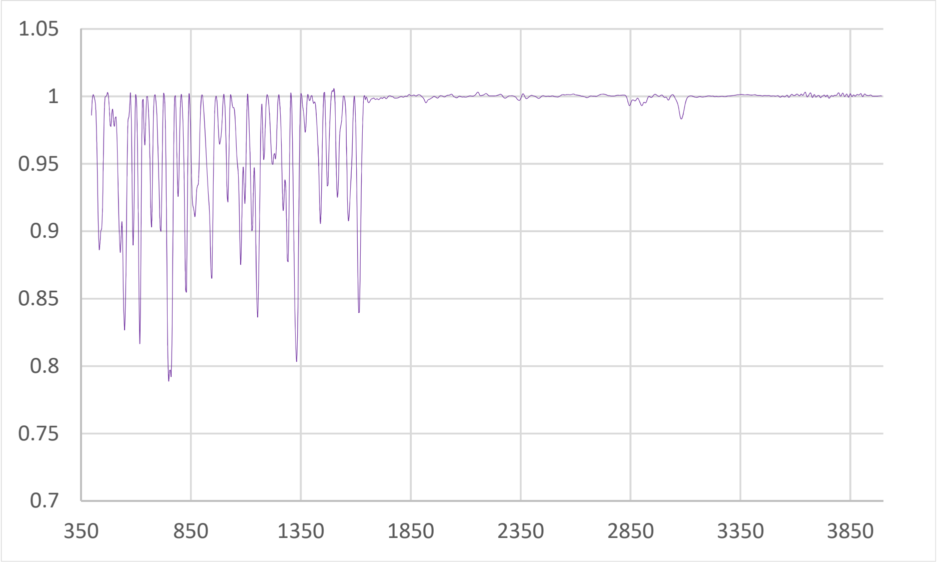

FTIR spectrum of N^{1}-(5[1]-bromo-2[1]-hydroxybenzylidenyl)-*N*-indolinyl-2-sulfanilamide **21**

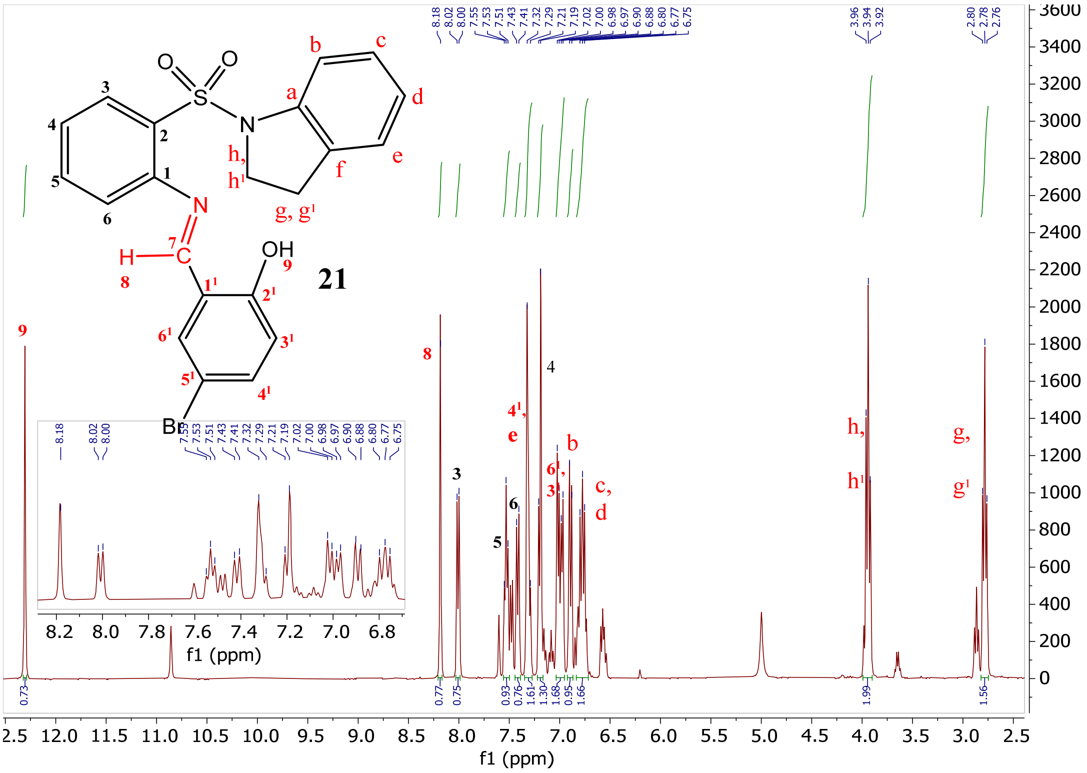

^{1}H NMR spectrum of N^{1}-(5[1]-bromo-2[1]-hydroxybenzylidenyl)-*N*-indolinyl-2-sulfanilamide **21**

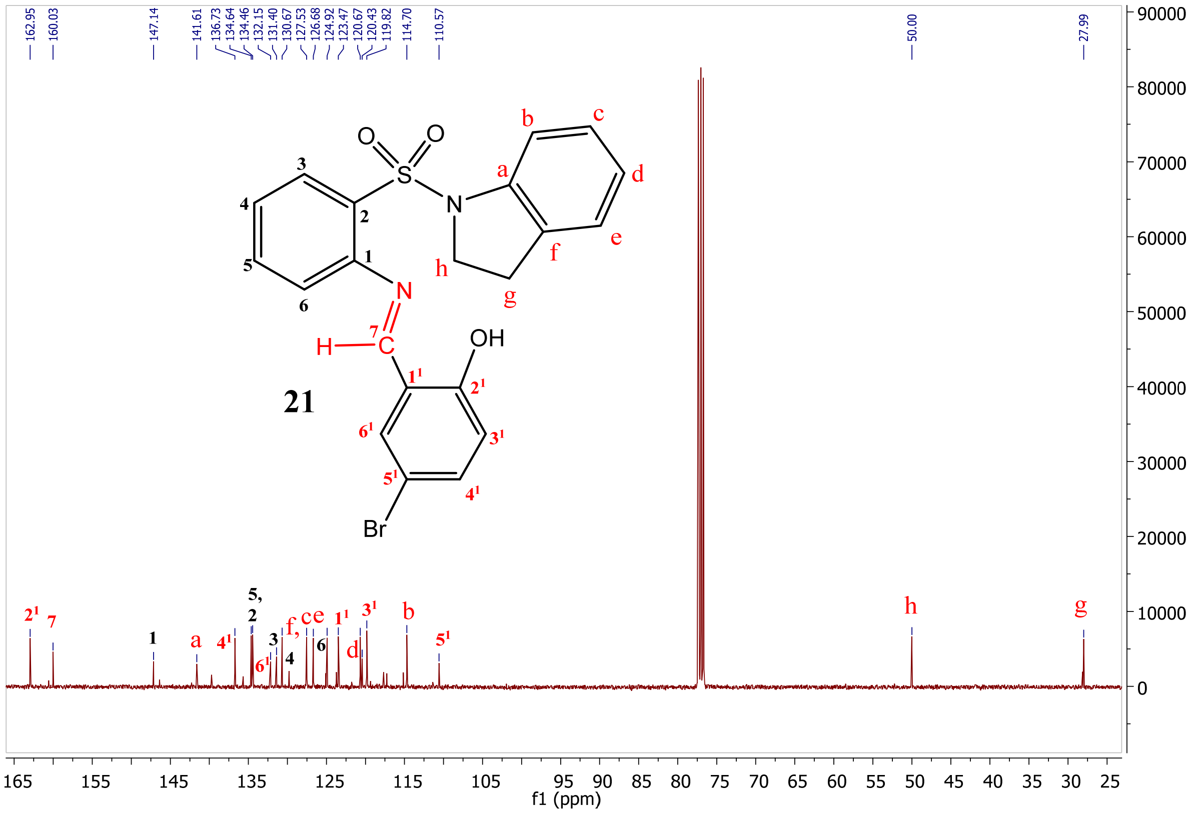

^{13}C NMR spectrum of N^{1}-(5^{1}-bromo-2^{1}-hydroxybenzylidenyl)-*N*-indolinyl-2-sulfanilamide **21**

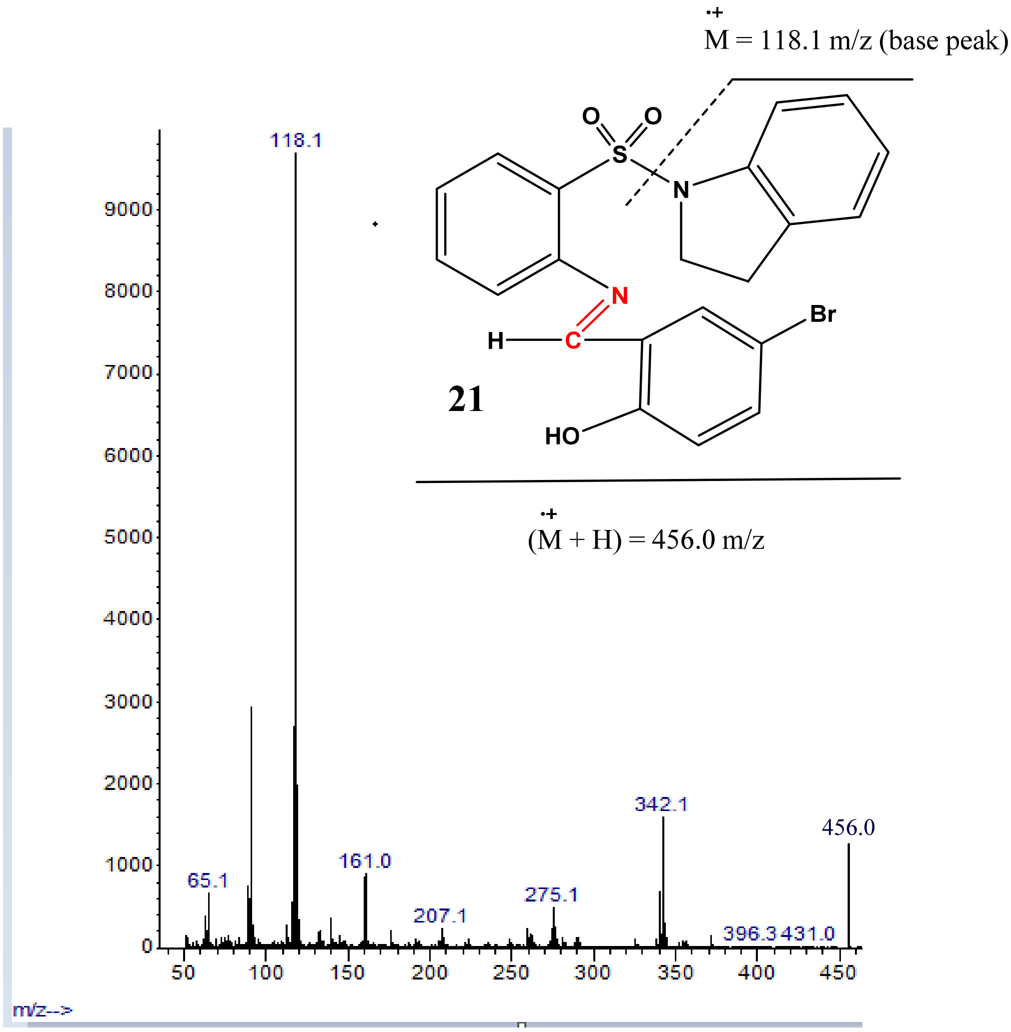

MS spectrum of *N*[1]-(5[1]-bromo-2[1]-hydroxybenzylidenyl)-*N*-indolinyl-2-sulfanilamide **21**

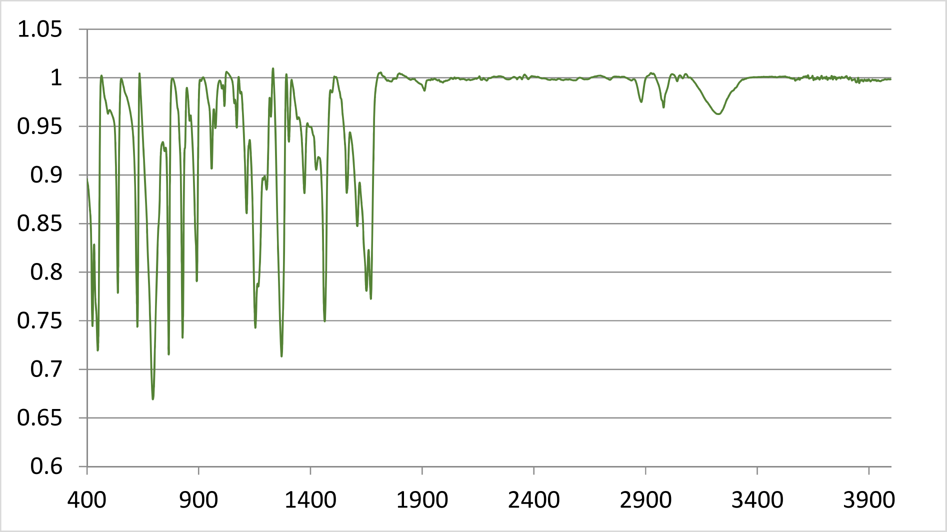

FTIR spectrum of N^1-(5[1]-bromo-2[1]-hydroxybenzylidenyl)-*N*-trihydroisoquinolinyl-2-sulfanilamide **22**

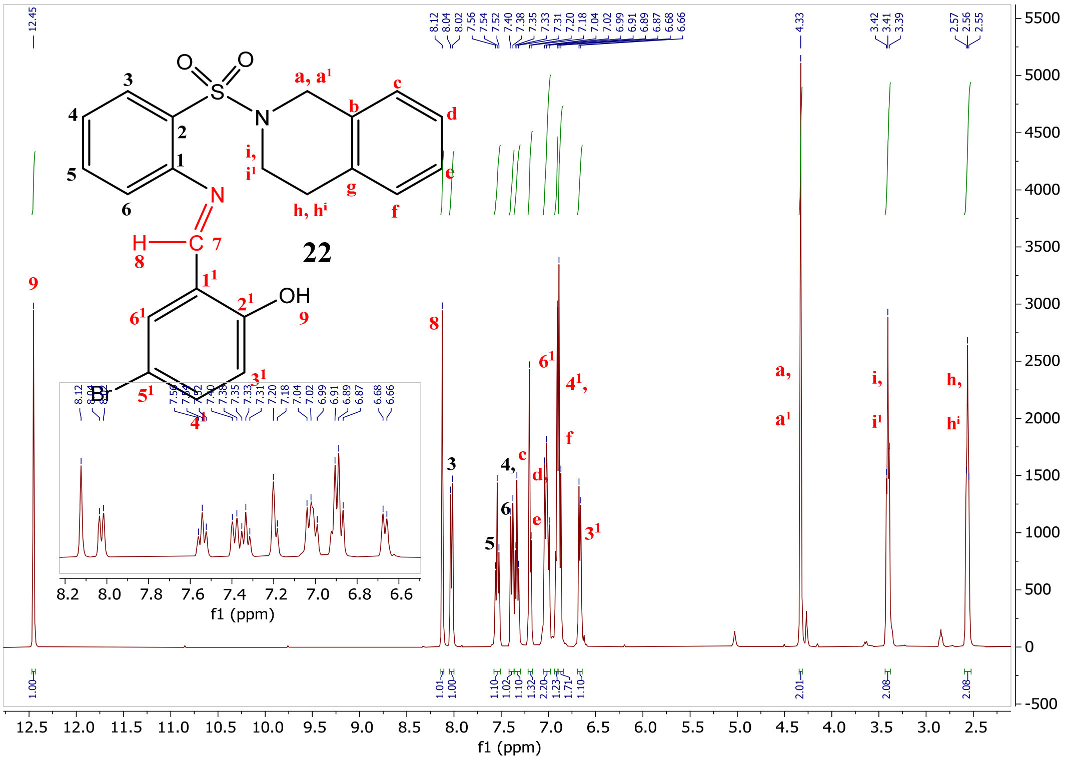

^{1}H NMR spectrum of N^1-(5[1]-bromo-2[1]-hydroxybenzylidenyl)-*N*-trihydroisoquinolinyl-2-sulfanilamide **22**

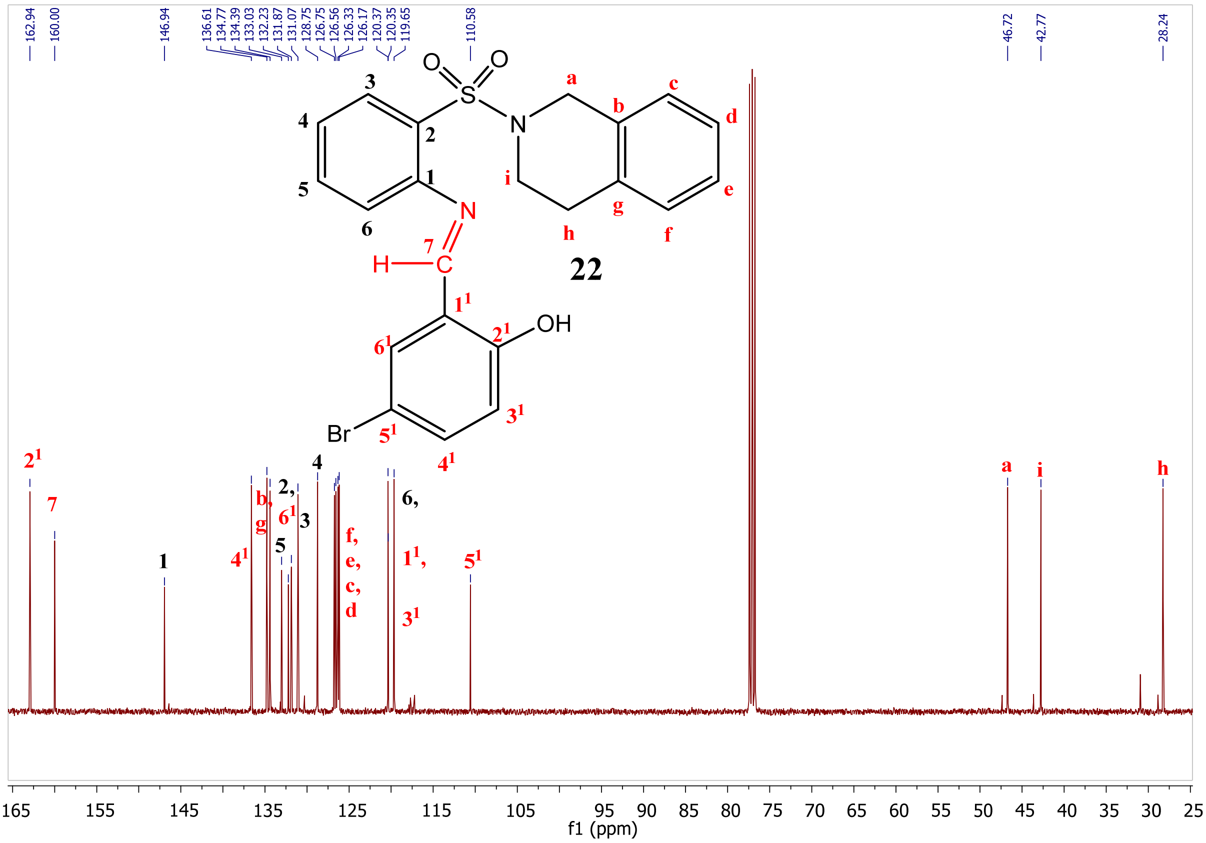

^{13}C NMR spectrum of N^{1}-(5^{1}-bromo-2^{1}-hydroxybenzylidenyl)-*N*-trihydroisoquinolinyl-2-sulfanilamide **22**

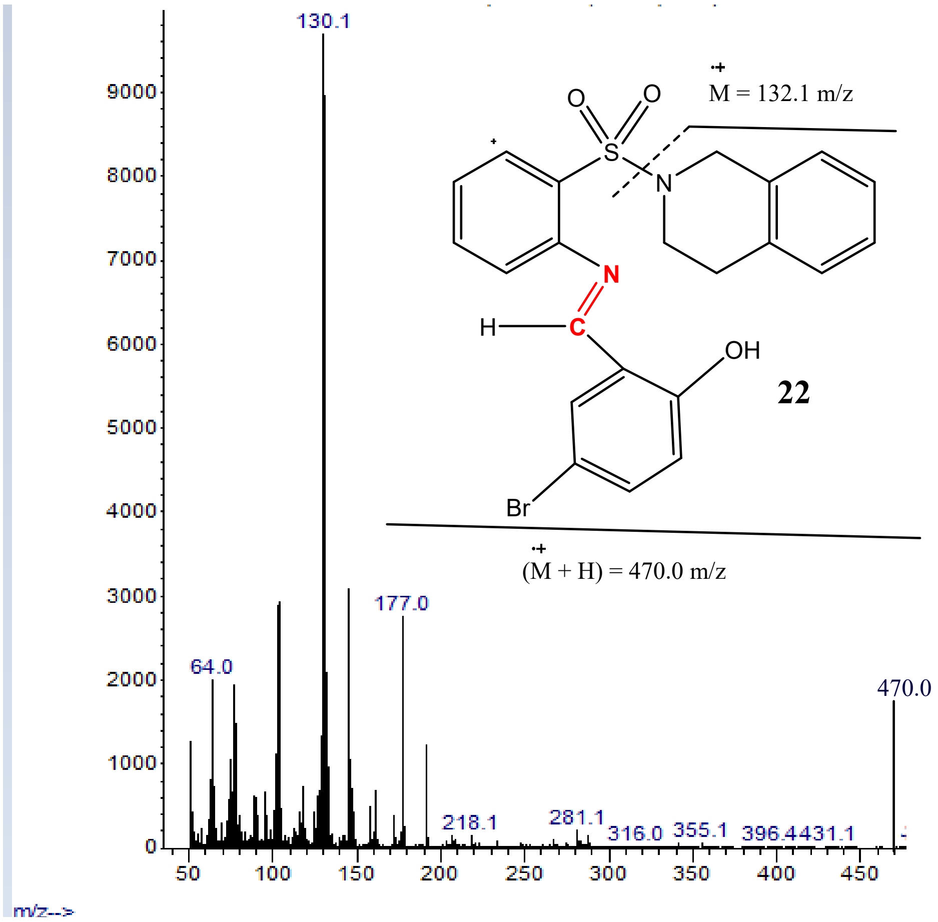

MS (ESI) spectrum of N^1-(5^1-bromo-2^1-hydroxybenzylidenyl)-*N*-trihydroisoquinolinyl-2-sulfanilamide **22**

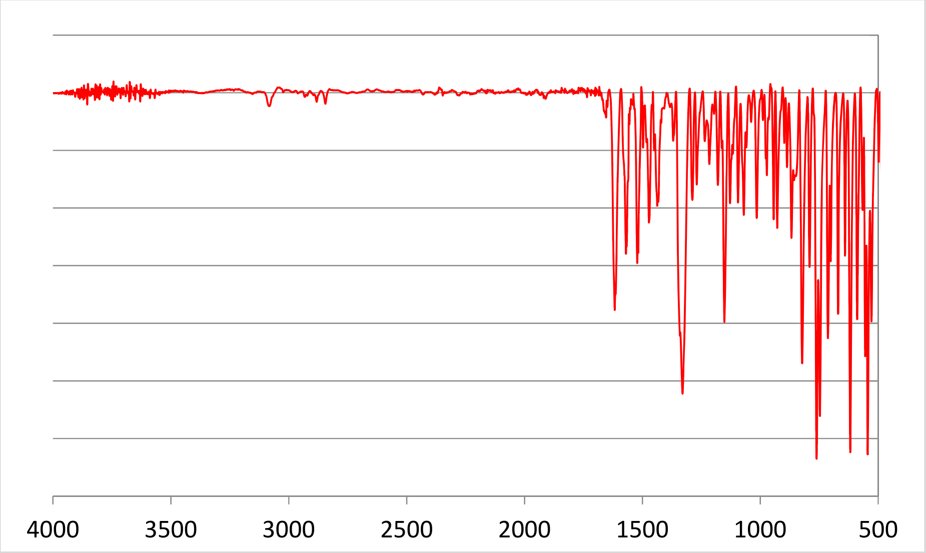

FTIR spectrum of *N*^{1}-(2^{1}-hydroxy-5^{1}-nitrobenzylidenyl)-*N*-trihydroisoquinolinyl-2-sulfanilamide **23**

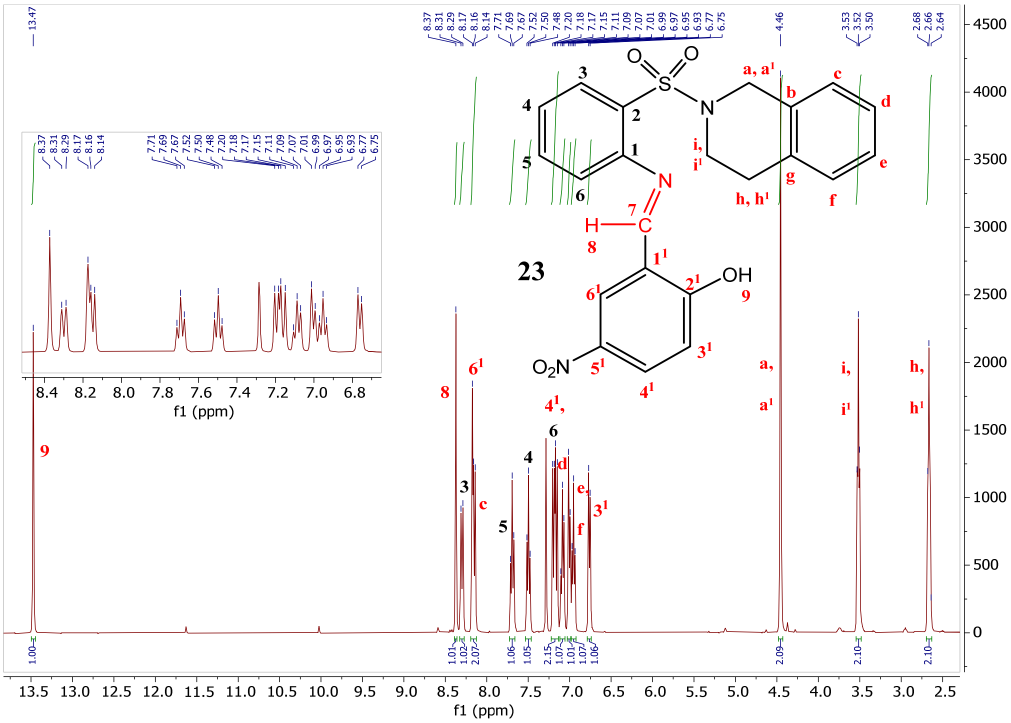

^{1}H NMR spectrum of *N*^{1}-(2^{1}-hydroxy-5^{1}-nitrobenzylidenyl)-*N*-trihydroisoquinolinyl-2-sulfanilamide **23**

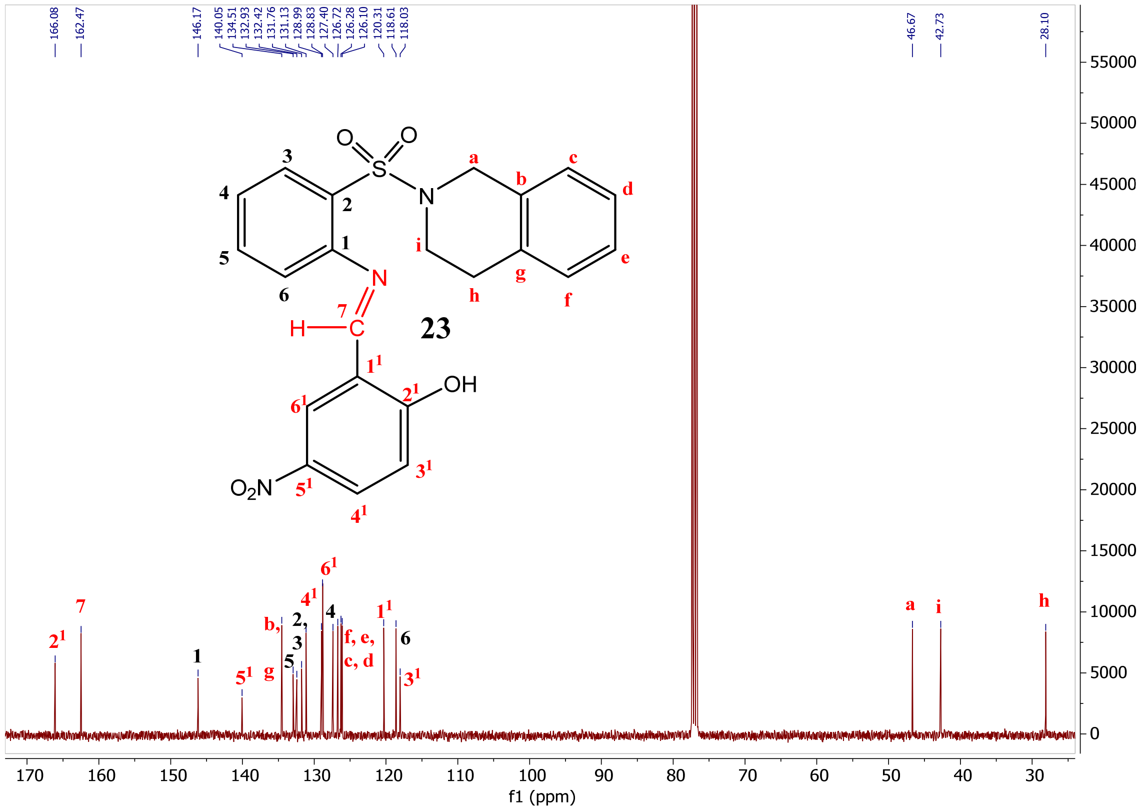

^{13}C NMR spectrum of *N*[1]-(2[1]-hydroxy-5[1]-nitrobenzylidenyl)-*N*-trihydroisoquinolinyl-2-sulfanilamide **23**

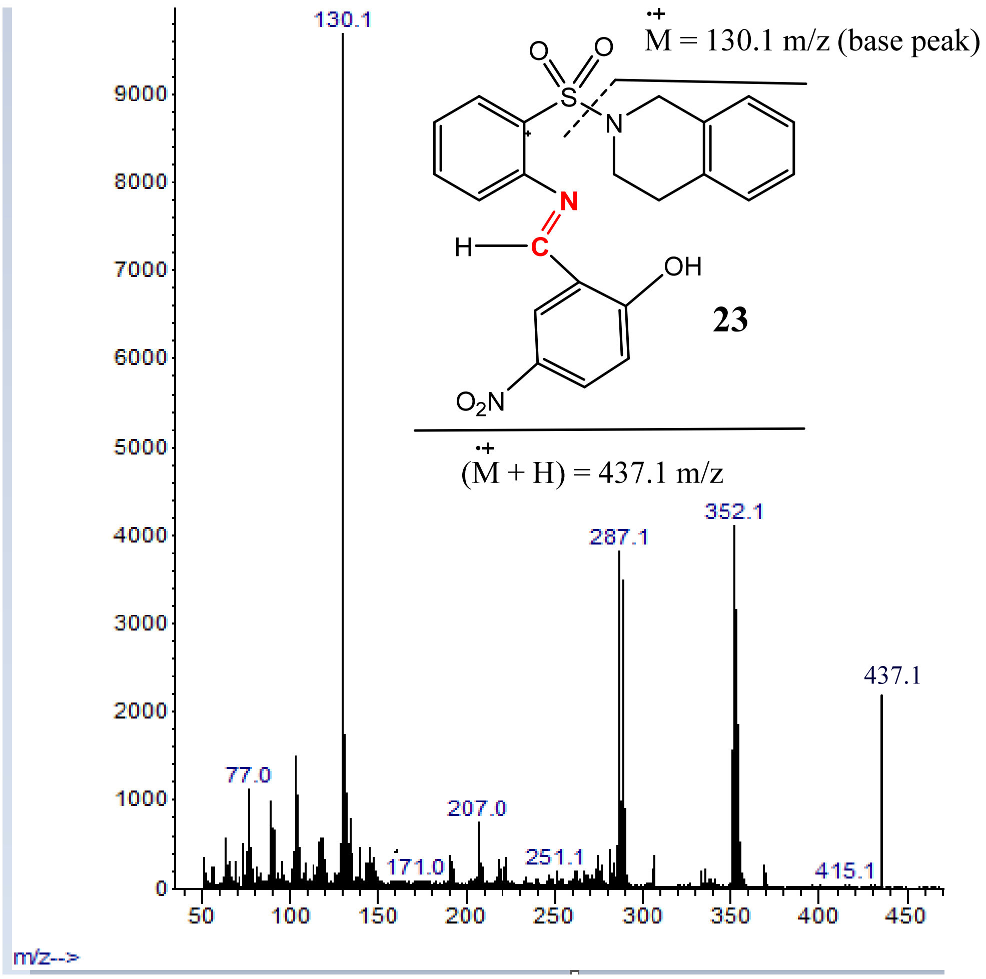

MS spectrum of N^{1}-(2^{1}-hydroxy-5^{1}-nitrobenzylidenyl)-*N*-trihydroisoquinolinyl-2-sulfanilamide **23**

Table 1: Physical and analytical properties of Sulfonamide Schiff Bases **17-23**

Compd.	Colour and Nature	Molecular formula	M.wt (g/mol)	Yield (%)	M.pt. (°C)
**17**	Yellow crystals	$C_{18}H_{20}N_2O_3S$	343.1	82	153.0-155.0
**18**	Yellow crystals	$C_{18}H_{19}BrN_2O_3S$	422.0	85	154.2-154.4
**19**	Yellow crystals	$C_{18}H_{19}N_3O_5S$	388.0	80	159.2-159.4
**20**	Yellow crystals	$C_{17}H_{17}BrN_2O_3S$	407.0	76	143.6-143.8
**21**	Yellow oil	$C_{21}H_{17}BrN_2O_3S$	456.0	62	N/A
**22**	Yellow crystals	$C_{22}H_{19}BrN_2O_3S$	470.0	83.5	162.2-162.4
**23**	Yellow crystals	$C_{22}H_{19}N_3O_5S$	436.0	87	168.5-168.7

Table 2: Some selected spectroscopic data of Sulfonamide Schiff Bases **17-23**

Compd.	IR *v*(C=N)	IR (cm^{-1}) *v*Ar(C=C)	IR *v*(SO_2-N)	^{1}H-NMR (δ) **H**C=N; C-O**H** ppm	^{13}C-NMR (δ) H**C**=N; **C**-OH ppm	MS [M + H]$^+$
**17**	1616	1566	1300-1148	H**8** = 8.48, s, 1H; H**9** = 12.43, s, 1H.	C**1** = 164.18; C**7** = 161.10.	343.1
**18**	1618	1561	1308-1136	H**8** = 8.50, s, 1H; H**9** = 12.56, s, 1H.	C**1** = 162.70; C**7** = 160.10.	422.0
**19**	1617	1512	1331-1119	H**8** = 8.67, s, 1H; H**9** = 13.50, s, 1H.	C**1** = 166.00; C**7** = 162.00.	388.0
**20**	1615	1555	1319-1150	H**8** = 8.52, s, 1H; H**9** = 12.56, s, 1H.	C**1** = 162.56; C**7** = 160.00.	407.0
**21**	1614	1562	1300-1155	H**8** = 8.18, s, 1H; H**9** = 12.31, s, 1H.	C**1** = 163.00; C**7** = 160.00.	456.0
**22**	1615	1558	1331-1152	H**8** = 8.12, s, 1H; H**9** = 12.46, s, 1H.	C**1** = 163.00; C**7** = 160.00.	470.0
**23**	1618	1570	1322-1152	H**8** = 8.37, s, 1H; H**9** = 13.47, s, 1H.	C**1** = 166.00; C**7** = 162.00.	436.0

Table 3: Some selected SCXRD data of *N*^{1}-(5^1-bromo-2^1-hydroxybenzylidenyl)-*N*-piperidinyl-2-sulfanilamide **18** and 4-chloro-*N*-[2-({(2E)-2-[(4-methoxyphenyl)methylidene]hydrazino} carbonyl)phenyl]benzene sulfonamide Y.

Bond Length (A°)	Compound **18**	Compound **Y**	Bond Length (A°)	Compound **18**	Compound **Y**
S1-O2	1.427	1.420	C1-N1	1.268	1.276
S1-O3	1.434	1.429	C1-C11	1.449	1.439
S1-N2	1.618	1.640			
S1-C22	1.767	1.759			

A summary of the physical properties and the spectroscopic data of compounds 17-23 are presented in Tables 1 and 2 respectively. This agrees with the results obtained by (Krátký *et al.,* 2017;

Govindaraj, Ramanathan, Rajendran, Ragu, Balachandaran, & Elanchleiyan, 2021;Elsamra, Masoud, & Ramadan, 2022).

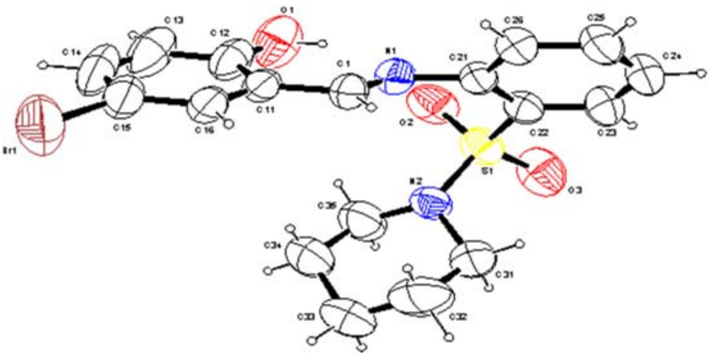

Figure 1: ORTEP diagram of N^{1}-(5^{1}-bromo-2^{1}-hydroxybenzylidenyl)-*N*-piperidinyl-2-sulfanilamide 18 determined by single crystal XRD. Ellipsoids were drawn at the 50% probability level.

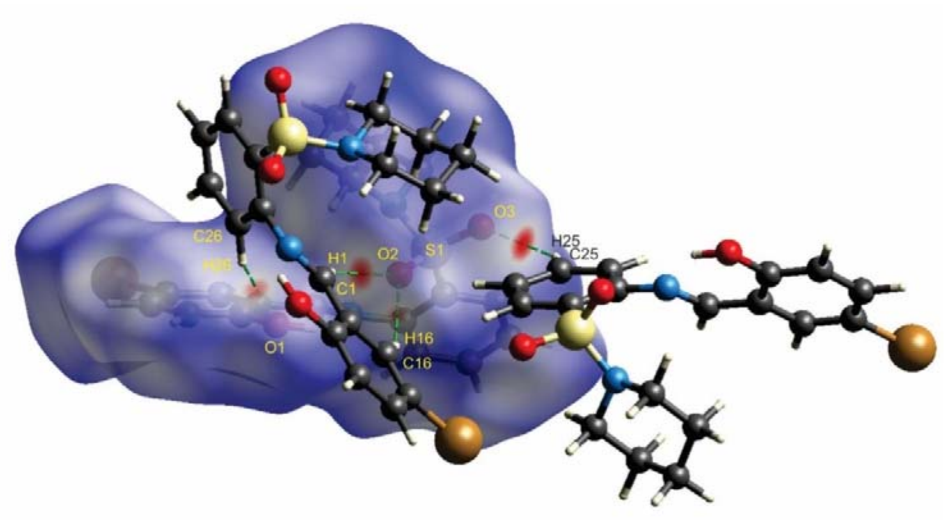

Figure 1: Hirshfeld surface of 18 plotted over d_{norm} showing selected intermolecular hydrogen interactions.

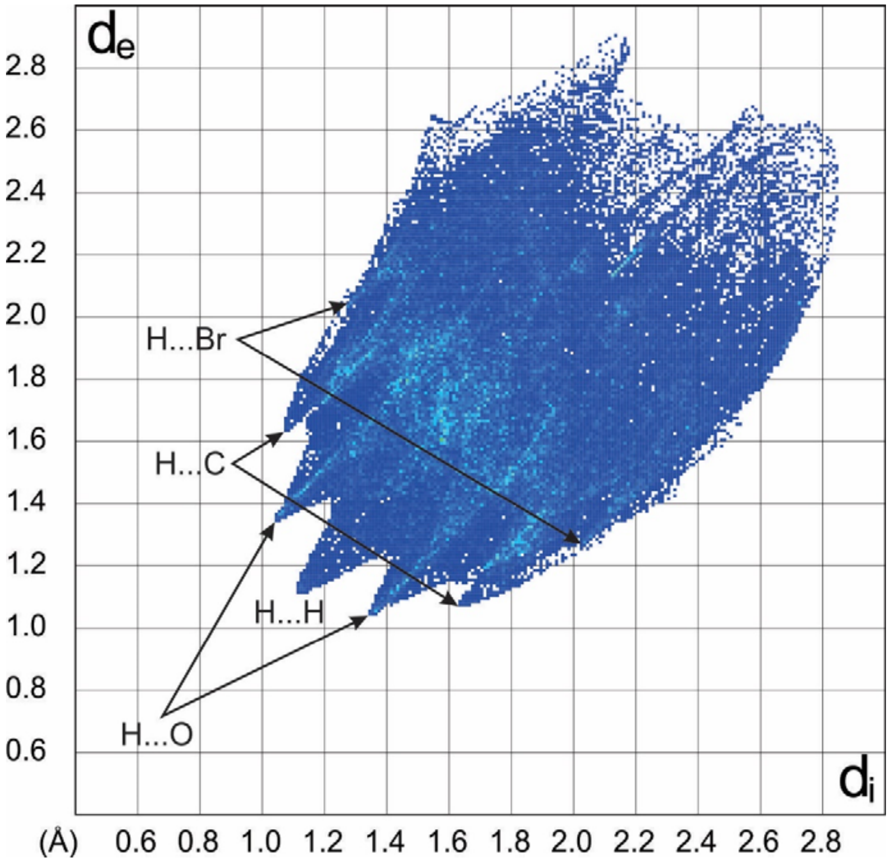

Figure 2: Hirshfeld surface fingerprint plot of 18 with indicated characteristic hydrogen interactions.

Table 3: Hirshfeld hydrogen reciprocal contact surface areas.

**Contact**	**Surface area %**
H…H	39.1
H…O/O…H	21.5
H…C/C…H	21.0
H…Br/Br…H	15.2

Table 14: Selected experimental bond lengths and bond angles

**Bond Length Experimental Å**	**Bond angle Experimental [°]**		
***N*^{1}-(5^1-bromo-2^1-hydroxybenzylidenyl)-*N*-piperidinyl-2-sulfanilamide**			
Br_1-C_{15}	1.893(6)	O_2-S_1-O_3	118.0(2)
S_1-O_2	1.427(4)	O_2-S_1-N_2	107.0(2)
S_1-N_2	1.618(4)	S_1-C_{22}-C_{23}	118.1(4)
S_1-C_{22}	1.767(4)	C_{21}-C_{22}-C_{23}	120.2(4)
**O_1-C_{12}**	1.343(7)	O_3-S_1-C_{22}	107.0(2)
**N_1-C_1**	1.268(5)	N_2-S_1-C_{22}	102.95(19)
**O_1-H_{1A}**	0.8200	C_1-N_1-C_{21}	119.8(4)
**C_1-C_{11}**	1.449(5)	C_{12}-O_1-H_{1A}	110.00
**C_{32}-C_{33}**	1.510(10)	C_{32}-C_{33}-C_{34}	109.4(5)
**C_1-H_1**	0.9300	Br_1-C_{15}-C_{16}	120.7(4)

Table 3 presents a detailed account of intramolecular interactions, encompassing D—H, H···A, D···A, D—H···A, and π···π interactions. Notably, the interaction between oxygen O_1 and hydrogen H_{1A}, labeled as O_1--H_{1A}..N_1, involves a hydrogen bond (D—H) at 0.8200 Å, an H···A distance of 1.8700 Å, a D···A distance of 2.585(6) Å, and a D—H···A angle of 145.00 °.

Table 15: Hydrogen-bonding geometry (Å)

Interactions	D—H (Å)	H···A (Å)	D···A (Å)	D—H···A (°)	π···π (Å)
**O_1-- H_{1A}..N_1**	0.8200	1.8700	2.585(6)	145.00	-
**C_1--H_1..O_2**	0.9300	2.5400	3.364(6)	14, 8.00	6_555
**C_{23}--H_{23}..O_3**	0.9300	2.4400	2.844(6)	107.00	-
**C_{25}--H_{25}..O_3**	0.9300	2.4900	3.219(6)	135.00	3_555
**C_{35}--H_{35B}..O_2**	0.9700	2.4300	2.857(7)	106.00	-
**Symmetry codes: (i)-1/2+x,y, 1/2-z, (ii) -1/2+x,1/2-y,-z, (iii) 1/2+x,y,1/2-z, (iv) 3/2-x,1/2+y,z, (v) x,1/2-y,1/2+z, (vi) x,1/2-y,-1/2+z, (vii) 3/2-x,1-y,-1/2+z, (viii) 1/2+x,1/2-y,-z, (ix) 3/2-x,-1/2+y,z, (x) 1-x,1-y,1-z, (xi) 3/2-x,1-y,1/2+z**					
